# The use of injectable orthobiologics for knee osteoarthritis: A formal ESSKA‐ORBIT consensus. Part 2—Cell‐based therapy

**DOI:** 10.1002/ksa.70001

**Published:** 2025-09-09

**Authors:** Laura de Girolamo, Giuseppe Filardo, Ferran Abat, Kristoffer Weisskirchner Barfod, Ricardo Bastos, Ramon Cugat, Michael Iosifidis, Baris Kocaoglu, Elizaveta Kon, Jeremy Magalon, Rodica Marinescu, Marko Ostojic, Mikel Sanchez, Thomas Tischer, Jasmin Bagge, Konrad Slynarski, Lucienne Vonk, Philippe Beaufils, Lior Laver, ESSKA‐ORBIT Group, Laura de Girolamo, Lior Laver, Giuseppe Filardo, Ferran Abat, Kristoffer Barfod, Ricardo Bastos, Ramon Cugat, Michael Iosifidis, Baris Kocaoglu, Elizaveta Kon, Jeremy Magalon, Rodica Marinescu, Marko Ostojic, Mikel Sanchez, Thomas Tischer, Diego Delgado, Patricia Laiz, Montserrat García, Jasmin Bagge, Konrad Slynarski, Lucienne Vonk

**Affiliations:** ^1^ Orthopaedic Biotechnology Laboratory IRCCS Ospedale Galeazzi Sant'Ambrogio Milano Italy; ^2^ Service of Orthopaedics and Traumatology, Department of Surgery EOC Lugano Switzerland; ^3^ Faculty of Biomedical Sciences Università Della Svizzera Italiana Lugano Switzerland; ^4^ Sports Orthopaedic Department, ReSport Clinic, Higher School of Health Sciences Tecnocampus, GRACIS Research Group (GRC 01604) Pompeu Fabra University Barcelona Spain; ^5^ Section for Sports Traumatology, Department of Orthopedic Surgery Copenhagen University Hospital – Bispebjerg and Frederiksberg Copenhagen Denmark; ^6^ Sports Orthopedic Research Center – Copenhagen (SORC‐C), Department of Orthopedic Surgery Copenhagen University Hospital – Hvidovre Copenhagen Denmark; ^7^ Hospital Lusíadas Santa Maria da Feira Santa Maria da Feira Portugal; ^8^ Fluminense Federal University Niterói Rio de Janeiro Brazil; ^9^ Instituto Cugat, Hospital Quironsalud Barcelona Barcelona Spain; ^10^ Fundación García Cugat, Mutualidad de Futbolistas Españoles‐Delegació Catalana Barcelona Spain; ^11^ OrthoBiology Surgery Center Thessaloniki Greece; ^12^ 3rd Orthopaedic Department European Interbalkan Medical Center Thessaloniki Greece; ^13^ Acibadem Altunizade Sports Therapy and Health Unit, Department of Orthopedics and Traumatology Acibadem University Faculty of Medicine Istanbul Turkey; ^14^ IRCCS Humanitas Research Hospital Rozzano Italy; ^15^ Department of Biomedical Sciences Humanitas University Pieve Emanuele Italy; ^16^ Cell Therapy Laboratory, Hôpital de la Conception, AP‐HM Marseille France; ^17^ INSERM, INRA, C2VN, Aix Marseille Univ Marseille France; ^18^ SAS Remedex Marseille France; ^19^ Department of Orthopaedics “Carol Davila” University of Medicine and Pharmacy Bucharest Romania; ^20^ Orthopedic Department Colentina Hospital Bucharest Romania; ^21^ Sports Traumatology Division, Traumatology Department Draskoviceva University Hospital “Sisters of Mercy” Zagreb Croatia; ^22^ Arthroscopic Surgery Unit Hospital Vithas Vitoria Vitoria‐Gasteiz Spain; ^23^ Advanced Biological Therapy Unit Hospital Vithas Vitoria Vitoria‐Gasteiz Spain; ^24^ Department of Orthopaedic Surgery University of Rostock Rostock Germany; ^25^ Mirai Clinic Warsaw Poland; ^26^ Department of Orthopaedics University Medical Center Utrecht Utrecht the Netherlands; ^27^ Xintela AB Lund Sweden; ^28^ ESSKA Consensus Projects Advisor Versailles France; ^29^ Department of Orthopaedics Hillel Yaffe Medical Center (HYMC), Hadera, Clinic Tel‐Aviv Israel; ^30^ Arthrosport Clinic Tel‐Aviv Israel; ^31^ Rappaport Faculty of Medicine Technion University Hospital (Israel Institute of Technology) Haifa Israel

**Keywords:** cell‐based therapy (CBT), intra‐articular injections, knee osteoarthritis, mesenchymal stromal cells (MSCs), orthobiologics

## Abstract

**Purpose:**

This European Society of Sports Traumatology, Knee Surgery and Arthroscopy (ESSKA) formal consensus aims to provide evidence‐ and expert opinion‐based recommendations for the use of point‐of‐care‐ and expanded‐cell‐based therapy (CBT) in the treatment of knee osteoarthritis (OA), focusing on indications, preparation, and administration.

**Methods:**

A multidisciplinary group of 77 leading experts in musculoskeletal regenerative medicine from 22 European Countries formed a steering group, a rating group, and a reader group. The steering group developed 23 questions, originating from 27 statements. The statements were graded from A (high‐level scientific evidence) to D (expert opinion). The question‐statement sets were scored by the rating group from 1 to 9 according to the level of agreement. The document was then assessed for geographic adaptability by the reader group composed of representatives from ESSKA‐affiliated societies.

**Results:**

Overall, the statements received a mean score of 8.2 (standard deviation: 0.3) points out of a possible 9 and a median score of 8 (range: 6–9). Among the 27 statements, 9 were considered appropriate with strong agreement, and 18 were considered appropriate with relative agreement. Five statements received a recommendation level of A or B, and 22 were rated as C or D. In terms of geographic adaptability, 18 affiliated ESSKA Societies expressed support, two were opposed, and two abstained. CBT has demonstrated consistent clinical benefits, particularly in pain and function improvement up to 12 months, supporting its use for patients with Kellgren–Lawrence (KL) Grades 1–3 knee OA, with some benefits, though inferior, in selected KL Grade 4. However, due to limited high‐quality studies and a lack of clear superiority over other injectables, CBT should be considered as a second‐line treatment option.

**Conclusions:**

The consensus document acknowledges ongoing debate about CBT's full effectiveness, though evidence suggests potential clinical benefits in pain relief and functional improvement. CBT are supported as a second‐line injectable treatment for KL 1–3 knee OA 1–3, with some benefits shown in Grade 4 too. However, gaps remain in high‐quality studies and treatment protocols. Nevertheless, evidence suggests that CBT may be seen as an alternative to traditional injectables like corticosteroids and hyaluronic acid, given the longer‐lasting benefits, and could currently be considered after other non‐operative treatment fails.

**Level of Evidence:**

Level I.

**Abbreviations**
BMA: bone marrow aspirate
BMAC: bone marrow aspirate concentrate
CBT: cell‐based therapy
CS: corticosteroids
HA: hyaluronic acid
IA: intra‐articular
KL: Kellgren–Lawrence
MFAT: microfragmented adipose tissue
OA: osteoarthritis
POC: point‐of‐care
PRP: platelet‐rich plasma
RCT: randomized controlled trial
SVF: stromal vascular fraction

## INTRODUCTION

Osteoarthritis (OA) affects over 500 million people worldwide, with an annual increase in cases due to the ageing population and an average annual cost per patient ranging from €0.7k to €10k [22]. The knee is the most affected joint in OA, and its treatment remains a topic of ongoing debate. While knee replacement is often the standard treatment for end‐stage OA, a variety of conservative management options exist for earlier stages, although these treatments do not always provide consistent or long‐term results. In this context, orthobiologics have gained significant attention as a promising treatment for tissue repair in various musculoskeletal conditions, both as a conservative injectable treatment and in combination with surgical procedures, where the former is becoming a central part of the non‐operative arsenal in the management of knee OA in many countries. Among orthobiologics, blood‐derived products, often and mainly indicated as platelet‐rich plasma (PRP) and cell‐based therapy (CBT), are the most used.

The first category is more widely recognized [6] and has been shown to be effective in the treatment of knee OA [24], while there is still a lack of agreement among professionals regarding patient indications, administration protocols, and the selection of specific CBT options. This issue warrants further attention, especially as the use of CBT has significantly increased in daily practice across many countries in recent years. Currently, the most commonly used CBT for knee OA includes injections of cells derived from bone marrow, adipose tissue and perinatal tissues. These treatments, which include both point‐of‐care (POC) minimally and mechanically manipulated cells and expanded cells, have shown promising disease‐modifying effects in various preclinical and in a few clinical studies [6, 10, 25, 29]. The mechanisms of action underlying these products are related to the presence of MSCs (mesenchymal stem/stromal cells or medicinal signalling cells), although in POC products, several other cell populations and tissue molecules can play a role. MSCs, originally of interest because of their ability to differentiate into cartilage, bone, and adipose tissue, are today recognized mainly for their paracrine activity. When injected into the affected joint, these cells promote tissue repair by secreting bioactive molecules, such as growth factors and cytokines, that reduce inflammation and, in turn, stimulate cartilage regeneration. MSCs also help modulate the immune response, improving the overall joint environment and restoring the correct homoeostasis. Several papers as well as systematic reviews and meta‐analyses on the use of CBT in the treatment of knee OA are available. While autologous treatments, using the patient's own cells, are generally preferred to minimize the risk of immune rejection, allogeneic treatments have garnered significant interest due to their regenerative and immunomodulatory potential, shorter treatment timelines, reduced costs, and elimination of donor‐site morbidity [26, 30]. Overall, the results are positive, although some inconsistencies are easily identified, such as variability in patient response and differences in treatment protocols across studies [8, 12, 14, 20, 23].

Despite the growing interest and promising results of CBT for knee OA, the field remains complex, with variations in indications, treatment protocols, cell sources, and delivery methods, making it challenging for clinicians to navigate the best approaches. A clear, evidence‐ and expert opinion‐based consensus would help standardize practices, ensure patient safety, and optimize therapeutic outcomes. It would also provide daily practitioners with a framework to make informed decisions, balancing the potential benefits of these therapies with the current limitations and uncertainties surrounding their use. For this reason, building on the success and insights gained from our first consensus document on PRP, the importance of extending this approach to cell‐based therapies was recognized. The European Society of Sports Traumatology, Knee Surgery and Arthroscopy (ESSKA), as Europe's largest association of musculoskeletal specialists, through the ORthoBIologics InitiaTive (ORBIT), has prepared this second formal consensus, aimed at providing a reference for the use of CBT. ORBIT strives to provide a clear framework for clinical practice, respecting different regulations on the use of cells in different countries, ensuring recommendations around the use of CBT injections, and facilitating informed decision‐making in this rapidly evolving field.

This manuscript specifically addresses CBT, either freshly prepared at the POC‐CBT or as expanded cell preparations prepared under the current good manufacturing practices (Expanded‐CBT). Following previous successful ESSKA formal consensus processes on various topics [2, 11, 13], the findings of this consensus offer a balanced combination of evidence‐based recommendations and clinical expertise. These recommendations aim to enhance decision‐making, refine indications, and improve the administration of CBT orthobiologics in the conservative approach to knee OA.

## MATERIALS AND METHODS

### Terminology

In this document the term CBT refers to a range of treatments that use living cells to promote tissue regeneration and/or repair, while modulating inflammation and supporting immune regulation. It includes both minimally manipulated autologous products prepared at the POC ‐ such as autologous bone marrow aspirate concentrate [BMAC], stromal vascular fraction [SVF] or microfragmented adipose tissue [MFAT] and allogeneic products derived from perinatal tissues (placenta, umbilical cord blood, amnitoic membrane) ‐ and culture‐expanded cells from the same sources.

### Consensus methodology

The consensus process for CBT for knee OA followed the ESSKA methodology for a formal consensus as described by the French National Healthcare Institution Haute Autorité de Santé HAS [18] as well as by ESSKA official organs [3, 4]. This approach included a group of experts in orthopaedics, cell biology, and tissue regeneration, working through phases. The process involved multiple stages, including online meetings, literature reviews, structured discussions, and voting rounds (detailed methodology can be accessed in [3, 4]. Specifically, the steering group, composed of 15 members (12 orthopaedic surgeons and 3 scientists) with expertise in the field of orthobiologics, developed a set of questions addressing various aspects of cell‐based therapies, including the rationale of use and the potential of both autologous and allogeneic cell therapies, either minimally manipulated or expanded cells, as well as standardizing protocols and appropriate cell sources. The questions, divided into three main sections, namely CBT rationale and indications (questions 1–12), CBT preparation and characterization (questions 13–18), and CBT protocol (questions 19–23), were prioritized based on clinical relevance and scientific importance using a decision‐making software (1000minds.com). After the question preparation, an independent subgroup of the steering group performed a comprehensive literature search in PubMed, Embase, and Cochrane databases. The search aimed to identify the best available scientific primary and secondary evidence on the topic published between 2000 and 2023 in the English Language. This literature group produced a specific literature summary for each question. Following the literature appraisal, draft statements were prepared by the steering group members, except those who performed the literature search. Each statement was assigned a grade of recommendation based on the Level of Evidence (LoE) of the existing literature on the topic according to international guidelines [16] with slight modifications. Specifically, A: high scientific level; B: scientific presumption; C: low scientific level; D: expert opinion.

All question‐statement sets with the related grade were thoroughly discussed by the steering group in collaboration with the consensus advisors—two scientists and a surgeon‐scientist specializing in musculoskeletal regenerative medicine. Once consensus was reached, the finalized question‐statement sets and accompanying literature summaries were circulated among the rating group, composed of 25 musculoskeletal experts, of whom most but not all were CBT users.

The rating process consisted of two rounds, during which the panel of experts assessed and ranked each statement using a discrete numerical scale (Likert scale from 1—indicating the *lowest level of agreement/entirely* inappropriate, to 9—indicating the *highest level of agreement/entirely* appropriate). A rating of 5 represented neutrality, so any score below this threshold was deemed inappropriate. After the first round of ratings, the text was revised by the steering group based on the feedback from the rating group, and a second iterative round of evaluation took place for the question‐statement sets, which had at least one score <5. Subsequently, a joint meeting of both the steering and rating groups was held to validate the draft and finalize the text. Each statement was assigned an agreement score (in the form of a median and range and mean ± standard deviation [SD]) that reflects a consensus among the raters on a particular statement A statement was deemed ‘appropriate’ when the median rating was ≥7 (strong agreement if scores ranged from 7 to 9, or relative agreement if scores ranged from 5 to 9). Conversely, a statement was classified as ‘uncertain’ when the median fell between 4 and 6.5, indicating indecision, or in any other case that did not meet the criteria for agreement or disagreement. Finally, a statement was considered ‘inappropriate’ if the median was ≤3.5 [18].

The final stage involved the reader group to evaluate the clarity, regional adaptability, and overall acceptance of the statements. A total of 41 affiliated societies from 36 European countries were reached out to by the ESSKA Office with a call for experts to assess the geographic adaptability of the document.

## RESULTS

The full consensus document, including the references used, is accessible on both the ESSKA and ESSKA Academy websites (https://esskaeducation.org/esska-consensus-projects) and is also available as Supporting Information S1: File 1.

Out of 23 questions, a total of 27 statements were developed, as some questions required two separate statements for proper consideration. In terms of grade of recommendation, 1 question/statement set was rated as Grade A, 4 as Grade B, 9 as Grade C, and 13 as Grade D (Table 1).

**Table 1:** Summary of the questions with grade of recommendation, mean ± SD and median and range of the rating group score and related grade of agreement.

Question	Question title	Grade of recommendation	Mean rating score (±SD)	Median rating score (range)	Grade of agreement (from rating scores)
1a	Does current evidence support the use of CBT for knee OA?—Point of care (POC)‐CBT	B	8.4 ± 0.8 (S1)	8.5 (6–9)	Appropriate with relative agreement
1b	Does current evidence support the use of CBT for knee OA?—Expanded‐CBT	A (S1)^a^ B (S2)	8.2 ± 0.9 (S1) 8.3 ± 1 (S2)	8 (6–9) 8.5 (5–9)	Appropriate with relative agreement Appropriate with relative agreement
2	For which degrees of knee OA is CBT recommended?	B	8.1 ± 1	8 (6–9)	Appropriate with relative agreement
3	Can CBT be used in severe knee OA (KL4)?	C	8.2 ± 0.8	8 (7–9)	Appropriate with strong agreement
4	Is there an age limitation for the use of CBT for knee OA?	D	8.2 ± 1	9 (6–9)	Appropriate with relative agreement
5	Could CBT for knee OA be used during the inflammatory phase when joint effusion is present (following effusion aspiration)?	D	8.1 ± 0.9	8 (6–9)	Appropriate with relative agreement
6	Are there specific contraindications for the use of CBT for knee OA?	D	8.0 ± 1	8 (5–9)	Appropriate with relative agreement
7	Does CBT induce disease‐modifying effects in knee OA?	C	8.4 ± 0.7	8.5 (7–9)	Appropriate with strong agreement
8	Is a repeated cycle of CBT injections recommended following a previous successful CBT treatment for knee OA upon the re‐emergence of symptoms?	D	8.2 ± 0.9	8 (6–9)	Appropriate with relative agreement
9	Are there advantages of CBT use in comparison to corticosteroids (CS) for treating knee OA?	D	8.3 ± 0.8	8 (6–9)	Appropriate with relative agreement
10	Are there advantages of CBT use in comparison to hyaluronic acid (HA) for treating knee OA?	B (S1) D (S2)	8.3 ± 0.8 (S1) 8.3 ± 1.0 (S2)	8 (6–9) 9 (5–9)	Appropriate with relative agreement Appropriate with relative agreement
11	Are there advantages of CBT use in comparison to platelet‐rich plasma (PRP) for treating knee OA?	C (S1) D (S2)	8.5 ± 0.9 (S1) 8.6 ± 0.6 (S2)	9 (5–9) 9 (7–9)	Appropriate with relative agreement Appropriate with strong agreement
12	Are there indications for the use of allogeneic cell products for knee OA?	C	7.8 ± 1.1	8 (6–9)	Appropriate with relative agreement
13	Is there a difference between bone marrow aspirate (BMA) and bone marrow aspirate concentrate (BMAC) for the management of knee OA?	D	8.5 ± 0.6	9 (7–9)	Appropriate with strong agreement
14	Is there a difference between mechanical stromal vascular fraction (SVF) of adipose tissue and microfragmented adipose tissue (MFAT) for the management of knee OA?	D	8.4 ± 0.6	8 (7–9)	Appropriate with strong agreement
15	Is there a clinical difference between expanded‐CBT and POC‐CBT for the management of knee OA?	C	8.0 ± 1.3	8 (5–9)	Appropriate with relative agreement
16	What should be the quality control measures for injectable CBT?	D	8.7 ± 0.5	9 (8–9)	Appropriate with strong agreement
17	Does harvest location, harvest equipment and number of trajectories matter for adipose tissue harvest and preparation?	D	8.4 ± 0.7	9 (7–9)	Appropriate with strong agreement
18	Does harvest location, harvest equipment and number of trajectories matter for bone marrow harvest and preparation?	C	8.3 ± 0.7	8 (7–9)	Appropriate with strong agreement
19	When using expanded mesenchymal stem/stromal cells (MSCs), what is the optimal/most appropriate number of cells to inject?	C	8.4 ± 1	9 (5–9)	Appropriate with relative agreement
20	For CBT Injections in knee OA—is one injection sufficient per treatment cycle?	C	8.4 ± 0.8	8 (6–9)	Appropriate with relative agreement
21	Are fasting or dietary restrictions recommended before CBT use? Could any other patient behaviour affect the treatment?	D	8.1 ± 1.1	8 (5–9)	Appropriate with relative agreement
22	Is antibiotics administration recommended around CBT use?	D	8.4 ± 0.8	9 (6–9)	Appropriate with relative agreement
23	Is there any clinical benefit of combining PRP with CBT?	C	8.4 ± 0.7	9 (7–9)	Appropriate with strong agreement

^a^ S1 (statement 1) and S2 (statement 2) are reported when more than one statement was produced for each question.

None of the proposed statements were considered inappropriate by the rating group, and therefore, none of the question/answer sets were deleted. After the second round of rating, overall, the statements received a mean score of 8.2 (SD: 0.3) points out of a possible 9 and a median score of 8 (range: 6–9). Among the 27 statements, 9 were considered appropriate with strong agreement, and 18 were considered appropriate with relative agreement.

Regarding the geographic adaptability, among the 36 contacted national societies, 22 affiliated ESSKA Societies responded to the call for experts to assess the geographic adaptability of the document. Of these, 18 Societies expressed support, two were opposed, meaning the consensus could not be applied in their countries, and two abstained, citing their inability to identify relevant experts on the topic within their membership (Table 2).

**Table 2:** Summary of ESSKA‐affiliated societies' positions on consensus adaptability to national contexts.

Country	ESSKA affiliated society	Geographic adaptability
Belarus	BAKAST	YES
Belgium	BKS	YES
Denmark	SAKS	NO
Estonia	EASTS	YES
France	SFTS	YES
Germany	DKG	YES
Germany – Austria ‐ Switzerland	AGA	YES
Great Britain	BASK	YES
Greece	HAA	YES
Hungary	MAT	YES
Israel	ISKSA	YES
Italy	SIAGASCOT	YES
Lithuania	LASTA	ABSTAINED
Luxembourg	LIROMS	YES
Norway	NAA	NO
Poland	PTA	YES
Portugal	SPAT	YES
Romania	SRATS	YES
Spain	SEROD	YES
Sweden	SFAIM	YES
The Netherlands	NVA	ABSTAINED
Turkey	TUSYAD	YES

### Complete list of questions and statements

*IMPORTANT: All the statements are based on the combination of clinical expertise and the current existing literature findings. As such, the recommendations regarding POC‐CBT refer only to those obtained by medical devices that have been clinically tested and appropriately studied in the literature*.

## SECTION 1 — CBT RATIONALE AND INDICATIONS

### QUESTION 1

#### Does current evidence support the use of CBT for knee OA?

##### POC‐CBT

Current scientific evidence has shown that the use of POC‐CBT products for knee OA can provide clinical benefit and is a safe treatment option, although certain limitations of current evidence exist due to the heterogeneity of products and the lack of studies on larger populations. Clinical improvement has been shown at both shorter (6 months) and longer (12 months) durations in most of the studies available in the literature. **The consensus group, therefore, concludes that there is sufficient clinical evidence to support the use of POC‐CBT as a treatment option for knee OA** (see following questions addressing CBT specifications and indications).

However, due to the lack of sufficient high‐quality studies in larger populations, as well as the lack of superiority in some studies compared to corticosteroids (CS) or PRP injections, the full clinical benefit and role of POC‐CBT products in the treatment algorithm for knee OA is not completely understood and, as such, **the consensus group currently does not recommend the use of POC‐CBT as a first‐line injectable treatment for knee OA**. The consensus group, therefore, does agree that CBT could be considered when other non‐operative and other injectable measures have failed and in circumstances where surgery is not yet indicated or medically appropriate.

Grade of recommendation: B

Rating score: Mean 8.4 ± 0.8; Median 8.5 (6–9) Appropriate with relative agreement

##### Expanded‐CBT

Current scientific evidence supports the clinical benefit/efficacy and safety of **Expanded‐CBT** for knee OA, confirming the findings of preclinical research. Clinical improvement has been shown at both shorter (6 months) and longer (up to 24 months) durations in most of the published studies. **The consensus group, therefore, concludes that there is sufficient preclinical and clinical evidence to support the use of Expanded‐CBT as a treatment option for knee OA** (see following questions addressing Expanded‐CBT specifications and indications) when regulatory approval exists.

Grade of recommendation: A

Rating Score: Mean 8.2 ± 0.9; Median 8 (6–9) Appropriate with relative agreement

Due to the complexity of the preparation procedure of autologous Expanded‐CBT products, **the consensus group currently does not recommend the use of Expanded‐CBT as a first‐line injectable treatment for knee OA.** The consensus group agrees that **Expanded‐CBT** could be considered when other non‐operative and other injectable measures have failed and in circumstances where surgery is not yet indicated or not medically appropriate.

Grade of recommendation: B

Rating Score: Mean 8.3 ± 1.0; Median 8.5 (5–9) Appropriate with relative agreement

### QUESTION 2

#### For which degrees of knee OA is CBT recommended?

Current evidence has shown the clinical benefit of CBT in knee OA Kellgren–Lawrence (KL) Grades 1–4; however, most studies involved populations with KL Grades 2 and 3.

**The consensus group recommends that CBT can be used for knee OA, mainly in Grades 1–3, although clinical benefits have also been shown in KL Grade 4.**

Grade of recommendation: B

Rating Score: Mean 8.1 ± 1.0; Median 8 (6–9) Appropriate with relative agreement

*This statement is valid for both POC‐ and Expanded‐CBT*.

### QUESTION 3

#### Can CBT be used in severe knee OA (KL 4)?

In comparison to KL Grades 1–3, although roughly half of the studies include patients with KL 4, the evidence on CBT use for KL 4 is more scarce, given the lower number of patients presenting with this advanced OA grade. Nevertheless, the evidence does show that CBT can also be used in severe knee OA (KL 4), with clinical benefits lasting up to 12 months. **Therefore, the consensus group suggests that CBT could be considered as a treatment option in severe knee OA (KL 4),** especially in individuals who have failed other non‐operative strategies (including other injectable therapies) and who are not yet indicated for or are not willing to undergo knee replacement surgery or cannot undergo such surgery due to comorbidities. **However, the consensus group also recommends informing such patients that outcomes may not be as favourable or may not last as long as for lower grades of knee OA.**

Grade of recommendation: C

Rating Score: Mean 8.2 ± 0.8; Median 8 (7–9) Appropriate with strong agreement

*This statement is valid for both POC‐ and Expanded‐CBT*.

### QUESTION 4

#### Is there an age limitation for the use of CBT for knee OA?

There is insufficient evidence in the literature regarding an age limitation for CBT use in knee OA. The majority of available studies on CBT use for knee OA include patients between 18 and 75 years of age. However, several in vitro studies have shown/suggested a decline in stem cell quantity and quality with increasing donor age, where adipose tissue‐derived mesenchymal stem/stromal cells (MSCs) seem to be less affected by donor age compared to bone marrow‐MSCs.

**Current evidence does not indicate clear limitations or associations with regard to cell quantity/cell viability and product efficacy. Therefore, the consensus group agrees that a specific age range cannot be recommended.** In light of the limited evidence on the age factor, the consensus group suggests that treatment decisions should not be based only on chronologic age as other factors should come into consideration, **though it recognizes that there is evidence suggesting a reduced cell quantity and possibly quality with age**.

Grade of recommendation: D

Rating Score: Mean 8.2 ± 1.0; Median 9 (6–9) Appropriate with relative agreement

*This statement is valid for both POC‐ and Expanded‐CBT*.

### QUESTION 5

#### Could CBT for knee OA be used during the inflammatory phase when joint effusion is present (following effusion aspiration)?

Current clinical evidence is lacking regarding the injection of CBT during the inflammatory phase in knee OA, as well as with regard to effusion aspiration prior to CBT injection.

However, multiple basic science studies have shown anti‐inflammatory properties of CBT, which could support the rationale for its use during the inflammatory phase. While evidence is lacking with regard to the optimal timing of CBT injection for knee OA when effusion is present, **the consensus group recognizes that when present, effusion aspiration is likely beneficial in pain improvement and relieving functional limitations and also avoids dilution of the injected CBT product.** In addition, it could aid in replacing the inflammatory infiltrate in the knee with a more favourable one with anti‐inflammatory properties.

Grade of recommendation: D

Rating Score: Mean 8.1 ± 0.9; Median: 8 (6–9) Appropriate with relative agreement

*This statement is valid for both POC‐ and Expanded‐CBT*.

### QUESTION 6

#### Are there specific contraindications for the use of CBT for knee OA?

Apart from the generally accepted contraindications for any knee injection, the consensus group attempted to identify and highlight specific contraindications related to CBT injections. In the absence of formal contraindications documented in the existing literature for the application of CBT in the treatment of knee OA, the consensus group's focus has been directed towards the identification of exclusion criteria employed in clinical trials with a level of evidence I. These identified exclusion criteria provide essential guidance when considering the suitability of patients for CBT in the treatment of knee OA. **The consensus group agrees that caution should guide any decision‐making process when considering the use of CBT, helping to ensure patient safety as well as the appropriate selection of candidates for clinical interventions.** This is relevant in conditions such as pregnancy, in which there's no available evidence on the safety of CBT use; therefore, the consensus group recommends avoiding its use in these instances until further data is available.

Since most of the suggested contraindications have not been thoroughly or sufficiently studied, the consensus group chose to recommend caution also in the presence of co‐existent malignancies or systemic conditions due to the possibility of unknown interactions.

While it remains challenging to ascertain whether these exclusion criteria are specific to CBT, the most frequently described criteria for exclusion from these clinical trials were categorized based on locally related contraindications, harvest site‐associated contraindications, and systemic‐related contraindications (for more details, see Supporting Information S1: File 1).

Grade of recommendation: D

Rating Score: Mean 8.0 ± 1.0; Median 8 (5–9) Appropriate with relative agreement

*This statement is valid for both POC‐ and Expanded‐CBT*.

### QUESTION 7

#### Does CBT induce disease‐modifying effects in knee OA?

Clinical evidence on the disease‐modifying effects of CBT for knee OA derived from adipose tissue, bone marrow, and foetal annexes (placenta, amnion, umbilical cord/umbilical cord blood) remains limited and inconsistent. Preclinical studies provide promising insights into their potential for disease modification in knee OA. A large number of animal studies show disease‐modifying effects of CBT derived from the aforementioned tissue sources in animal OA models. Specifically, positive results have been reported in macroscopic, histological, and immunohistochemical analyses at both cartilage and synovial levels, with superior effects associated with adipose‐derived products over the other two. **However, currently, there is no sufficient direct evidence to suggest that CBT induces disease‐modifying effects in humans.**

Grade of recommendation: C

Rating Score: Mean 8.4 ± 0.7; Median 8.5 (7–9) Appropriate with strong agreement

*This statement is valid for both POC‐ and Expanded‐CBT*.

### QUESTION 8

#### Is a repeated cycle of CBT injections recommended following a previous successful CBT treatment for knee OA upon the re‐emergence of symptoms?

Current evidence regarding repeated cycles of CBT treatment for knee OA is limited. However, it has been suggested that this strategy may have clinical benefits. **As evidence suggests a decrease in the effects of CBT for knee OA over time, the consensus group agrees that an additional cycle could be considered upon the re‐emergence of symptoms after a previous successful treatment (lasting around 12 months).** However, the available data are mainly related to autologous procedures. Concerning allogeneic procedures, a few preclinical studies have indicated a local immune response with potential destruction of injected MSCs, while a few clinical studies have indicated a potential higher risk of mild local adverse events when using allogeneic cells for repeated treatments.

Grade of recommendation: D

Rating Score: Mean 8.2 ± 0.9; Median 8 (6–9) Appropriate with relative agreement

*This statement is valid for both POC‐ and Expanded‐CBT*.

### QUESTION 9

#### Are there advantages of CBT use in comparison to CS for treating knee OA?

**Although the literature is sparse with regard to direct comparisons between CBT and CS, the current available evidence does not show the clinical superiority of CBT compared to CS**. However, CS injections have been shown to have detrimental effects on chondrocytes and can lead to accelerated cartilage degeneration, especially with multiple/repeated injections. Although CS are strong anti‐inflammatory agents and can provide short‐term relief in knee OA (mainly less than 3 months), CBT injections have been shown to have the potential for a longer effect in comparison to the shorter‐term effect of CS injections. CBT also seems to provide a safer use profile with fewer potentially related complications compared to CS, especially when considering the potential need for repeated injections in knee OA patients, more so in younger patients. Therefore, **the consensus group considers CBT injections to be a non‐chondro‐toxic and effective treatment option, with potentially expected longer‐term clinical improvements compared to CS injections.**

Grade of recommendation: D

Rating Score: Mean 8.3 ± 0.8; Median 8 (6–9) Appropriate with relative agreement

*This statement is valid for both POC‐ and Expanded‐CBT*.

### QUESTION 10

#### Are there advantages of CBT use in comparison to hyaluronic acid (HA) for treating knee OA?

Several high‐level studies, as well as meta‐analyses, exist comparing the effectiveness of CBT to HA for knee OA, with the majority favouring CBT in terms of overall clinical improvement and a longer‐lasting effect documented to last up to 12 months.

**Based on current available evidence, the consensus group acknowledges that CBT seems to have superiority over HA for knee OA due to overall clinical improvement and expected longer‐lasting effects,** whilst also acknowledging that there are different formulations of the products that may introduce some bias in the conclusions of meta‐analyses.

Grade of recommendation: B

Rating Score: Mean 8.3 ± 0.8; Median 8 (6–9) Appropriate with relative agreement

However, due to the more invasive and complex preparation process of CBT, **the consensus group recommends that its use should be reserved as a second‐line injectable treatment option.**

Grade of recommendation: D

Rating Score: Mean 8.3 ± 1.0; Median 9 (5–9) Appropriate with relative agreement

*These statements are valid for both POC‐ and Expanded‐CBT*.

### QUESTION 11

#### Are there advantages of CBT use in comparison to PRP for treating knee OA?

Current literature regarding the advantage or superiority of CBT compared to platelet‐rich plasma (PRP) is limited and inconclusive, with few studies performed with direct comparisons between CBT and PRP. Therefore, based on current evidence, **the consensus group does not acknowledge superiority or clear advantages of CBT over PRP for knee OA.**

Grade of recommendation: C

Rating Score: Mean 8.5 ± 0.9; Median 9 (5–9) Appropriate with relative agreement

Moreover, considering the relatively invasive and more complex nature of the preparation procedure of CBT compared to PRP, **the consensus group recommends that PRP should be used as a first‐line orthobiologic injectable treatment option in knee OA, while CBT could be considered as a second‐line orthobiologic treatment option.**

Grade of recommendation: D

Rating Score: Mean 8.6 ± 0.6; Median 9 (7–9) Appropriate with strong agreement

*These statements are valid for both POC‐ and Expanded‐CBT*.

### QUESTION 12

#### Are there indications for the use of allogeneic cell products for knee OA?

While several high‐quality studies exist evaluating the clinical benefit of allogeneic CBT for knee OA with promising results in terms of clinical benefit, current evidence is still limited due to relatively small sample sizes and heterogeneity in cell preparation and dosing, product characterization, and patient populations. Additionally, the optimal source, dose, and frequency of MSC injections have not been established. Some transient adverse effects have been reported in preclinical studies, especially after repeated injections, as well as in a few clinical studies, often comparable to those observed with the use of autologous cells. **The consensus group cannot suggest that allogeneic CBT have an advantage over autologous CBT in terms of clinical benefit, although the lack of donor‐site morbidity could play an important role in some subjects. Therefore, the consensus group suggests allogeneic CBT could be considered in patients who cannot have autologous CBT for any reason or in whom autologous CBT is contraindicated.**

*(For contraindications to the use of CBT, please refer to Question 6; for effects of repeated CBT injections, please refer to Question 8)*

Grade of recommendation: C

Rating Score: Mean 7.8 ± 1.1; Median 8 (6–9) Appropriate with relative agreement

*These statements are valid for both POC‐ and Expanded‐CBT*.

## SECTION 2 — CBT PREPARATION AND CHARACTERIZATION

### QUESTION 13

#### Is there a difference between bone marrow aspirate (BMA) and BMAC for the management of knee OA?

Current evidence is lacking controlled clinical studies directly comparing BMA and BMAC for the management of knee OA. Nevertheless, data indicates that BMA obtained with the most appropriate instruments and technique provides a similar number of bone marrow mesenchymal stem/stromal cells (BM‐MSCs) as in single‐spin BMAC from a sample harvested without specific techniques aimed at minimizing peripheral blood contamination. When using the same equipment and technique for bone marrow harvesting, BMAC (obtained by centrifugation) will result in a product with a higher cell number, although with a lower volume. **The consensus, therefore, agrees it is essential to adopt the most suitable technique and instrument for bone marrow collection (see Question 18) in order not to compromise the resulting product or concentration procedure when relevant.**

A double‐spin BMAC protocol is reported to increase the cell concentration while significantly reducing the volume. Double‐spin BMAC protocol produces a higher BM‐MSCs number, which seem to positively influence clinical benefit and, therefore, when considering BMAC use for knee OA, the consensus group recommends the use of products with a double‐spin protocol.

Grade of recommendation: D

Rating Score: Mean 8.5 ± 0.6; Median 9 (7–9) Appropriate with strong agreement

### QUESTION 14

#### Is there a difference between the mechanical SVF of adipose tissue and MFAT for the management of knee OA?

Although different in composition and structure, mechanical SVF and MFAT show a similar safety and efficacy profile for the treatment of knee OA, with satisfactory subjective results up to 24 months. Until further studies are conducted to determine whether one product is clinically superior to the other, **the consensus group currently does not support one type of adipose‐derived CBT over the other and considers both mechanical SVF and MFAT valid options for the management of knee OA when this approach is considered.**

Grade of recommendation: D

Rating Score: Mean 8.4 ± 0.6; Median 8 (7–9) Appropriate with strong agreement

### QUESTION 15

#### Is there a clinical difference between expanded‐CBT and POC‐CBT for the management of knee OA?

The literature involving direct comparisons between expanded‐CBT and POC‐CBT is sparse and limited. Treatments involving both expanded cells and POC products have been shown to be safe treatment options and to have the ability to provide clinical benefit for up to 12–24 months.

Expanded cell products have been shown to provide more consistent cell numbers, although they entail a higher production cost and a more complex two‐stage procedure (in autologous products). Discrepancies in the clinical settings, in production protocols, and the lack of stratification of OA patients based on the radiologic classification currently limit any recommendation on the use of either product group in clinical practice. Therefore, **the consensus group does not recommend the use of one group over the other and currently considers both expanded‐CBT and POC‐CBT as acceptable products for the management of knee OA.**

Grade of recommendation: C

Rating Score: Mean 8.0 ± 1.3; Median 8 (5–9) Appropriate with relative agreement

### QUESTION 16

#### What should be the quality control measures for injectable CBT?

Currently available literature on quality control measures for CBT is variable, spanning from a lack of information—mainly for POC products—to exhaustive biological characterization—mainly for expanded cells.

For improving clinical practice and the quality of future studies as well as enabling improved comparison measures between studies and products, **the consensus group considers the reporting of cell characterization an important minimum quality control requirement**. The consensus group suggests adopting the ‘Minimum Information for Studies Evaluating Biologics in Orthopaedics (MIBO) requirements’ when reporting data about CBT.

Grade of recommendation: D

Rating Score: Mean 8.7 ± 0.5; Median 9 (8–9) Appropriate with strong agreement

*This statement is valid for both POC‐ and Expanded‐CBT*.

### QUESTION 17

#### Does harvest location, harvest equipment and number of trajectories matter for adipose tissue harvest and preparation?

Although in vitro studies show differences in terms of cell yield and performance among different anatomic harvest sites for adipose tissue, due to the lack of stringency and high heterogeneity in the design of the available clinical studies, **currently, it is not possible to recommend one harvest site over another**. Due to the different body mass composition of athletes, adipose tissue harvesting can be more complicated in these patients. **The consensus group, therefore, suggests choosing preparation methods that require a smaller volume of adipose tissue**, or, in case of scarce material, to combine adipose tissue‐derived products with PRP or other orthobiologics.

There is a general trend that mild harvest methods, such as surgical resection and manual lipoaspiration, are milder in terms of cell integrity, but due to the lack of clinical data comparing possible different methods, **it is not possible to draw any clear conclusion with regard to the optimal harvest equipment.**

Finally, no studies investigated the effect of the number of harvesting trajectories on adipose tissue CBT related to knee OA treatment and, therefore, **the consensus group cannot recommend an optimal number of harvest trajectories.**

Grade of recommendation: D

Rating Score: Mean 8.4 ± 0.7; Median 9 (7–9) Appropriate with strong agreement

*This statement is valid for both POC‐ and Expanded‐CBT*.

### QUESTION 18

#### Does harvest location, harvest equipment and number of trajectories matter for bone marrow harvest and preparation?

Although clinical studies comparing different harvest locations for bone marrow harvesting are lacking, **the posterior and anterior iliac crests have been suggested as the best sites for collecting bone marrow for intra‐articular injection because of the highest number of MSCs available in comparison to other sites**.

Clinical studies directly comparing different harvesting equipment are also lacking. Nevertheless, to improve the quality of bone marrow, that is, the lack of peripheral blood contamination, **the consensus group recommends performing multiple puncture sites and different trajectories and gradual advancements to harvest small volumes of BMA** (up to 2–5 mL from each location) from multiple sites with a 10 mL syringe.

Grade of recommendation: C

Rating Score: Mean 8.3 ± 0.7; Median 8 (7–9) Appropriate with strong agreement

*This statement is valid for both POC‐ and Expanded‐CBT*.

## SECTION 3 — CBT PROTOCOL

### QUESTION 19

#### When using expanded MSCs, what is the optimal/most appropriate number of cells to inject?

The majority of available dose‐response studies reported the use of <100 × 10^6^ MSCs. However, due to the lack of stringency and high heterogeneity in the design of the available studies and due to the absence of a clear correlation between cell numbers and clinical outcomes, as well as various cell numbers in different studies, currently no consensus exists about the most appropriate number of expanded MSCs to inject in the treatment of knee OA. **The consensus group concludes that defining the optimal MSC number for the management of knee OA is complex and includes many variables, and, therefore, currently, optimal cell ranges for the treatment of knee OA cannot be defined.**

Grade of recommendation: C

Rating Score: Mean 8.4 ± 1.0; Median 9 (5–9) Appropriate with relative agreement

### QUESTION 20

#### For CBT Injections in knee OA—Is one injection sufficient per treatment cycle?

Current literature is scarce with regard to the optimal number of CBT injections per treatment cycle for the management of knee OA. To date, no study involving autologous POC‐CBT includes more than one injection protocol, whereas a few studies using expanded MSCs reported the outcomes of multiple injections in a short interval. Although studies using expanded cells with more than one‐injection protocols have shown to provide clinical benefit, there is a lack of sufficient data to support multiple injection protocols over single‐injection protocols and, therefore, **the consensus group cannot recommend one protocol over the other for either POC‐CBT or expanded‐CBT for the management of knee OA.**

Grade of recommendation: C

Rating Score: Mean 8.4 ± 0.8; Median 8 (6–9) Appropriate with relative agreement

*This statement is valid for both POC‐ and Expanded‐CBT*.

### QUESTION 21

#### Are fasting or dietary restrictions recommended before CBT use? Could any other patient behaviour affect the treatment?

The literature lacks clinical data regarding the direct impact of diet and fasting or other lifestyle recommendations on the therapeutic effects of CBT. However, since basic science literature reports negative effects of these behaviours on MSCs' performances, the consensus group acknowledges the importance of adopting some dietary and life restrictions (including alcohol consumption and smoking habit) a few weeks before the treatment in case of autologous treatment to maximize the efficacy. Moreover, given the presence of peripheral blood contamination in most of the POC‐CBT (mainly cone marrow concentrate and SVF), similar recommendations to those for the use of PRP should be followed. **The consensus group, therefore, recommends that patients avoid high‐fat foods for at least 24 h prior to a CBT treatment.**

Grade of recommendation: D

Rating Score: Mean 8.1 ± 1.1; Median 8 (5–9) Appropriate with relative agreement

*This statement is valid for both POC‐ and Expanded‐CBT*.

### QUESTION 22

#### Is the administration of antibiotics recommended around CBT use?

**Evidence on the administration of antibiotics around CBT use is lacking. Therefore, the consensus group does not recommend the routine use of antibiotics around CBT use.** However, unlike other injectable products for the knee joint, the autologous CBT preparation process involves tissue harvesting (mainly but not only fat or bone‐marrow) and therefore some degree of infectious risk should be taken into consideration. To reduce the infectious risk, **the consensus group recommends performing CBT procedures in an appropriate and dedicated environment** (i.e., sterile office area, operating theatre or similar environments). Nevertheless, the consensus group suggests taking a cautious approach in specific cases and considering the administration of antibiotics in populations with higher risk factors for infections, such as diabetics, heavy smokers, previous joint infections, or wound complications.

Grade of recommendation: D

Rating Score: Mean 8.4 ± 0.8; Median 9 (6–9) Appropriate with relative agreement

*This statement is valid for both POC‐ and Expanded‐CBT*.

### QUESTION 23

#### Is there any clinical benefit of combining PRP with CBT?

Current pre‐clinical and clinical literature suggests some potential benefits of combining PRP with cell‐based products, with the majority of studies focusing on culture‐expanded cells. However, evidence is still lacking regarding the clear benefits of using these products in combination over using CBT alone. **Therefore, based on current evidence, the consensus group does not see clear advantages from combining PRP with CBT products for knee OA and does not recommend a combined treatment.**

Grade of recommendation: C

Rating Score: Mean 8.4 ± 0.7; Median 9 (7–9) Appropriate with strong agreement

*This statement is valid for both POC‐ and Expanded‐CBT*.

## DISCUSSION

This ESSKA‐ORBIT formal consensus on the injectable use of CBT for the non‐surgical treatment of knee OA aims to guide clinicians through the complexities of CBT application. It also provides a comparative analysis of CBT versus other injectable therapies, helping to clarify its role in managing knee OA. The document is organized into three key sections: indications and appropriateness of CBT, variability in product formulations and protocols, and safety and administration considerations.

The most important messages of this document are that, given the consistent results showing that CBT can yield significant clinical benefits, including improvements in pain and function, particularly over periods ranging from 6 to 12 months, the consensus results suggest there is sufficient clinical evidence to support the use of both POC‐CBT and Expanded‐CBT as a treatment option for patients with KL Grades 1–3 knee OA.

**Figure 1:**
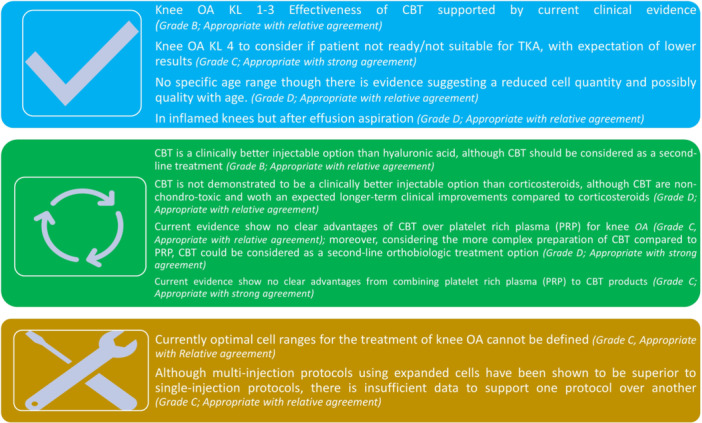
A summary of the main messages of the ESSKA formal consensus on the use of injectable CBT for knee osteoarthritis. CBT, cell‐based therapy.

However, due to the lack of sufficient high‐quality studies in larger populations, as well as the lack of superiority in some studies compared to CS injections or PRP, the consensus group currently does not recommend the use of POC‐CBT as a first‐line injectable treatment for knee OA, nor as a first‐line orthobiologic injectable treatment option. Moreover, the project output indicates that CBT could be considered when other non‐operative and other injectable measures have failed. In this regard, some clinical improvements have also been observed in KL Grade 4. However, the group emphasized that in severe OA, CBT could be considered mainly for those who have not responded to other non‐operative measures and who are not yet eligible for surgery, warning patients that the results may not be as favourable or may not last as long as for lower grades of knee OA (Figure 1).

A major point of discussion is the appropriate age range for this kind of therapy. The literature does not provide sufficient elements to establish clear age limitations for the use of CBT in knee OA, suggesting that treatment decisions should, therefore, be based on a broader clinical assessment rather than chronological age alone. However, since it is known that older patients may experience a decrease in stem cell quality and quantity, particularly with BM‐derived MSCs, it would be advisable to consider these treatments more carefully in older patients, although there is no definitive evidence linking age to treatment outcomes.

CBT, especially POC‐CBT, have emerged as an alternative to more established injectable treatments such as CS, HA, and also blood‐derived products. Although no direct evidence supports the superiority of CBT over CS, several key points were raised. First, CBT is considered non‐chondrotoxic. This is a significant advantage over CS, which can accelerate cartilage degeneration with repeated use, as it is expected over the course of OA progression. Furthermore, CBT may offer longer‐lasting benefits than CS and, therefore, should be preferred, especially in younger patients. When comparing CBT to HA, evidence supports both POC‐CBT and Expanded‐CBT as potentially superior in terms of clinical improvement and duration of effect [1, 5, 9, 15, 17, 19, 31]. However, the more invasive and complex preparation of CBT cannot be ignored and, in this context, the consensus group recommends it should be reserved for second‐line therapy, with HA remaining a first‐line option. In terms of PRP, the evidence is less conclusive, and while no clear advantage of CBT over PRP has been identified, the consensus group suggests that CBT could be considered when PRP fails, given its broader clinical benefit in some cases. It is important to note that all these comparisons are affected by the heterogeneity in CBT and PRP preparations, as well as the variability in the characteristics of HA products reported in the literature.

The use of allogeneic CBT products (derived from donors) for knee OA was also addressed in this consensus. While there are promising results from preclinical and clinical studies, the consensus group noted that evidence remains limited, with small sample sizes and variations in preparation and dosing. Allogeneic CBT may be an option for patients who cannot undergo autologous treatments, although at the moment it does not appear to offer a clear clinical advantage over autologous CBT. Moreover, a few studies implying repeated cycles of allogeneic CBT injection have raised concerns regarding immune response and local adverse events, although reported adverse events were mild and self‐resolving.

Despite some challenges, such as variability in the clinical outcomes linked to different preparation protocols and product characteristics, the consensus group was able to reach a high level of agreement on all the statements, with 9 statements receiving a strong agreement and 18 a relative agreement. Interestingly, the overall results of this document are quite similar to the previous consensus document prepared by the same working group on the use of injectable blood‐derived products for the treatment of knee OA [21]. In fact, the consensus document on PRP overall showed a mean score of 8.2 (SD: 0.3) and a median score of 9 (range: 6–9), with 16 out of 31 statements considered appropriate with strong agreement and 15 appropriate with relative agreement. The similarity between the two consensus documents, despite the slightly stronger agreement seen in the PRP document, can be explained by the growing body of evidence supporting the effectiveness of both cell‐based therapies and injectable blood‐derived products for treating knee OA. While PRP has garnered more robust consensus likely due to its longer history of clinical use and more established protocols, the overall agreement on cell‐based therapies reflects the increasing confidence in these treatments as viable alternatives in most European countries. The high level of agreement across both documents suggests that, even though there are variations in clinical outcomes depending on the specific product or protocol, experts are aligning on the overall potential of these therapies for knee OA.

### Strengths and limitations of the consensus document

Several factors are crucial in order to generate a high‐quality consensus. The first key factor is assembling a leading group of experts in the relevant specific field. Another key factor is maintaining strict adherence to a well‐defined and validated methodology throughout the consensus process [3, 18, 28] to reach a high consensus level through an iterative process necessary to ensure increasing agreement. An additional strength of the current consensus stems from its pan‐European geographical representation, involving 77 clinicians and scientists from a total of 22 European countries, supporting it as a reliable depiction of the European position on this topic. However, it is also important to acknowledge the differences in healthcare systems across different countries, which may influence the daily practice, clinical decision‐making, and use of injectable CBT for the management of knee OA and, therefore, may not always be in line with the current scientific evidence. Therefore, it is important to note that the statements and recommendations in the current consensus do not represent absolute values or standalone binding parameters and should be interpreted and used in a critical manner in combination with clinical evaluation and other objective assessment tools when generating a treatment plan for each patient individually.

There are several limitations to the current consensus. It is undisputed that the strength of a consensus relies not only on the methodology used, but also on the level of evidence available with regard to the addressed topics. A consensus may typically include aspects with high‐level and others with low‐level scientific evidence. When scientific evidence is lacking or insufficient, clinical expertise is utilized to provide the best available recommendation. However, while high‐level meta‐analyses and systematic reviews include only high‐level scientific evidence, they are often not able to provide answers and address the questions and issues encountered by daily practitioners. This is the scope of a consensus: it provides the best practical recommendation available to practitioners, even if the level of available evidence is low for a certain question, and this will be represented by a low grade of recommendation. When critically assessing a consensus, it is often mistaken to rely only on available evidence. This is not the scope of a consensus. A consensus applies similar principles, which serve as the pillars of evidence‐based medicine (EBM). In its original definition, ‘EBM integrates individual clinical expertise with the best available external clinical evidence from systematic research and the compassionate use of individual patients' predicaments, rights, and preferences. By individual clinical expertise, we mean the proficiency and judgement that individual clinicians acquire through clinical experience and practice’ [27]. EBM is thus a complex concept that is often wrongly perceived as exclusive to scientific studies, focusing solely on the level of evidence for each study, whereas scientific evidence is only a part of EBM. Similarly, the consensus integrates scientific evidence and clinical expertise, and its value is even more relevant when the scientific evidence is not very strong.

Another limitation of the current consensus is that it provides recommendations based on current knowledge (scientific and clinical expertise) at a specific time point. As such, knowledge in this field will evolve over time, and the management of knee OA may thus evolve and develop accordingly. When interpreting these recommendations, it must be taken into consideration that CBT use is complex and multifactorial. Treatment decision‐making is often not based on isolated factors and involves understanding where in the OA process the clinician meets the patient, which treatments the patient has already had, and includes integration of variable factors, both objective and subjective, including the clinician's personal experience. In this context, it is important to remember the multifactorial nature of the OA knee, where factors such as mechanical malalignment, which could be addressed surgically when relevant, may play a significant role (tibio‐femoral and patello‐femoral malalignment). This presents another limitation of the consensus, which cannot address each and every specific factor and scenario. When considering orthobiologic injections for knee OA, the consensus does not refer to gross mechanical malalignment situations that may require surgical management, although decision‐making should be made on a case‐by‐case basis. An additional limitation is derived from available product variability. Since it would have been impossible to run a consensus process for each available type of CBT product, the consensus was conducted by gathering information and results from several types of products with different characteristics. The consensus findings are therefore applicable to those products for which a good level of clinical evidence is available.

Another limitation, specifically related to the consensus document itself, is that two countries, among those represented by the ESSKA‐affiliated Societies, did not consider the document suitable for adapting to their specific geographic realities. While 90% of the other countries involved expressed satisfaction with the consensus document, it is important to acknowledge that, despite the growing interest in and use of CBT for knee OA, some countries remain more sceptical about the use of CBT and prefer to wait for clearer and more conclusive evidence of treatment efficacy before fully embracing CBT. Interestingly, a recent systematic review highlighted a higher abundance of literature on orthobiologics, particularly blood‐derived products, compared to CS [6, 7], even though CS continues to be used more frequently and with greater certainty. The consensus group recognizes that regulatory and economic aspects may also influence the choice of treatment in some countries. However, it is important to clarify that the consensus document focuses solely on the appropriateness of using CBT in knee OA treatment, and costs were not taken into consideration.

Finally, this consensus does not aim to identify the ideal patient/candidate for CBT treatment but rather to provide recommendations that may aid in addressing commonly encountered scenarios when considering CBT products for knee OA.

## CONCLUSIONS

The consensus acknowledges that there remains some debate about the full extent of CBT's effectiveness. However, the body of evidence has steadily grown with data indicating potential clinical benefits, particularly in pain relief and functional improvement. The consensus results support the use of both POC‐CBT and expanded‐CBT as a viable second‐line injectable treatment option for knee OA, mainly of Grades 1–3, although clinical benefit has also been shown in KL Grade 4.

The consensus underscores the potential of CBT as an alternative to traditional injectable treatments, such as CS and HA, although emphasizing that CBT should generally not be considered as a first‐line injectable treatment option nor a first‐line injectable orthobiologic treatment. The variability in treatment protocols, cell sources, and preparation methods presents challenges for clinical practice, highlighting the urgent need for more high‐level comparative studies and standardized guidelines.

Overall, this consensus document offers a valuable framework to guide clinicians through the complexities of using CBT for knee OA. It provides evidence‐based recommendations while recognizing existing limitations and the need for continued research in this area.

## AUTHOR CONTRIBUTIONS

All authors contributed substantially to the development of this consensus manuscript. The manuscript was drafted by Laura de Girolamo and Lior Laver and critically revised for important intellectual content by Philippe Beaufils and then by all co‐authors. All authors approved the final version of the manuscript and agreed to be accountable for all aspects of the work.

## ESSKA‐ORBIT GROUP

ESSKA‐ORBIT Group is composed of Laura de Girolamo, Lior Laver, Giuseppe Filardo, Ferran Abat, Kristoffer Barfod, Ricardo Bastos, Ramon Cugat, Michael Iosifidis, Baris Kocaoglu, Elizaveta Kon, Jeremy Magalon, Rodica Marinescu, Marko Ostojic, Mikel Sanchez, Thomas Tischer, Diego Delgado, Patricia Laiz and Montserrat García. Scientific advisors: Jasmin Bagge, Konrad Slynarski and Lucienne Vonk.

## CONFLICT OF INTEREST STATEMENT

Laura de Girolamo: Honoraria or Consultation Fees: Lipogems International (IT); Grants/research supports: Fidia (IT); Company speaker honorarium: Arthrex (USA). Elizaveta Kon: Honoraria or Consultation Fees: Cartiheal (IL), Green Bone (IT), Geistlich (CH); Grants/research supports: Zimmer Biomet (USA), Mastelli (IT), Fidia (IT), Cartiheal (IL); Company speaker honorarium: Zimmer Biomet (USA), Fidia (IT); Stock shareholder: Cartiheal (IL). Jeremy Magalon: Honoraria or Consultation Fees: Fidia (IT), Horiba (JP), Macopharma (FR), Arthrex (USA), Horus (FR); Cofounder of Remedex (FR). Lucienne Vonk: Employed by Xintela AB (SW); Stock shareholder: Xintela AB (SW). The remaining authors declare no conflicts of interest.

## ETHICS STATEMENT

The ethics statement is not applicable.

## Supporting information

Supporting File 1_anonymized.

## Data Availability

The data that support the findings of this study are available in Supporting Information S1 of this article, as well as being openly available on the ESSKA website at https://esskaeducation.org/esska-consensus-projects.
