# Supplementary material for: The use of injectable orthobiologics for knee osteoarthritis: A formal ESSKA‐ORBIT consensus. Part 2—Cell‐based therapy
Source: Knee Surg Sports Traumatol Arthrosc. 2025 Sep 9;33(11):4079–95. doi: 10.1002/ksa.70001 (PMC12582236; doi:10.1002/ksa.70001)
Supplement: Supplementary file 1 — Supporting File 1_anonymized. [file KSA-33-4079-s001.docx]

**The use of injectable Orthobiologics for knee osteoarthritis: a formal ESSKA consensus**

**Part 2 – Cell-Based Therapy (CBT)**

**Introduction**

The field of Orthobiologics continues to develop as a result of the growing interest in biologic approaches for the treatment of a variety of musculoskeletal conditions and nowadays it is clear and evident that the use of orthobiologics, both blood-derived and cell-based products, is widespread in most countries around the world. Despite the increasing volume of publications and data, the outcomes of these treatments are still inconclusive because of the lack of unanimous opinion by professionals in terms of patients’ indications, administration protocols and even more in the choice of the available options/devices. Moreover, therapy developers and providers must address hurdles from regulatory issues, through reimbursement considerations and to commercial challenges before successful orthobiologic procedures are available to patients. All of this risks to devalue the potential and the use of these treatments, with a potential loss of valid care opportunities.

In response to this, as Europe’s largest association of musculoskeletal specialists, ESSKA, through the creation of the Orthobiologic Initiative (ORBIT) has highlighted the value of establishing and assemble a pan-European/International collaboration to create a common language and a uniform and responsible voice in the field of orthobiologics as well as driving good standard of care in this field.

**Mission/scope of the ESSKA Orthobiologics Initiative (ORBIT)**

ORBIT focuses on orthobiologic treatment options and strategies for variable musculoskeletal conditions/pathologies. In addition to promote activities to systematically evaluate the effectiveness of existing and emerging orthobiologic treatments, the ORBIT leadership felt the impellent need to provide daily practitioners with consensus documents containing answers to the most common practical questions around the use of orthobiologics, based on the most up-to-date clinical literature and expert opinion, with the final aim to avoid misuse of these therapies.

Since injectable orthobiologic options are the most widely used, ORBIT decided to initiate a formal consensus process in order to address these treatment options, divided into non transfusional hemo-components or blood-derived products (including but not limited to Platelet Rich Plasma, Part 1), and cell-based therapy (sometimes referred to, although improperly, as "stem cell therapy", Part 2).

After the successful completion and release of the Part 1 of the ORBIT consensus project on the use of injectable blood-derived products for the treatment of knee osteoarthritis, available online (https://cdn.ymaws.com/www.esska.org/resource/resmgr/docs/consensus_projects/FINAL_document_ORBIT_long.pdf) and recently published on Knee Surgery Sports Traumatology Arthroscopy (32(4):783-797. doi: 10.1002/ksa.12077), the same group of experts has prepared a second part on the use of injectable cell-based therapy (CBT) products for the same joint condition.

Such an approach is necessary to properly lay the groundwork for their use by clinicians, equipping them to make informed decisions regarding CBT treatment options and allow improved and meaningful patient-informed decision-making.

The Initiative’s main mission is not to develop guidelines but to promote the responsible use of orthobiologics in clinical practice according to the current knowledge and expert opinion, by defining clear indications as well as improved assessment and monitoring recommendations.

**The ESSKA Formal Consensus**

The formal consensus method was first proposed by the High Authority of Health (HAS – Haute Autorité de Santé) in France. It is derived from the Delphi method and it is defined as a way to synthesize information and compare contradictory opinions, with the aim of defining the degree of agreement within a group of selected individuals^1^. Its purpose is to formalize the degree of agreement among experts by identifying and selecting, through iterative ratings with feedback, the points on which experts agree and the points on which they disagree or are undecided. Recommendations are subsequently based on agreement points. The recommendations must be concise, based on the formal agreement of experts and, according to the literature available, with the levels of evidence identified, unambiguous, and clearly respond to the questions raised.

Therefore, the goal of any consensus is not to propose strict guidelines, but to provide recommendations to those who want to offer their patients a given treatment in a supported manner.

In this regard, a consensus document is not devoted to research but to education and it does not start from a hypothesis, but from daily practitioners’ expectations. For this reason, a consensus document aims to provide recommendations and guidance even when the literature is lacking, by exploiting the opinions and experience of the experts who compose the consensus group. Therefore, this consensus document is neither a systematic literature review - or systematic analysis, nor an expert opinion paper, but rather a rigorous and standardized combination of the two, addressing the non-operative management of patients affected by knee osteoarthritis with CBT.

Noteworthy, presenting information on the various specific techniques or commercial systems available was not within the scope of this consensus. Nevertheless, the recommendations regarding Point-of-care CBT (POC-CBT) products are referred only to those obtained by medical devices that have been clinically tested and appropriately studied in the literature.

When considering the use of CBT products for knee OA, one of the main challenges is to identify the ideal patient. Profiling the ideal knee OA patient for CBT products use is complex and multi-factorial. Treatment decision is often not based on isolated factors, and it is the understanding of where in the arthritic process the clinician meets the patient, integrating variable factors, objective and subjective, including the clinician’s personal experience. The scope of this consensus was not to prepare an ‘a-la-carte’ menu to profile the ideal patient/candidate. However, recognizing the need of the above-mentioned document by several practitioners, the ORBIT leadership has initiated another consensus based on the RAND/UCLA Appropriateness Method (RAM). This method aims to produce, through a highly structured approach, patient-focused, contemporary, evidence-based, indications for the use of injectable CBT products for commonly encountered scenarios. The UCLA consensus on the injectable use of CBT will be released shortly.

**Methodology**

The consensus on the injectable use of CBT for knee OA has followed the ESSKA “Formal Consensus Methodology” derived from the modified Delphi methodology^2^.

The core group comprised a Steering Group of 15 experts that was divided into a question and a literature group. The question group proposed a series of relevant questions which were ranked according to clinical importance, answerability and scientific importance by a decision-making software (1000minds.com) that was used for the first time in an ESSKA consensus. The ranked list was then narrowed down and refined by the entire Steering Group.

Following completion of the literature reviews by the Literature Group for each of the questions, the Steering Group produced respective statements based on the existing literature (updated to December 2023) as well as on the entire Steering Group’s expert opinion. Given the great complexity in interpreting current findings on the use of CBT for knee OA and the variability of clinically available devices/methods, in agreement with the consensus project advisors, the steering group felt the need to involve world-class experts (Scientific Advisors) in specific topics addressed in the consensus project.

For each statement, the following grading system was used:

Grade A: high scientific level

Grade B: scientific presumption

Grade C: low scientific level

Grade D: expert opinion

It should be noted that for some statements the degree of recommendation may not perfectly reflect the currently available literature on the subject. This is due to the expert opinion component in the preparation and evaluation of the statements and the interpretation of the literature.

A Rating Group composed by an independent panel of 25 experienced musculoskeletal clinicians (both users and non-users) was asked to review the statements produced by the steering group. The rating phase was composed of two rounds, during which the panel evaluated and ranked each answer according to a discrete numerical scale (Likert scale from 1 to 9, 1 lowest grade of agreement, 9 highest grade of agreement). Appropriateness and agreement were then classified accordingly to the following criteria:

- Appropriate if the median rating was ≥7 and the panel showed consensus (7–9 = strong agreement; 5–9 = relative agreement);
- Uncertain if the median ranged from 4 to 6.5 or consensus was lacking;
- Inapropriate if the median was ≤3.5.

When needed, after the first round the text was modified by the steering group, taking into account the rating group’s comments and a second round to the rating group was carried on. After this, a combined meeting of the steering and rating groups was organized to validate the draft and finalize the following text.

For each statement, in addition to the grade of recommendation, the mean and median rating score is indicated.

In the final step the finalized text was circulated among a Peer Review group to assess the geographic adaptability and acceptance among Europe. The peer review group was set up by the National Societies affiliated to ESSKA, which replied to the call for actions and appointed their representative delegates. The document was peer reviewed by 36 delegates representing 20 ESSKA Affiliated National Societies.

Altogether, the methodology followed during the process, through an iterative process among independent groups, ensures the document’s objectivity and plurality (51 experts between steering, rating group, scientific advisors and other contributors, representing 21 European countries).

**Regulations, cost-effectiveness and geographic adaptability**

During the consensus process, some aspects concerning the regulatory and economic issues around CBT were not taken into consideration given the wide inter-country variability.
In a few European Countries the use of CBT is not authorized by regulatory bodies, neither as minimally manipulated products nor as following an extensive manipulation. In most European countries the use of CBT is permitted under specific rules. One of the still debated issues is the non-homologous use of CBT. The term “homologous use” is used to indicate the repair, reconstruction, replacement, or supplementation of a recipient's cells or tissues with a CBT product that performs the same basic function or functions in the recipient as in the donor. For example, recipient cells or tissues that are identical (e.g., skin for skin) to the donor cells or tissues, and perform one or more of the same basic functions in the recipient as the cells or tissues performed in the donor; or, recipient cells or tissues that may not be identical to the donor's cells or tissues but that perform one or more of the same basic functions in the recipient as the cells or tissues performed in the donor. The use of CBT from bone marrow, adipose tissue, placenta or amniotic membrane to treat knee osteoarthritis would be non-homologous. However, since the regulations are not clearly defined, these therapeutic options are still accepted by the regulatory bodies of most European countries, while requiring to collect data and adopt a monitoring program for these patients.

The use of allogeneic cells also raises several concerns among European regulatory bodies, where most have not approved their routine use yet.

Cost-effectiveness is another issue that concerns the use of CBT, both for products prepared at the point-of-care (POC) and even more so, for extensively manipulated cell products. Although it is clear that the current costs of these procedures are very high and not sustainable for most national health care systems, a thorough health technology assessment would be required before concluding that orthobiologics are not cost-effective in the long term, obviously against a reduction in the costs of these products and procedures.

Because of its nature, a European-level consensus document cannot take into account inter-country variability. However, what this consensus methodology therefore includes is a step to have the consensus document evaluated by representatives from various national societies in the field, nominated by their respective national societies, in order to assess the geographic adaptability of the consensus to their respective healthcare realities. For this reason, all ESSKA affiliated national societies were contacted and asked to nominate expert delegates who could perform this type of evaluation. This last consensus step resulted in an overall acceptance of the documents content (18 societies in favor, 2 against and 2 abstaining due to failure to identify experts).

**ACKNOWLEDGEMENTS**

The Chairs of the ESSKA Consensus Project on Injectable Orthobiologics in Knee OA, Laura de Girolamo and Lior Laver, would like to sincerely thank all the colleagues who contributed to the preparation of this document.

***ESSKA Consensus projects advisor***

Philippe Beaufils (*France*)

***Steering group members***

Abat Ferran (*Spain*)

Barfod Kristoffer (*Denmark*)

Bastos Ricardo (*Portugal*)

Cugat Ramon (*Spain*)

Filardo Giuseppe (*Italy*)

Iosifidis Michael (*Greece*)

Kocaoglu Baris (*Turkey*)

Kon Elizaveta (*Italy*)

Magalon Jeremy (*France*)

Marinescu Rodica (*Romania*)

Ostojic Marko (*Bosnia*-*Erzegovina*)

Sanchez Mikel (*Spain*)

Tischer Thomas (*Germany*)

***Scientific Advisors***

Slynarski Konrad (*Poland*)

Vonk Lucienne (*Sweden*)

Bagge Jasmin (*Denmark*)

***Other contributors (literature search and other relevant tasks)***

Boffa Angelo (*Italy*), Colombini Alessandra (*Italy*), Delgado Diego (*Spain*), Garcia Montserrat (*Spain*), Laiz Patricia (*Spain*), Ragni Enrico (*Italy*), Visconte Caterina (*Italy*), Yossi Sourugeon (*Israel*)

***Rating group members***

Alentorn-Geli Eduard (*Spain*)

Andia Isabel (*Spain*)

Blønd Lars (*Denmark*)

Bøe Berte (*Norway*)

Cengic Tomislav (*Croatia*)

Dallo Ignacio (*Spain*)

Espregueira-Mendes João (*Portugal*)

Heuberer Philippe (*Austria*)

Izadpanah Kaywan (*Germany*)

Kaux Jean-Francois (*Belgium*)

Kovacic Ladislav (*Slovenia*)

Lagae Koen Carl (*Belgium*)

Mangiavini Laura (*Italy*)

Menetrey Jacques (*Switzerland*)

Mogos Stefan (*Romania*)

Papakostas Emmanuel (*Greece*/*Qatar*)

Pengas Yiannis (*UK/Cyprus*)

Pereira Helder (*Portugal*)

Spalding Tim (*UK*)

Piontek Tomasz (*Poland*)

Thoreux Patricia (*France*)

Totlis Trifon (*Greece*)

Ulku Kerem Tekin (*Turkey*)

Yonai Yaniv (*Israel*)

Zaffagnini Stefano (*Italy*)

***ESSKA-affiliated societies for Peer review***

Belgium (BKS), Belarus (BAKAST), Denmark (SAKS), Estonia (EASTS), France (SFTS), Germany-Austria-Switzerland (AGA), Germany (DKG), Greece (HAA), Hungary (MAT), Israel (ISKSA), Italy (SIAGASCOT), Lithuania (LASTA), Luxembourg (LIROMS), Norway (NAA), Poland (PTA), Portugal (SPAT), Romania (SRATS), Spain (SEROD), Sweden (SFAIM), The Netherlands (NVA), Turkey (TUSYAD), United Kingdom (BASK).

***ESSKA Office***

Hansen Anna (Luxembourg/Poland)

The Chairs of the ESSKA Consensus Project on Injectable Orthobiologics

Laura de Girolamo (Italy) Lior Laver (Israel)

Luxembourg, May 2024

**Conflicts of interest of consensus participants:**

*Steering group*

LdG: Honoraria or Consultation Fees: Lipogems International (IT)

Grants/research supports: Fidia (IT)

Company speaker honorarium: Arthrex (USA)

EK: Honoraria Or Consultation Fees: Cartiheal (IL), Green Bone (IT), Geistlich (CH)

Grants/research supports: Zimmer Biomet (USA), Mastelli (IT), Fidia (IT), Cartiheal (IL)

Company speaker honorarium: Zimmer Biomet (USA), Fidia (IT)

Stock shareholder: CARTIHEAL (IL)

JM: Educational support: Fidia (IT), Horiba (JP), Macopharma (FR), Arthrex (USA), Horus (FR)

Co-founder of Remedex (FR)

LV: Employed by Xintela AB (SW); Stock shareholder: Xintela AB (SW)

All other members of the steering group reported no relevant conflicts of interest.

*Rating group*

IA: Scientific advisory board: Spry Bio Inc (SP)

BB: Company speaker honorarium: Smith&Nephew (USA), Ortomedic (NO), Johnson&Johnson (USA)

Grants/research supports: Smith&Nephew (USA)

LB: Honoraria Or Consultation Fees: Arthrex (USA)

Company speaker honorarium: Arthrex (USA)

JM: Royalties: Springer Publisher

Honoraria Or Consultation Fees: Johnson&Johnson Depuy Mitek (USA)

Company speaker honorarium: Johnson&Johnson Depuy Mitek (USA)

Grants/research supports: Johnson&Johnson Depuy Mitek (USA)

TS: Royalties (on products unrelated to the consensus topic): Conmed (USA)

Scientific advisory board: Episurf (SW), Ortonika (UK)

All other members of the rating group reported no relevant conflicts of interest.

**QUESTIONS AND STATEMENTS COLLECTION**

**The use of injectable Orthobiologics for knee osteoarthritis: an ESSKA consensus**

**Part 2 – Cell-based therapy (CBT)**

The questions are divided into 3 (three) sections:

Section 1: CBT Rationale/Indications (Question 1-12)

Section 2: CBT Preparation/Characterization (Question 13-18)

Section 3: CBT Protocol (Question 19-23)

**Abbreviations:**

AT-MSCs Adipose Tissue-derived Mesenchymal Stem Cells

BMA Bone Marrow Aspirate

BMAC Bone Marrow Aspirate Concentrate

BM-MSCs Bone Marrow-derived Mesenchymal Stem Cells

CBT Cell-Based Therapy

CFU-f Colony Forming Unit-Fibroblast

CS Corticosteroids

HA Hyaluronic Acid

hA-MSCs Human Amniotic Mesenchymal Stem Cells

HSC Hematopoietic Stem Cells

IA Intra Articular

IKDC International Knee Documentation Committee
KOOS Knee Injury and Osteoarthritis Outcome Score
KL Kellgren-Lawrence

LEFS Lower Extremity Functional Scale

LP-PRP Leukocyte Poor-Platelet Rich Plasma

LR-PRP Leukocyte Rich- Platelet Rich Plasma

MCID Minimal Clinically Important Difference

MFAT Microfragmented Adipose Tissue

MR Magnetic Resonance

MSCs Mesenchymal Stem Cells

NS Normal Saline

NSAIDS Non Steroidal Anti-Inflammatory Drugs

OA Osteoarthritis

PBMCs Peripheral Blood Mononuclear Cells

POC Point of Care

PRGF Platelet Rich Growth Factors

PRP Platelet Rich Plasma

RCT Randomized Controlled Trial

SF-12 12-Item Short Form Health Survey Questionnaire

SF-36 36-Item Short Form Health Survey Questionnaire

SVF Stromal Vascular Fraction (From Adipose Tissue)

UC-MSCs Umbilical Cord-derived Mesenchymal Stem Cells

VAS Visual Analog Scale (Pain)

WOMAC Western Ontario And McMaster Universities Osteoarthritis Index

WORMS Whole-Organ Magnetic Resonance Imaging Score

**IMPORTANT:**

**All the statements are based on the combination of the experts’ opinions and the current existing literature findings. As such, the recommendations regarding POC-CBT products are referred only to those obtained by medical devices that have been clinically tested and appropriately studied in the literature.**

**SECTION 1**

**QUESTION 1**

**Does current evidence support the use of CBT for knee osteoarthritis (OA)?**

**Statement**

***Point of Care (POC)-CBT***

Current scientific evidence has shown that the use of Point-of-care (POC)-CBT products for knee OA can provide clinical benefit and is a safe treatment option, although certain limitations of current evidence exist due to heterogeneity of products and lack of studies on larger populations. Clinical improvement has been shown at both shorter (6 months) and longer (12 months) durations in most of the studies available in the literature. **The consensus group therefore concludes that there is sufficient clinical evidence to support the use of POC-CBT as a treatment option for knee OA** (see following questions addressing CBT specifications and indications).

However, due to the lack of sufficient high-quality studies in larger populations, as well as the lack of superiority in some studies compared to corticosteroids (CS) or platelet rich plasma (PRP) injections, the full clinical benefit and role of POC-CBT products in the treatment algorithm for knee OA is not completely understood and, as such, **the consensus group currently does not recommend the use of POC-CBT as a first line injectable treatment for knee OA**. The consensus group, therefore, does agree that CBT could be considered when other non-operative and other injectable measures have failed and in circumstances where surgery is not yet indicated or medically appropriate.

Grade of recommendation: B

Rating score: Mean 8.4±0.8; Median 8.5 (6-9) – Appropriate with relative agreement

**(13 RCTs, of which 4 double-blind and 6 single-blind)**

***Expanded-CBT***

Current scientific evidence supports the clinical benefit/efficacy and safety of **Expanded-CBT** for knee OA, confirming the findings of preclinical research. Clinical improvement has been shown at both shorter (6 months) and longer (up to 24 months) durations in most of the published studies. **The consensus group therefore concludes that there is sufficient preclinical and clinical evidence to support the use of Expanded-CBT as a treatment option for knee OA** (see following questions addressing Expanded-CBT specifications and indications) when regulatory approval exists.

Grade of Recommendation: A

Rating Score: Mean 8.2±0.9; Median 8 (6-9) - Appropriate with relative agreement

Due to the complexity of the preparation procedure of autologous Expanded-CBT products, **the consensus group currently does not recommend the use of Expanded-CBT as a first-line injectable treatment for knee OA.** The consensus group agrees that **Expanded-CBT** could be considered when other non-operative and other injectable measures have failed and in circumstances where surgery is not yet indicated or not medically appropriate.

Grade of recommendation: B

Rating Score: Mean 8.3±1.0; Median 8.5 (5-9) Appropriate with relative agreement

**Literature summary**

The current literature presents several studies about the efficacy of intra-articular injections of CBT for treating early to advance knee OA, concerning pain reduction, functional improvement, and quality of life.

Several randomized controlled trials (RCTs) include both cell-based products (one step procedures) prepared at the point of care (POC), such as BMAC and MFAT or SVF, and in vitro expanded MSC (two step procedures).

Only Level I/II SR and MA of RCTs were included and discussed herein.

In general, most of the studies report favorable outcomes of CBT for knee OA, with only few studies showing no significant difference, but never detrimental effects. Among the latter, two Meta Analysis showed that expanded MSC interventions did not result in a relevant reduction of joint pain or improvement of joint function^1,2^. Similarly, in Xu and colleagues report^3^, the comprehensive evaluation index indicated no significant differences after MSC treatment although the assessment of clinical symptoms and cartilage morphology showed improvement. Limited evidence in pain relief and functional improvement was also reported in a level II MA^4^.

On the contrary, a larger number of meta-analyses and systematic reviews as well as RCTs supports the use of CBT for knee OA.

Concerning **expanded mesenchymal stem cells (MSCs)**, a recent systematic review and meta-analysis suggest that MSCs could significantly decrease the visual analog scale in a 12-month follow-up study compared to PRP, hyaluronic acid, and normal saline^5^. Another recent systematic review of high-quality level 1 studies presented better patient-reported outcomes measures at 6 and 12 months for expanded AT-MSCs (adipose tissue-derived mesenchymal stem cells) compared to placebo and hyaluronic acid injections^6^. In a double-blinded RCT, 53 patients with Kellgren–Lawrence grade 1–3 knee OA were recruited to receive intra-articular injection of in vitro expanded MSC from adipose tissue or HA. Significant improvements in WOMAC, VAS, and SF-36 scores were observed in both groups at months 6 and 12 compared with baseline. Compared with the HA group, significantly more patients achieved 50% improvement of WOMAC and a trend of more patients achieved a 70% improvement rate in cell group after 12 months, together with a notably increase in articular cartilage volume in the cell group only after 12 months as measured by MRI^7^. Similarly, another Level I double-blind RCT involving 146 with Kellgren-Lawrence grade 2 and 3 patients showed that an intra-articular injection of allogeneic bone marrow-resulted in significant improvements in the WOMAC total score compared with the placebo group at 6 and 12 months. T2 mapping showed that there was no worsening of deep cartilage in the medial femorotibial compartment of the knee in the cell group at 12-month follow-up, whereas in the placebo group, there was significant and gradual worsening of cartilage^8^.

**Among one step procedures (POC products)** BMA/BMAC injections showed improved clinical outcomes when compared with patients who received hyaluronic acid (HA)^9^. A similar positive outcome was observed for SVF in two meta-analyses (respectively of level I and II), with remarkable clinical efficacy and safety in the short term, comparable with expanded MSC^6,10^. In another meta-analysis, level of evidence II, SVF resulted as a preferable option for pain and functional outcomes when compared to other treatments, including BMA/BMAC, PRP and HA^11^. MSC component resulted as the leading active principle as reported^12^ with expanded cells having significant improvements assessed via KOOS, VAS, WOMAC, and MRI. This is supported by one SR^13^ and two MA^6,10^ where adipose tissue MSCs (AT-MSCs) and SVF ended in significant pain improvement and safety in the short term. Consistently, MSCs, regardless their origin, demonstrated evidence of a beneficial effect of intra-articular injections as reported in several systematic reviews^14–16^ and meta-analyses^17–21^. Of note, all reports confirm positive outcomes for pain and physical function at both short (6 months) and long (12 months) terms, with paucity or inconclusive data for tissue regeneration because of the lack of tools sufficiently sensitive to assess this parameter.

In addition, a recent randomized control trial with long-term follow up (5 years) conducted on 126 patients compared the outcomes after intra-articular injection of either SVF or HA^22^. The Authors concluded that VAS and WOMAC scores in the SVF group were significantly better than in the HA group for all the parameters evaluated. On the other hand, a randomized control trial comparing the efficacy of BMAC and PRP on pain and function in 84 patients with knee OA up to 24 months after injection showed improvements in both groups from baseline to final follow-up, but without inter-group difference^23^.

When comparing sources, AT-MSCs were described as superior to bone marrow MSCs (BM-MSCs) in four MA^24–27^ and to umbilical cord MSCs (UCMSCs) in one MA^28^. Conversely, in one MA, better outcomes were obtained with the use of BM-MSCs as compared with AT-MSCs, and with the use of expanded MSC as opposed to uncultured MSC^29^.

**References**

1. Han SB, Seo IW, Shin YS. Intra-Articular Injections of Hyaluronic Acid or Steroids Associated With Better Outcomes Than Platelet-Rich Plasma, Adipose Mesenchymal Stromal Cells, or Placebo in Knee Osteoarthritis: A Network Meta-analysis. *Arthroscopy*. 2021;37(1):292-306. doi:10.1016/j.arthro.2020.03.041

**Study type: Network Meta-analysis; Level of evidence 2.**

2. Dai W, Leng X, Wang J, et al. Intra-Articular Mesenchymal Stromal Cell Injections Are No Different From Placebo in the Treatment of Knee Osteoarthritis: A Systematic Review and Meta-analysis of Randomized Controlled Trials. *Arthroscopy*. 2021;37(1):340-358. doi:10.1016/j.arthro.2020.10.016

**Study type: Systematic Review Meta-analysis; Level of evidence 1.**

3. Xu S, Liu H, Xie Y, Sang L, Liu J, Chen B. Effect of mesenchymal stromal cells for articular cartilage degeneration treatment: a meta-analysis. *Cytotherapy*. 2015;17(10):1342-1352. doi:10.1016/j.jcyt.2015.05.005

**Study type: Meta-analysis; Level of evidence 2.**

4. Kim SH, Ha CW, Park YB, Nam E, Lee JE, Lee HJ. Intra-articular injection of mesenchymal stem cells for clinical outcomes and cartilage repair in osteoarthritis of the knee: a meta-analysis of randomized controlled trials. *Arch Orthop Trauma Surg*. 2019;139(7):971-980. doi:10.1007/s00402-019-03140-8

**Study type: Meta-analysis; Level of evidence 2**

5. Song Y, Zhang J, Xu H, et al. Mesenchymal stem cells in knee osteoarthritis treatment: A systematic review and meta-analysis. *J Orthop Translat*. 2020;24:121-130. doi:10.1016/j.jot.2020.03.015

**Study type: Systematic Review and Meta-analysis; Level of evidence 4.**

6. Kim KI, Kim MS, Kim JH. Intra-articular Injection of Autologous Adipose-Derived Stem Cells or Stromal Vascular Fractions: Are They Effective for Patients With Knee Osteoarthritis? A Systematic Review With Meta-analysis of Randomized Controlled Trials. *Am J Sports Med*. 2023;51(3):837-848. doi:10.1177/03635465211053893

**Study type: Meta-analysis; Level of evidence 1.**

7. Lu L, Dai C, Zhang Z, et al. Treatment of knee osteoarthritis with intra-articular injection of autologous adipose-derived mesenchymal progenitor cells: a prospective, randomized, double-blind, active-controlled, phase IIb clinical trial. *Stem Cell Res Ther*. 2019;10(1):143. doi:10.1186/s13287-019-1248-3

**Study type: RCT; Level of evidence 1.**

8. Gupta PK, Maheshwari S, Cherian JJ, et al. Efficacy and Safety of Stempeucel in Osteoarthritis of the Knee: A Phase 3 Randomized, Double-Blind, Multicenter, Placebo-Controlled Study. *Am J Sports Med*. 2023;51(9):2254-2266. doi:10.1177/03635465231180323

**Study type: RCT; Level of evidence 1.**

9. Belk JW, Lim JJ, Keeter C, et al. Patients With Knee Osteoarthritis Who Receive Platelet-Rich Plasma or Bone Marrow Aspirate Concentrate Injections Have Better Outcomes Than Patients Who Receive Hyaluronic Acid: Systematic Review and Meta-analysis. *Arthroscopy*. 2023;39(7):1714-1734. doi:10.1016/j.arthro.2023.03.001

**Study type: Systematic Review and Meta-analysis; Level of evidence 1.**

10. Yang Y, Lan Z, Yan J, et al. Effect of intra-knee injection of autologous adipose stem cells or mesenchymal vascular components on short-term outcomes in patients with knee osteoarthritis: an updated meta-analysis of randomized controlled trials. *Arthritis Res Ther*. 2023;25(1):147. doi:10.1186/s13075-023-03134-3

**Study type: Meta-analysis; Level of evidence 2.**

11. Anil U, Markus DH, Hurley ET, et al. The efficacy of intra-articular injections in the treatment of knee osteoarthritis: A network meta-analysis of randomized controlled trials. *Knee*. 2021;32:173-182. doi:10.1016/j.knee.2021.08.008

**Study type: Network meta-analysis; Level of evidence 2.**

12. Ding W, Xu YQ, Zhang Y, et al. Efficacy and Safety of Intra-Articular Cell-Based Therapy for Osteoarthritis: Systematic Review and Network Meta-Analysis. *Cartilage*. 2021;13(1_suppl):104S-115S. doi:10.1177/1947603520942947

**Study type: Systematic Review and Network Meta-analysis; Level of evidence 1.**

13. Biazzo A, D’Ambrosi R, Masia F, Izzo V, Verde F. Autologous adipose stem cell therapy for knee osteoarthritis: where are we now? *Phys Sportsmed*. 2020;48(4):392-399. doi:10.1080/00913847.2020.1758001

**Study type: Systematic Review; Level of evidence 2.**

14. Wiggers TG, Winters M, Van den Boom NA, Haisma HJ, Moen MH. Autologous stem cell therapy in knee osteoarthritis: a systematic review of randomised controlled trials. *Br J Sports Med*. 2021;55(20):1161-1169. doi:10.1136/bjsports-2020-103671

**Study type: Systematic Review; Level of evidence 2.**

15. Hall M, McCafferty J, Agarwalla A, Nwachuku I, Liu JN, Amin NH. Safety and Efficacy of Cultured/Noncultured Mesenchymal Stromal Cells without Concurrent Surgery for Knee Osteoarthritis: A Systematic Review of Randomized Controlled Trials. *J Long Term Eff Med Implants*. 2020;30(1):31-47. doi:10.1615/JLongTermEffMedImplants.2020035281

**Study type: Systematic Review; Level of evidence 2.**

16. Gong J, Fairley J, Cicuttini FM, et al. Effect of Stem Cell Injections on Osteoarthritis-related Structural Outcomes: A Systematic Review. *J Rheumatol*. 2021;48(4):585-597. doi:10.3899/jrheum.200021

**Study type: Systematic Review; Level of evidence 2.**

17. Qu H, Sun S. Efficacy of mesenchymal stromal cells for the treatment of knee osteoarthritis: a meta-analysis of randomized controlled trials. *J Orthop Surg Res*. 2021;16(1):11. doi:10.1186/s13018-020-02128-0

**Study type: Meta-analysis; Level of evidence 2.**

18. Naja M, Fernandez De Grado G, Favreau H, et al. Comparative effectiveness of nonsurgical interventions in the treatment of patients with knee osteoarthritis: A PRISMA-compliant systematic review and network meta-analysis. *Medicine (Baltimore)*. 2021;100(49):e28067. doi:10.1097/MD.0000000000028067

**Study type: Systematic review and Network Meta-analysis; Level of evidence 1.**

19. Yubo M, Yanyan L, Li L, Tao S, Bo L, Lin C. Clinical efficacy and safety of mesenchymal stem cell transplantation for osteoarthritis treatment: A meta-analysis. *PLoS One*. 2017;12(4):e0175449. doi:10.1371/journal.pone.0175449

**Study type: Meta-analysis; Level of evidence 2.**

20. Kim SH, Djaja YP, Park YB, Park JG, Ko YB, Ha CW. Intra-articular Injection of Culture-Expanded Mesenchymal Stem Cells Without Adjuvant Surgery in Knee Osteoarthritis: A Systematic Review and Meta-analysis. *Am J Sports Med*. 2020;48(11):2839-2849. doi:10.1177/0363546519892278

**Study type: Systematic Review and meta-analysis; Level of evidence 2.**

21. Ma W, Liu C, Wang S, Xu H, Sun H, Fan X. Efficacy and safety of intra-articular injection of mesenchymal stem cells in the treatment of knee osteoarthritis: A systematic review and meta-analysis. *Medicine (Baltimore)*. 2020;99(49):e23343. doi:10.1097/MD.0000000000023343

**Study type: Systematic review and Meta-analysis; Level of evidence 2.**

22. Zhang S, Xu H, He B, et al. Mid-term prognosis of the stromal vascular fraction for knee osteoarthritis: a minimum 5-year follow-up study. *Stem Cell Res Ther*. 2022;13(1):105. doi:10.1186/s13287-022-02788-1

**Study type: RCT; Level of evidence 1.**

23. Anz AW, Plummer HA, Cohen A, Everts PA, Andrews JR, Hackel JG. Bone Marrow Aspirate Concentrate Is Equivalent to Platelet-Rich Plasma for the Treatment of Knee Osteoarthritis at 2 Years: A Prospective Randomized Trial. *Am J Sports Med*. 2022;50(3):618-629. doi:10.1177/03635465211072554

**Study type: RCT; Level of evidence 2.**

24. Zhao D, Pan JK, Yang WY, et al. Intra-Articular Injections of Platelet-Rich Plasma, Adipose Mesenchymal Stem Cells, and Bone Marrow Mesenchymal Stem Cells Associated With Better Outcomes Than Hyaluronic Acid and Saline in Knee Osteoarthritis: A Systematic Review and Network Meta-analysis. *Arthroscopy*. 2021;37(7):2298-2314.e10. doi:10.1016/j.arthro.2021.02.045

**Study type: Systematic Review and Network Meta-analysis; Level of evidence 2.**

25. Jeyaraman M, Muthu S, Ganie PA. Does the Source of Mesenchymal Stem Cell Have an Effect in the Management of Osteoarthritis of the Knee? Meta-Analysis of Randomized Controlled Trials. *Cartilage*. 2021;13(1_suppl):1532S-1547S. doi:10.1177/1947603520951623

**Study type: Meta-analysis; Level of evidence 2.**

26. Zhang Y, Yang H, He F, Zhu X. Intra-articular injection choice for osteoarthritis: making sense of cell source-an updated systematic review and dual network meta-analysis. *Arthritis Res Ther*. 2022;24(1):260. doi:10.1186/s13075-022-02953-0

**Study type: Systematic Review and Network Meta-Analysis; Level of evidence 2.**

27. Han X, Yang B, Zou F, Sun J. Clinical therapeutic efficacy of mesenchymal stem cells derived from adipose or bone marrow for knee osteoarthritis: a meta-analysis of randomized controlled trials. *J Comp Eff Res*. 2020;9(5):361-374. doi:10.2217/cer-2019-0187

**Study type: Meta-analysis; Level of evidence 2.**

28. Wei ZJ, Wang QQ, Cui ZG, Inadera H, Jiang X, Wu CA. Which is the most effective one in knee osteoarthritis treatment from mesenchymal stem cells obtained from different sources? A systematic review with conventional and network meta-analyses of randomized controlled trials. *Ann Transl Med*. 2021;9(6):452. doi:10.21037/atm-20-5116

**Study type: Systematic Review and Network Meta-Analysis; Level of evidence 2.**

29. Tan SHS, Kwan YT, Neo WJ, et al. Intra-articular Injections of Mesenchymal Stem Cells Without Adjuvant Therapies for Knee Osteoarthritis: A Systematic Review and Meta-analysis. *Am J Sports Med*. 2021;49(11):3113-3124. doi:10.1177/0363546520981704

**Study type: Systematic Review and Meta-analysis; Level of evidence 2.**

**QUESTION 2**

**Statement**

**For which degrees of knee OA is CBT recommended?**

Current evidence has shown the clinical benefit of CBT in knee OA Kellgren-Lawrence (KL) grades 1-4, however most studies involved populations with KL grades 2-3.

**The consensus group recommends CBT can be used for knee OA mainly in grades 1-3, although clinical benefit have also been shown in KL grade 4.**

Grade of recommendation: B

Rating Score: Mean 8.1±1.0; Median 8 (6-9) Appropriate with relative agreement

*This statement is valid for both POC- and Expanded-CBT*

**Literature summary (See Literature Summary of Question 3)**

**QUESTION 3**

**Can CBT be used in severe knee OA (Kellgren-Lawrence 4)?**

**Statement**

In comparison to Kellgren-Lawrence **(**KL) grades 1-3, although roughly half of the studies include patients with KL 4, the evidence on CBT use for KL 4 is more scarce given the lower number of patients presenting with this advanced OA grade. Nevertheless, the evidence does show that CBT can also be used in severe knee OA (KL 4), with clinical benefits lasting up to 12 months. **Therefore, the consensus group suggests that CBT could be considered as a treatment option in severe knee OA (KL 4),** especially in individuals who have failed other non-operative strategies (including other injectable therapies) and who are not yet indicated for or are not willing to undergo knee replacement surgery or cannot undergo such surgery due to comorbidities. **However, the consensus group also recommends informing such patients that outcomes may not be as favorable or may not last as long as for lower grades of knee OA.**

Grade of recommendation: C

Rating Score: Mean 8.2±0.8; Median 8 (7-9) Appropriate with strong agreement

*This statement is valid for both POC- and Expanded-CBT*

**Literature summary** **(Question 2 & 3)**

Several studies have evaluated the injectable use of cell-based therapy in different stages of knee osteoarthritis based on Kellgreen-Lawrence (KL) classification. In particular, analyzing 38 level I and II clinical studies reporting data about CBT injections, 33% and 50% of the studies for POC products or expanded cells, respectively, included also patients with end-stage KOA stage (KL4). However, most of the times the patients with severe knee OA represent only a small percentage of the study cohort.

The vast majority of RCTs reported significant improvements in patients with low to moderate OA grade after the treatment with CBT, with advantages over control, however often with an unclear association with the severity of knee OA^1–5^, in some cases up to 5 years follow-up.

Other Level I and II studies offered a deeper analysis in terms of OA severity and clinical benefit of CBT. Dilogo et al.^6^ compared two groups of patients based on K-L classification (K-L 1-2, n=33; K-L 3-4, n=22) treated with expanded umbilical cord MSCs. Significant improvement in VAS, IKDC and WOMAC were observed in both groups with maximum effect at 6 months, with no difference between groups. On the other side, Tran et al.^7^ compared patients with KL 2 and 3 treated with a single injection of stromal vascular fraction (SVF) at 24 months. They concluded that stromal vascular fraction (SVF) therapy is effective in the recovery of both groups of patients but with better results in KL 3 than in KL 2 grade. Conversely, Pintore et al.^8^ in a prospective comparative clinical trial using either nanofragmented adipose tissue bone marrow aspirate concentrate to treat patients with KL grade 2-4, showed better functional and clinical outcomes in patients with KL 2 than patients with KL grades 3 and 4.

Around 20 level I and II studies report the results of CBT in the treatment of patients with severe knee OA (KL4). They all showed satisfactory results meant as improvement over baseline and/or compared to control groups. Vega A et al.^9^ in a RCT, evaluated patients with K-L 2-4 and observed that allogeneic MSC therapy was superior to HA. Chahal et al.^10^ showed that the use of BM-MSC was safe and results in improvement in PROMs in patients with late-stage knee OA 12 months after a single MSC injection. Higuchi J et al.^11^ in a clinical trial, showed that in patients with K-L 3-4, an intra-articular administration of autologous AT-MSCs had improvement of VAS and KOOS-sports/recreation and was significantly higher in patients with more severe cartilage lesions.

Heidari N et al.^12^, in a prospective, gender-bias mitigated, reproducible analysis at two years evaluated patients K-L 3 and 4 who underwent MFAT injection. The treatment was able to improve quality of life in patients with KOA who were deemed suitable for knee replacement. Pers YM et al.^13^ in a non-randomized dose escalation clinical trial, showed that intra-articular injection of AT-MSCs was a safe therapeutic alternative to treat severe knee OA patients (K-L 3 and 4). However, only a few of them reported separated data focusing on only KL4 patients. In their study Zaffagnini et al.^14^ showed that, despite a general lack of superiority of MFAT over PRP, as a secondary outcome, more patients in the MFAT group with severe OA reached the minimal clinically important difference for the IKDC score at 6 months compared with the PRP group.

These findings as well as others are summarized in systematic reviews and meta-analyses. Wiggers et al.^15^ in a systematic review reported the results of 14 RCTs for a total of 408 patients analyzed, of which the majority with K-L grade 2 (33%) and 3 (50%) KOA. They found a positive effect of autologous CBT on clinical (28/43; 65%) and radiological (MRI) outcome measures (5/6; 83%), with an improvement 1 year after the treatment in 19/26 (73%) cases, regardless of KL grade. However, the risk of bias was considered high in all the studies. Similarly, in another systematic review Álvarez Hernández^16^ observed clinical and structural benefits in patients with K-L grades 2-4 using expanded MSCs implants in osteoarthritis patients, with no difference among different OA grades.

Ip et al.^17^ in a literature review included 12 RCT focusing on CBT for the treatment of knee OA concluded that MSCs treatment works better than control groups (placebo or HA). In particular, better results were observed in KL grade 2-3 patients compared to patients with severe KOA (KL 4). This was confirmed by a meta-analysis of Cui et al.^18^ that showed that patients submitted to stem cell therapy with lower degrees of K-L grade achieved improved outcomes.

Conversely, the systematic review of Anzillotti et al.^19^ did not demonstrate uniform beneficial effects for the use of injectable orthobiologics and concluded that this would prevent any advice for routine application in the treatment of severe knee OA (K-L 4).

Out of the choir, a network meta-analysis by Han et al.^20^ included 43 non-homogenous RCTs focusing on the clinical effects of hyaluronic acid (HA), steroids, platelet-rich plasma (PRP), or adipose mesenchymal stromal cell (MSC) injections in the treatment of knee osteoarthritis in patients KL grade 1-4. Single PRP, multiple PRP, and adipose MSC interventions did not result in a relevant reduction of joint pain nor improvement of joint function compared with placebo.

**References (Question 2 & 3)**

1. Zhang S, Xu H, He B, et al. Mid-term prognosis of the stromal vascular fraction for knee osteoarthritis: a minimum 5-year follow-up study. *Stem Cell Res Ther*. 2022;13(1):105. doi:10.1186/s13287-022-02788-1

**Study type: RCT; Level of evidence 1.**

2. Hong Z, Chen J, Zhang S, et al. Intra-articular injection of autologous adipose-derived stromal vascular fractions for knee osteoarthritis: a double-blind randomized self-controlled trial. *Int Orthop*. 2019;43(5):1123-1134. doi:10.1007/s00264-018-4099-0

**Study type: RCT; Level of evidence 1.**

3. Centeno C, Sheinkop M, Dodson E, et al. A specific protocol of autologous bone marrow concentrate and platelet products versus exercise therapy for symptomatic knee osteoarthritis: a randomized controlled trial with 2 year follow-up. *J Transl Med*. 2018;16(1):355. doi:10.1186/s12967-018-1736-8

**Study type: RCT; Level of evidence 1.**

4. Kim KI, Lee MC, Lee JH, et al. Clinical efficacy and safety of the intra-articular injection of autologous adipose-derived mesenchymal stem cells for knee osteoarthritis: a phase III, randomized, double-blind, placebo-controlled trial. *Am J Sports Med*. 2023;51(9):2243-2253. doi:10.1177/03635465231179223

**Study type: RCT; Level of evidence 1.**

5. Emadedin M, Labibzadeh N, Liastani MG, et al. Intra-articular implantation of autologous bone marrow-derived mesenchymal stromal cells to treat knee osteoarthritis: a randomized, triple-blind, placebo-controlled phase 1/2 clinical trial. *Cytotherapy*. 2018;20(10):1238-1246. doi:10.1016/j.jcyt.2018.08.005

**Study type: RCT; Level of evidence 1.**

6. Dilogo IH, Canintika AF, Hanitya AL, Pawitan JA, Liem IK, Pandelaki J. Umbilical cord-derived mesenchymal stem cells for treating osteoarthritis of the knee: a single-arm, open-label study. *Eur J Orthop Surg Traumatol*. 2020;30(5):799-807. doi:10.1007/s00590-020-02630-5

**Study type: Uncontrolled Clinical trial; Level of evidence 3.**

7. Tran TDX, Wu CM, Dubey NK, et al. Time- and Kellgren-Lawrence grade-dependent changes in intra-articularly transplanted stromal vascular fraction in osteoarthritic patients. *Cells*. 2019;8(4):308. doi:10.3390/cells8040308

**Study type: Uncontrolled Clinical trial; Level of evidence 3.**

8. Pintore A, Notarfrancesco D, Zara A, et al. Intra-articular injection of bone marrow aspirate concentrate (BMAC) or adipose-derived stem cells (ADSCs) for knee osteoarthritis: a prospective comparative clinical trial. *J Orthop Surg Res*. 2023;18(1):350. doi:10.1186/s13018-023-03841-2

**Study type: Non-randomized clinical trial; Level of evidence 2.**

9. Vega A, Martín-Ferrero MA, Del Canto F, et al. Treatment of knee osteoarthritis with allogeneic bone marrow mesenchymal stem cells: a randomized controlled trial. *Transplantation*. 2015;99(8):1681-1690. doi:10.1097/TP.0000000000000678

**Study type: RCT; Level of evidence 2.**

10. Chahal J, Gómez-Aristizábal A, Shestopaloff K, et al. Bone marrow mesenchymal stromal cell treatment in patients with osteoarthritis results in overall improvement in pain and symptoms and reduces synovial inflammation. *Stem Cells Transl Med*. 2019;8(8):746-757. doi:10.1002/sctm.18-0183

**Study Type: Uncontrolled Clinical Trial; Level of evidence 3.**

11. Higuchi J, Yamagami R, Matsumoto T, et al. Associations of clinical outcomes and MRI findings in intra-articular administration of autologous adipose-derived stem cells for knee osteoarthritis. *Regen Ther*. 2020;14:332-340. doi:10.1016/j.reth.2020.04.003

**Study type: Uncontrolled Clinical trial; Level of evidence 3.**

12. Heidari N, Borg TM, Olgiati S, et al. Microfragmented adipose tissue injection (MFAT) may be a solution to the rationing of total knee replacement: a prospective, gender-bias mitigated, reproducible analysis at two years. *Stem Cells Int*. 2021;2021:9921015. doi:10.1155/2021/9921015

**Study Type: Uncontrolled clinical trial; Level of evidence 3.**

13. Pers YM, Rackwitz L, Ferreira R, et al. Adipose mesenchymal stromal cell-based therapy for severe osteoarthritis of the knee: a phase I dose-escalation trial. *Stem Cells Transl Med*. 2016;5(7):847-856. doi:10.5966/sctm.2015-0245

**Study type: Uncontrolled clinical trial; Level of evidence 3.**

14. Zaffagnini S, Andriolo L, Boffa A, et al. Microfragmented Adipose Tissue Versus Platelet-Rich Plasma for the Treatment of Knee Osteoarthritis: Response. *Am J Sports Med*. 2022; 50(11):2881-2892. doi 10.1177/03635465221115821

**Study type: RCT; Level of evidence 1.**

15. Wiggers TG, Winters M, Van den Boom NA, Haisma HJ, Moen MH. Autologous stem cell therapy in knee osteoarthritis: a systematic review of randomised controlled trials. *Br J Sports Med*. 2021;55(20):1161-1169. doi:10.1136/bjsports-2020-103671

**Study type: Systematic Review; Level of evidence 2.**

16. Álvarez Hernández P, de la Mata Llord J. Expanded Mesenchymal Stromal Cells in knee osteoarthritis: A systematic literature review. *Reumatol Clin (Engl Ed)*. 2022;18(1):49-55. doi:10.1016/j.reumae.2020.10.001

**Study type: Systematic Review; Level of evidence 3.**

17. Ip HL, Nath DK, Sawleh SH, Kabir MH, Jahan N. Regenerative medicine for knee osteoarthritis - the efficacy and safety of intra-articular platelet-rich plasma and mesenchymal stem cells injections: a literature review. *Cureus*. 2020;12(9):e10575. doi:10.7759/cureus.10575

**Study type: Narrative Review; Level of evidence 4.**

18. Cui GH, Wang YY, Li CJ, Shi CH, Wang WS. Efficacy of mesenchymal stem cells in treating patients with osteoarthritis of the knee: A meta-analysis. *Exp Ther Med*. 2016;12(5):3390-3400. doi:10.3892/etm.2016.3791

**Study type: Meta-analysis; Level of evidence 3.**

19. Anzillotti G, Conte P, Di Matteo B, Bertolino EM, Marcacci M, Kon E. Injection of biologic agents for treating severe knee osteoarthritis: is there a chance for a good outcome? A systematic review of clinical evidence. *Eur Rev Med Pharmacol Sci*. 2022;26(15):5447-5459. doi:10.26355/eurrev_202208_29413

**Study type: Systematic Review; Level of evidence 4.**

20. Han SB, Seo IW, Shin YS. Intra-articular injections of hyaluronic acid or steroids associated with better outcomes than platelet-rich plasma, adipose mesenchymal stromal cells, or placebo in knee osteoarthritis: A Network Meta-analysis. *Arthroscopy*. 2021;37(1):292-306. doi:10.1016/j.arthro.2020.03.041

**Study type: Network Meta-analysis; Level of evidence 2.**

**QUESTION 4**

**Is there an age limitation for the use of CBT for knee OA?**

**Statement**

There is insufficient evidence in the literature regarding an age limitation for CBT use in knee OA. The majority of available studies on CBT use for knee OA includes patients between 18 and 75 years of age. However, several in vitro studies have shown/suggested a decline in stem cell quantity and quality with increasing donor age, where adipose tissue-derived mesenchymal stem/stromal cells (MSCs) seem to be less affected by donor age compared to bone marrow-MSCs.

**Current evidence does not indicate clear limitations or associations with regards to cell quantity/cell viability and product efficacy. Therefore, the consensus group agrees that a specific age range cannot be recommended.** In light of the limited evidence on the age factor, the consensus group suggests that treatment decisions should not be based only on chronologic age as other factors should come into consideration**, though it recognizes that there is evidence suggesting a reduced cell quantity and possibly quality with age**.

Grade of recommendation: D

Rating Score: Mean 8.2±1.0; Median 9 (6-9) Appropriate with relative agreement

*This statement is valid for both POC- and Expanded-CBT*

**Literature summary**

The literature is sparse regarding the possible effect of age on CBT therapy outcomes. Although OA is typically a condition associated with aging, an increasing number of patients face this condition, at least in the early stages, well before they reach old age. Although intuitively biological material from younger patients/donors should perform better than that from older patients, just as the reparative/regenerative capacity of a younger body should be superior to that of an older body, the effect of age on CBT performance does not seem to be confirmed by the current literature.

Given the lack of specific literature, the literature search found that most level I and II clinical trials include patients with a wide age range, specifically 18 to 75 years old, with a mean age of approximately 57 years/old. Interestingly no difference has been found between studies on POC products and cultured expanded cells available in the literature in terms of mean age of the patients treated.

Quality assays regarding release criteria typical of culture expanded cell-based products have highlighted that the quality/efficacy of expanded cells from older donors are inferior to younger ones. As a consequence, older donors are excluded to avoid the production of a final preparation that does not meet the release criteria. For POC these quality assays are usually not performed, but also these products might be less effective from older donors.

Given the lack of clinical literature on the effect of age on injectable CBT products, the literature search was addressed to find relevant in vitro studies on patients’ biological material in the search of important evidence that is missing in clinical literature.

*In vitro studies on patients’ material*

A study by Choudhery et al.^1^ found that AT-MSCs from young donors (<40 years) had a greater cellular viability (93.3% ± 2.8%) compared to older donors (>50 years) (84.7% ± 1.2%) after hypoxic insults simulating an ischemic (knee OA) environment.

Cavallo et al.^2^ compared the number and properties of bone marrow stromal cells collected from BMAC obtained from different harvest sites and from patients of different ages. Twenty patients with mild knee OA were included in the study (n= 10 per age group). It was concluded that BMAC of younger patients (19 ± 2.7 years) had a 3 times higher number of mononucleated cells compared to older patients (56.8 ± 12.5 years). A decline in stem cell quantity with increasing age is supported in multiple studies as described by Stolzing et al.^3^, where an age-related decline in MSC quantity commonly is reported around 50 years of age. Common to most of these studies, the age range is wide and range from adolescent to geriatric donors. Few studies with a narrower age-inclusion range found no difference in MSC quantity as a function of age^3^.

A lower harvest quantity is associated with more in vitro cell-doublings required to obtain a certain cell number for treatment with expanded cells compared to a higher harvest quantity. This may lead to cellular senescence, which is reported in various types of MSCs from aged donors, whereby the cells cannot be further expanded and lose their differentiation capacity in addition to secreting a harmful senescence-associated secretory profile^1,3–9^. Studies by Ragni et al.^10^ and Bagge et al.^11^ reported low level of cellular senescence (≤5%) in MFAT from knee OA patients with a mean age of 44 years ± 6, and 52.6 years ± 8.1 (41 to 63 years), respectively.

Human MSCs have been shown to change their morphology upon long-term passage with increasing donor age accelerating the morphological changes^12^.The chondrogenic and osteogenic differentiation performance is shown to decline with increasing age^1,3,4,6,7^. An “adipogenic switch” has been reported where BM-MSCs from aged donors are more prone to adipogenic differentiation compared to osteogenic differentiation^13^. Adipose tissue-derived stem cells are reported to be less affected by donor age compared to BM-MSCs^4,7^. In a review by Zupan et al.^9^, it is further described how age-related MSC exhaustion leads to decreased immunomodulation and decreased paracrine effects. Extrinsic age factors may impact (young) allogeneic cells if implanted into aged OA patients^9^, but more research is needed in this area. On the other hand, transplantation of allogeneic MSCs from young donors have been shown to slow loss of bone density in aged mice^14^.

*Clinical studies combining CBT and surgery*

Regarding clinical literature, given the lack of studies studying the influence of age on intra-articular injections of CBT only, some interesting studies combining intra-articular injections with minor arthroscopic procedures have been included herein in the search of relevant issue related to age and CBT performances. Note that, because of the combination with arthroscopy the age-effect on CBT may thus be affected by other factors.

Wei et al.^15^ reviewed the literature focusing on the effects of doses of different sources of MSC – BMA/BMAC, BM-MSCs, UC-MSCs - for intra-articular MSC injection for knee OA. They concluded that young patients and those with early knee OA are more likely to benefit from intra-articular injection of MSCs, with the age-recommendation primarily based on a study by Koh et al. (2012)^16^. They performed a prospective case-control study where 25 knee OA patients (age 54.2 ± 9.3 years, range 34-69) were treated with MSCs derived from the infrapatellar fat-pad together with PRP and arthroscopic debridement. The authors found that patients >55 years had an increased VAS score and decreased Tegner activity scale score compared to younger patients at 12 months follow-up. In the review by Wei et al.^15^, no specific age cut-off was described, and the origin of cells was not commented on.

Ferracini et al.^17^ performed a prospective Cohort Study Level II multicentric trial involving 91 patients with the diagnosis of early/mild osteoarthritis, and failure of previous conservative measures. They were enrolled to undergo diagnostic arthroscopy (intra articular pathology was treated) and a single microfragmented adipose tissue (MFAT) injection. They concluded that age and synovitis were significant factors influencing the clinical outcome. Better clinical results were observed in patients younger than 60 years without active inflammatory joint processes, leading to consider that age is a parameter that should be considered and that surgery on knee OA patients over 60 years may correlate with negative results.

Vasso and al.^18^ published a study aimed to report the clinical and functional results of a series of patients with isolated primary patellofemoral osteoarthritis (PFOA) treated with intraarticular injection of MFAT plus knee arthroscopy. Twenty-three patients with early-to-moderate PFOA who received this treatment were retrospectively analyzed. The results were also analyzed in relation to age and body mass index (BMI) of patients, and to the stage of PFOA. Differences in improvements of IKS and VAS scores in relation to age (< 60 versus ≥ 60 years), BMI (< 30 versus ≥ 30 kg/m2), and stage of PFOA (stages 1–2 versus stage 3) were finally analyzed. The specific age demographics of the two age-groups were not mentioned, and the general age distribution of (mean ± SD (range)) 58 ± 8 years (45-78) was very close to the age-cut between the two groups, which makes the strength of the data quite low. The authors, however, concluded no significant differences in improvements of IKS knee and function and VAS scores in relation to age, BMI, or stage of PFOA.

An observational, intention to treat study by Borg et al.^19^ reported that women showed a greater improvement in pain and function to MFAT treatment than men, and that age factors played a role in differences between the sexes. The authors did, however, not elaborate further on the age effect, whereby the specific effect of age is unclear to the reader.

**References**

1. Choudhery MS, Badowski M, Muise A, Pierce J, Harris DT. Donor age negatively impacts adipose tissue-derived mesenchymal stem cell expansion and differentiation. *J Transl Med*. 2014;12:8. doi:10.1186/1479-5876-12-8

**Study type: *In vitro* study**

2. Cavallo C, Boffa A, de Girolamo L, et al. Bone marrow aspirate concentrate quality is affected by age and harvest site. *Knee Surg Sports Traumatol Arthrosc*. 2023;31(6):2140-2151. doi:10.1007/s00167-022-07153-6

**Study type: *In vitro* study**

3. Stolzing A, Jones E, McGonagle D, Scutt A. Age-related changes in human bone marrow-derived mesenchymal stem cells: consequences for cell therapies. *Mech Ageing Dev*. 2008;129(3):163-173. doi:10.1016/j.mad.2007.12.002

**Study type: *In vitro* study.**

4. Bagge J, Berg LC, Janes J, MacLeod JN. Donor age effects on in vitro chondrogenic and osteogenic differentiation performance of equine bone marrow- and adipose tissue-derived mesenchymal stromal cells. *BMC Vet Res*. 2022;18(1):388. doi:10.1186/s12917-022-03475-2

**Study type: *In vitro* study.**

5. Bagge J, MacLeod JN, Berg LC. Cellular Proliferation of Equine Bone Marrow- and Adipose Tissue-Derived Mesenchymal Stem Cells Decline With Increasing Donor Age. *Front Vet Sci*. 2020;7:602403. doi:10.3389/fvets.2020.602403

**Study type: *In vitro* study.**

6. Zhou S, Greenberger JS, Epperly MW, et al. Age-related intrinsic changes in human bone-marrow-derived mesenchymal stem cells and their differentiation to osteoblasts. *Aging Cell*. 2008;7(3):335-343. doi:10.1111/j.1474-9726.2008.00377.x

**Study type: *In vitro* study**

7. Chen HT, Lee MJ, Chen CH, et al. Proliferation and differentiation potential of human adipose-derived mesenchymal stem cells isolated from elderly patients with osteoporotic fractures. *J Cell Mol Med*. 2012;16(3):582-593. doi:10.1111/j.1582-4934.2011.01335.x

**Study type: *In vitro* study**

8. Stenderup K, Justesen J, Clausen C, Kassem M. Aging is associated with decreased maximal life span and accelerated senescence of bone marrow stromal cells. *Bone*. 2003;33(6):919-926. doi:10.1016/j.bone.2003.07.005

**Study type: *In vitro* study**

9. Zupan J, Strazar K, Kocijan R, Nau T, Grillari J, Marolt Presen D. Age-related alterations and senescence of mesenchymal stromal cells: Implications for regenerative treatments of bones and joints. *Mech Ageing Dev*. 2021;198:111539. doi:10.1016/j.mad.2021.111539

**Study type: Narrative Review; Level of evidence 4**

10. Ragni E, Viganò M, Torretta E, et al. Characterization of Microfragmented Adipose Tissue Architecture, Mesenchymal Stromal Cell Content and Release of Paracrine Mediators. *J Clin Med*. 2022;11(8):2231. doi:10.3390/jcm11082231

**Study type: *In vitro* study**

11. Bagge J, Hölmich P, Hammer FA, et al. Successful isolation of viable stem cells from cryopreserved microfragmented human adipose tissue from patients with knee osteoarthritis - a comparative study of isolation by tissue explant culture and enzymatic digestion. *J Exp Orthop*. 2023;10(1):31. doi:10.1186/s40634-023-00596-x

**Study type: *In vitro* study**

12. Zaim M, Karaman S, Cetin G, Isik S. Donor age and long-term culture affect differentiation and proliferation of human bone marrow mesenchymal stem cells. *Ann Hematol*. 2012;91(8):1175-1186. doi:10.1007/s00277-012-1438-x

**Study type: *in vitro* study.**

13. Kim M, Kim C, Choi YS, Kim M, Park C, Suh Y. Age-related alterations in mesenchymal stem cells related to shift in differentiation from osteogenic to adipogenic potential: implication to age-associated bone diseases and defects. *Mech Ageing Dev*. 2012;133(5):215-225. doi:10.1016/j.mad.2012.03.014

**Study type: Narrative Review; Level of evidence 4**

14. Shen J, Tsai YT, Dimarco NM, Long MA, Sun X, Tang L. Transplantation of mesenchymal stem cells from young donors delays aging in mice. *Sci Rep*. 2011;1:67. doi:10.1038/srep00067

**Study type: In vivo study**

15. Wei P, Bao R. Intra-Articular Mesenchymal Stem Cell Injection for Knee Osteoarthritis: Mechanisms and Clinical Evidence. *Int J Mol Sci*. 2022;24(1):59. doi:10.3390/ijms24010059

**Study type: Narrative Review; Level of evidence 4.**

16. Koh YG, Choi YJ. Infrapatellar fat pad-derived mesenchymal stem cell therapy for knee osteoarthritis. *Knee*. 2012;19(6):902-907. doi:10.1016/j.knee.2012.04.001

**Study type: Case-Control study; Level of evidence 3.**

17. Ferracini R, Alessio-Mazzola M, Sonzogni B, et al. Age and synovitis affect the results of the treatment of knee osteoarthritis with Microfragmented Autologous Fat Tissue. *Knee Surg Sports Traumatol Arthrosc*. 2023;31(9):3655-3664. doi:10.1007/s00167-022-07139-4

**Study type: Prospective Multicentric Cohort Study; Level of evidence 2.**

18. Vasso M, Corona K, Capasso L, Toro G, Schiavone Panni A. Intraarticular injection of microfragmented adipose tissue plus arthroscopy in isolated primary patellofemoral osteoarthritis is clinically effective and not affected by age, BMI, or stage of osteoarthritis. *J Orthop Traumatol*. 2022;23(1):7. doi:10.1186/s10195-022-00628-9

**Study type: Retrospective Cohort Study, Level of evidence 4.**

19. Borg TM, Heidari N, Noorani A, et al. Gender-Specific Response in Pain and Function to Biologic Treatment of Knee Osteoarthritis: A Gender-Bias-Mitigated, Observational, Intention-to-Treat Study at Two Years. *Stem Cells Int*. 2021;2021:6648437. doi:10.1155/2021/6648437

**Study type: Observational study; Level of evidence 3.**

**QUESTION 5**

**Could CBT for knee OA be used during the inflammatory phase when joint effusion is present (following effusion aspiration)?**

**Statement**

Current clinical evidence is lacking regarding the injection of CBT during the inflammatory phase in knee OA, as well as with regards to effusion aspiration prior to CBT injection.

However, multiple basic science studies have shown anti-inflammatory properties of CBT, which could support the rationale for its use during the inflammatory phase. While evidence is lacking with regards to the optimal timing of CBT injection for knee OA when effusion is present, **the consensus group recognizes that when present, effusion aspiration is likely beneficial in pain improvement and relieving functional limitations and also avoids dilution of the injected CBT product.** In addition, it could aid in replacing the inflammatory infiltrate in the knee with a more favorable one with anti-inflammatory properties.

Grade of recommendation: D

Rating Score: Mean 8.1±0.9; Median: 8 (6-9) Appropriate with relative agreement

*This statement is valid for both POC- and Expanded-CBT*

**Literature summary**

No clinical literature demonstrating the effect of CBT injection during the inflammatory phase in presence of joint effusion is available. However, basic and pre-clinical studies show a well-proven anti-inflammatory effect of CBT therapy, mostly due to the secretion of a plethora of different molecules called the cell secretome ^1^. Besides switching off inflammation, CBT acts in limiting stress response and apoptosis, and recruiting the immune and reparative cells of the recipient^2^. For example, *in vitro* study was performed on chondrocytes and synoviocytes in presence of high levels of pro-inflammatory mediators. A co-culture of adipose derived MSCs obtained from infrapatellar fat pad, subcutaneous hip, or abdominal fat was added in transwell and a decrease of IL-1ß, IL-6, and CXCL8/IL-8 expression was observed^3^. MSCs could also prevent chondrocytes from differentiating into fibrocytes and stimulate chondrocytes to produce type II collagen, thus providing help for tissue repair and regeneration^4^. The anabolic effect of the MSCs is mostly due to the growth factors they produce, such as transforming growth factor (TGF), hepatocyte growth factor (HGF), basic fibroblast growth factor (FGF) or vascular endothelial growth factor (VEGF), that induce proliferation and angiogenesis of various cell types ^5^. *In vitro* studies have shown that MSCs stimulate the chondrocyte production and reduce the expression of hypertrophic, fibrotic and inflammatory markers ^3,6,7^. Having in mind positive results of *in vitro* studies, there is also growing evidence on animal studies, providing evidence on the mechanisms and effects at the living tissue level ^8^. We are still not certain on how exactly do MSCs act once inside the joint, but the cessation of pain and functional improvement show that they do possess anti-inflammatory effect *in vivo* ^9^. MSCs therapy has shown favorable results in systematic reviews and meta-analysis of RCTs ^10–13^. Once injected into the joint, it is hard to predict the time length of MSCs survival, given the hostile microenvironment which they encounter, which is hypoxic, rich of inflammatory mediators, and with a low pH, being thus characterized by non-optimal conditions for MSC survival ^14^. So, it makes it rational to try to partially alter the environment by aspirating the effusion that contains inflammatory factors.

Certainly, aspiration also prevents the dilution of any injected substance, including the CBT. In a clinical trial on patients with knee OA that were injected by BM MSCs, 14 out of 15 patients had effusion on a baseline. Still, the results were favorable, with a long-lasting amelioration of pain, improvement of quality of life, and signs of cartilage repair. The paper does not report if the effusion was aspirated prior to injection^15^.

**References**:

1. Jeyaraman M, Muthu S, Ganie PA. Does the Source of Mesenchymal Stem Cell Have an Effect in the Management of Osteoarthritis of the Knee? Meta-Analysis of Randomized Controlled Trials. *Cartilage*. 2021;13(1_suppl):1532S-1547S. doi:10.1177/1947603520951623

**Study type: Meta-analysis; Level of evidence 2.**

2. Lopa S, Colombini A, Moretti M, de Girolamo L. Injective mesenchymal stem cell-based treatments for knee osteoarthritis: from mechanisms of action to current clinical evidences. *Knee Surg Sports Traumatol Arthrosc*. 2019;27(6):2003-2020. doi:10.1007/s00167-018-5118-9

**Study type: Systematic review; Level of evidence 3.**

3. Manferdini C, Maumus M, Gabusi E, et al. Adipose-derived mesenchymal stem cells exert antiinflammatory effects on chondrocytes and synoviocytes from osteoarthritis patients through prostaglandin E2. *Arthritis Rheum*. 2013;65(5):1271-1281. doi:10.1002/art.37908

**Study type: *In vitro* study**

4. Lu L, Dai C, Zhang Z, et al. Treatment of knee osteoarthritis with intra-articular injection of autologous adipose-derived mesenchymal progenitor cells: a prospective, randomized, double-blind, active-controlled, phase IIb clinical trial. *Stem Cell Res Ther*. 2019;10(1):143. doi:10.1186/s13287-019-1248-3

**Study type: RCT; Level of evidence 1.**

5. Pers YM, Ruiz M, Noël D, Jorgensen C. Mesenchymal stem cells for the management of inflammation in osteoarthritis: state of the art and perspectives. *Osteoarthritis Cartilage*. 2015;23(11):2027-2035. doi:10.1016/j.joca.2015.07.004

**Study type: Narrative review; Level of evidence 4.**

6. Wu L, Leijten J, van Blitterswijk CA, Karperien M. Fibroblast growth factor-1 is a mesenchymal stromal cell-secreted factor stimulating proliferation of osteoarthritic chondrocytes in co-culture. *Stem Cells Dev*. 2013;22(17):2356-2367. doi:10.1089/scd.2013.0118

**Study type: *In vitro* study**

7. Maumus M, Manferdini C, Toupet K, et al. Adipose mesenchymal stem cells protect chondrocytes from degeneration associated with osteoarthritis. *Stem Cell Res*. 2013;11(2):834-844. doi:10.1016/j.scr.2013.05.008

**Study type: *In vitro* study**

8. de Girolamo L, Filardo G, Laver L. Disease-modifying effects of orthobiologics in the treatment of knee osteoarthritis: the lesson learned from preclinical research models. *Knee Surg Sports Traumatol Arthrosc*. 2023;31(12):5286-5290. doi:10.1007/s00167-023-07423-x

**Study type: Editorial; Level of evidence 5.**

9. Liu M, Li K, Wang Y, Zhao G, Jiang J. Stem Cells in the Treatment of Neuropathic Pain: Research Progress of Mechanism. *Stem Cells Int*. 2020;2020:8861251. doi:10.1155/2020/8861251

**Study type: Narrative review; Level of evidence 4.**

10. Bolia IK, Bougioukli S, Hill WJ, et al. Clinical Efficacy of Bone Marrow Aspirate Concentrate Versus Stromal Vascular Fraction Injection in Patients With Knee Osteoarthritis: A Systematic Review and Meta-analysis. *Am J Sports Med*. 2022;50(5):1451-1461. doi:10.1177/03635465211014500

**Study type: Systematic Review and meta-analysis; Level of evidence 4**.

11. Kim KI, Kim MS, Kim JH. Intra-articular Injection of Autologous Adipose-Derived Stem Cells or Stromal Vascular Fractions: Are They Effective for Patients With Knee Osteoarthritis? A Systematic Review With Meta-analysis of Randomized Controlled Trials. *Am J Sports Med*. 2023;51(3):837-848. doi:10.1177/03635465211053893

**Study type: Systematic Review and Meta-analysis; Level of evidence 1.**

12. Wei P, Bao R. Intra-Articular Mesenchymal Stem Cell Injection for Knee Osteoarthritis: Mechanisms and Clinical Evidence. *Int J Mol Sci*. 2022;24(1):59. doi:10.3390/ijms24010059

**Study type: Narrative Review; Level of evidence 4.**

13. Freitag J, Wickham J, Shah K, Tenen A. Real-world evidence of mesenchymal stem cell therapy in knee osteoarthritis: a large prospective two-year case series. *Regen Med*. 2022;17(6):355-373. doi:10.2217/rme-2022-0002

**Study type: Prospective, case series; Level of evidence 4.**

14. Hyun JS, Tran MC, Wong VW, et al. Enhancing stem cell survival in vivo for tissue repair. *Biotechnol Adv*. 2013;31(5):736-743. doi:10.1016/j.biotechadv.2012.11.003

**Study type: Narrative review; Level of evidence 4.**

15. Soler R, Orozco L, Munar A, et al. Final results of a phase I-II trial using ex vivo expanded autologous Mesenchymal Stromal Cells for the treatment of osteoarthritis of the knee confirming safety and suggesting cartilage regeneration. *Knee*. 2016;23(4):647-654. doi:10.1016/j.knee.2015.08.013

**Study type: RTC; Level of evidence 2.**

**QUESTION 6**

**Are there specific contraindications for use of CBT for knee OA?**

**Statement**

Apart from the generally accepted contraindications for any knee injection, the consensus group attempted to identify and highlight specific contraindications related to CBT injections. In the absence of formal contraindications documented in the existing literature for the application of CBT in the treatment of knee OA, the consensus group’s focus has been directed towards the identification of exclusion criteria employed in clinical trials with a level of evidence I. These identified exclusion criteria provide essential guidance when considering the suitability of patients for CBT in the treatment of knee OA. **The consensus group agrees that caution should guide any decision-making process when considering the use of CBT, helping to ensure patient safety as well as the appropriate selection of candidates for clinical interventions.** This is relevant in conditions such as pregnancy, in which there’s no available evidence on the safety of CBT use; therefore, the consensus group recommends avoiding its use in these instances until further data is available.

Since most of suggested contraindications have not been thoroughly or sufficiently studied, the consensus group chose to recommend caution also in the presence of co-existent malignancies or systemic conditions due to the possibility of unknown interactions.

While it remains challenging to ascertain whether these exclusion criteria are specific to CBT, the most frequently described criteria for exclusion from these clinical trials were categorized based on local related contraindications, harvest site associated contraindications, and systemic related contraindications.

*Contraindications due to systemic problems:*

- *Malignancies/Cancer*

In terms of malignancies, current literature has not demonstrated a clear link between CBT use in the knee joint and the risk of tumor proliferation, either locally or remotely. However, due to the theoretical risk that CBT may contribute to tumor growth promotion in situations where either a benign or malignant tumor exists in the knee joint, the consensus group considers these conditions a contraindication for injecting CBT. Due to similar concerns and until further evidence is available, the consensus group recommends this should also apply to tumors with or without existing metastasis located in other locations, outside/even remote from the knee, although consultation should be made with the managing oncologist/physician in specific cases.

- *Inflammatory diseases*

The presence of local or systemic inflammatory diseases (rheumatoid arthritis, Crohn's disease and or other auto-immune diseases) should warrant caution when injecting CBT in the knee due to the potential of enhancing the inflammatory response in the joint, especially with regards to allogeneic cell sources.

- *Autoimmune disorders*

Patients with active autoimmune disorders undergoing immunosuppressive treatment or suffering from immune deficiency.

*Contraindications related to harvest site:*

- *Coagulopathy or regular use of anticoagulants*

This may cause harvest associated hematoma and bleeding related complications. This is a relative contraindication due to variability in coagulopathies as well as anti-coagulation use as it may be stopped in certain scenarios. For this purpose, the consensus group recommends consultation with a haematologist in cases of existing coagulopathies or regular use of anticoagulants prior to commencing with CBT use with regards to potential harvest site associated complications.

- *Abdominal Hernia*

This is a contraindication for abdominal harvesting for Adipose CBT. In these cases, another harvest site can be used (i.e buttocks, lower back).

Grade of recommendation: D

Rating Score: Mean 8.0±1.0; Median 8 (5-9) Appropriate with relative agreement

*This statement is valid for both POC- and Expanded-CBT*

**Literature summary**

As the literature does not describe any formal contraindications to the use of cell therapies in the treatment of knee osteoarthritis, the contraindications for the use of CBT are registered as exclusion criteria for patient eligibility from clinical trials. We focused on exclusion criteria from clinical trials with the highest level of evidence in comparison with hyaluronic acid to consider a non-cell based control.

The identified studies describe the use of adipose-derived stromal vascular fraction^1,2^, bone marrow aspirate concentrate^3,4^, amniotic suspension allograft ^5^ or expanded mesenchymal stem cells (MSC) from bone marrow^6,7^, umbilical cord^8^, peripheral blood^9,10^, adipose tissue^11^. Although it remains difficult to assess whether they could be specific for a given CBT, the most reported exclusion criteria were the history of previous or active malignancy^1–6,8,9,11^, followed by presence of infections sign or positive serology for HIV, hepatitis or syphilis^1,3–6,8,11^. Systemic disorders referring to autoimmune disorders^3,5,6,9,10^, coagulation disorder^1,2,4,5,11^, diabetes^3,9^, or hematological disease^3^ were also regularly reported as exclusion criteria. Active immunosuppressive treatment^2,5–7^ or solid organ/hematologic transplantation^5^ are also considered as exclusion criteria. Severe heart, lung, liver or kidney disease preventing patients to get a general anesthesia is also mentioned^1–3^ in reference to large volume liposuction or bone marrow harvesting.

These trials also reported classical exclusion criteria for injectable knee OA treatment as valgus or varus deformities as evidenced by standard-of-care radiograph, or inflammatory joint disease^3,6,8–10^.

**References**

1. Hong Z, Chen J, Zhang S, et al. Intra-articular injection of autologous adipose-derived stromal vascular fractions for knee osteoarthritis: a double-blind randomized self-controlled trial. *Int Orthop*. 2019;43(5):1123-1134. doi:10.1007/s00264-018-4099-0

**Study type: RCT; Level of evidence 1.**

2. Zhang Y, Bi Q, Luo J, Tong Y, Yu T, Zhang Q. The Effect of Autologous Adipose-Derived Stromal Vascular Fractions on Cartilage Regeneration Was Quantitatively Evaluated Based on the 3D-FS-SPGR Sequence: A Clinical Trial Study. *Biomed Res Int*. 2022;2022:2777568. doi:10.1155/2022/2777568

**Study type: RCT; Level of evidence 1.**

3. Goncars V, Jakobsons E, Blums K, et al. The comparison of knee osteoarthritis treatment with single-dose bone marrow-derived mononuclear cells vs. hyaluronic acid injections. *Medicina (Kaunas)*. 2017;53(2):101-108. doi:10.1016/j.medici.2017.02.002

**Study type: RCT; Level of evidence 2.**

4. Dulic O, Rasovic P, Lalic I, et al. Bone Marrow Aspirate Concentrate versus Platelet Rich Plasma or Hyaluronic Acid for the Treatment of Knee Osteoarthritis. *Medicina (Kaunas)*. 2021;57(11):1193. doi:10.3390/medicina57111193

**Study type: RCT; Level of evidence 2.**

5. Gomoll AH, Farr J, Cole BJ, et al. Safety and Efficacy of an Amniotic Suspension Allograft Injection Over 12 Months in a Single-Blinded, Randomized Controlled Trial for Symptomatic Osteoarthritis of the Knee. *Arthroscopy*. 2021;37(7):2246-2257. doi:10.1016/j.arthro.2021.02.044

**Study type: RCT; Level of evidence 1.**

6. Vega A, Martín-Ferrero MA, Del Canto F, et al. Treatment of Knee Osteoarthritis With Allogeneic Bone Marrow Mesenchymal Stem Cells: A Randomized Controlled Trial. *Transplantation*. 2015;99(8):1681-1690. doi:10.1097/TP.0000000000000678

**Study type: RCT; Level of evidence 2.**

7. Wong KL, Lee KBL, Tai BC, Law P, Lee EH, Hui JHP. Injectable cultured bone marrow-derived mesenchymal stem cells in varus knees with cartilage defects undergoing high tibial osteotomy: a prospective, randomized controlled clinical trial with 2 years’ follow-up. *Arthroscopy*. 2013;29(12):2020-2028. doi:10.1016/j.arthro.2013.09.074

**Study type: RCT; Level of evidence 2.**

8. Matas J, Orrego M, Amenabar D, et al. Umbilical Cord-Derived Mesenchymal Stromal Cells (MSCs) for Knee Osteoarthritis: Repeated MSC Dosing Is Superior to a Single MSC Dose and to Hyaluronic Acid in a Controlled Randomized Phase I/II Trial. *Stem Cells Transl Med*. 2019;8(3):215-224. doi:10.1002/sctm.18-0053

**Study type: RCT; Level of evidence 2.**

9. Turajane T, Chaveewanakorn U, Fongsarun W, Aojanepong J, Papadopoulos KI. Avoidance of Total Knee Arthroplasty in Early Osteoarthritis of the Knee with Intra-Articular Implantation of Autologous Activated Peripheral Blood Stem Cells versus Hyaluronic Acid: A Randomized Controlled Trial with Differential Effects of Growth Factor Addition. *Stem Cells Int*. 2017;2017:8925132. doi:10.1155/2017/8925132

**Study type: RCT; Level of evidence 2.**

10. Saw KY, Anz A, Siew-Yoke Jee C, et al. Articular cartilage regeneration with autologous peripheral blood stem cells versus hyaluronic acid: a randomized controlled trial. *Arthroscopy*. 2013;29(4):684-694. doi:10.1016/j.arthro.2012.12.008

**Study type: RCT; Level of evidence 2.**

11. Lu L, Dai C, Zhang Z, et al. Treatment of knee osteoarthritis with intra-articular injection of autologous adipose-derived mesenchymal progenitor cells: a prospective, randomized, double-blind, active-controlled, phase IIb clinical trial. *Stem Cell Res Ther*. 2019;10(1):143. doi:10.1186/s13287-019-1248-3

**Study type: RCT; Level of evidence 1.**

**QUESTION 7**

**Does CBT induce disease-modifying effects in knee OA?**

**Statement**

Clinical evidence on the disease-modifying effects of CBT for knee OA derived from adipose tissue, bone marrow, and fetal annexes (placenta, amnion, umbilical cord/umbilical cord blood) remains limited and inconsistent. Preclinical studies provide promising insights into their potential for disease modification in knee OA. A large number of animal studies show disease-modifying effects of CBT derived from the aforementioned tissue sources in animal OA models. Specifically, positive results have been reported in macroscopic, histological, and immunohistochemical analyses at both cartilage and synovial levels, with superior effects associated with adipose-derived products over the other two. **However, currently, there is no sufficient direct evidence to suggest that CBT induces disease-modifying effects in humans.**

Grade of recommendation: C

Rating Score: Mean 8.4±0.7; Median 8.5 (7-9) Appropriate with strong agreement

*This statement is valid for both POC- and Expanded-CBT*

**Literature summary**

*Clinical findings*

There is still limited clinical evidence on possible disease-modifying effects of CBT derived from adipose tissue, bone marrow fand fetal annexes for the treatment of knee OA. Most research conducted to date on these products has involved only small groups of patients and often lacked a proper control group, making it challenging to establish the true efficacy of these therapies in preventing OA disease progression. Also, the few high-level studies present scarce and inconsistent findings.

Concerning adipose tissue, Hong et al. evaluated in a double-blind RCT 16 patients with bilateral knee OA treated in one knee with an injection of autologous adipose-derived stromal vascular fraction (SVF) and in the other knee with a hyaluronic acid injection. The analysis of the Magnetic Resonance Observation of Cartilage Repair Tissue (MOCART) score and the whole-organ magnetic resonance imaging score (WORMS) revealed at 12 months a significant improvement of articular cartilage status in SVF-treated knees compared to controls^1^. Freitag et al. assessed in a RCT 30 patients with knee OA treated with a single or two injections of AT-MSCs (1 × 10^8^) compared to a control group of conservative management. Imaging analysis with the MRI Osteoarthritis Knee Scores (MOAKS) score at 12 months of follow-up demonstrated a higher progression of cartilage loss from baseline in the control group (67%) compared to the one-injection group (30%) and the two-injection group (11%)^2^. Lee et al. evaluated in a double-blind RCT 12 patients with bilateral knee OA treated in one knee with an AT-MSCs (1 × 10^8^) injection and in the other knee with a saline injection. At 6 months of follow-up, while no differences in Kellgren-Lawrence grade and joint space width were observed at radiography evaluation, the MR analysis showed an increased cartilage defect in the control group compared to the treatment group^3^. Lu et al. investigated in a double-blind RCT 53 patients with knee OA treated with a single intra-articular injection of AT-MSCs or hyaluronic acid. At 12 months after the injection, the AT-MSC group demonstrated an increase in articular cartilage volume at MR analysis, compared to the hyaluronic acid group showing a decrease in cartilage volume^4^. A case-series analysis conducted on 32 knees demonstrated increased cartilage quality at MRI with dGEMRIC protocol after an injection of microfragmented adipose tissue at 12 months follow-up. In particular the Authors reported structural and biochemical changes in the cartilage with an increased GAG production if compared to baseline^5^.

Among bone marrow studies, Soler et al. evaluated in a phase I trial 15 patients treated with an intra-articular injection of BM-MSCs. The MR analysis with T2 mapping performed at baseline, 6 and 12 months showed signs of cartilage regeneration over time in all patients^6^. Similarly, Al-Najar et al. assessed in a phase I prospective clinical study 13 patients suffering from grade 2-3 knee OA undergoing two intra-articular injections of BMMSCs with 1-month interval. The quantitative MR analysis with T2 mapping documented a significant improvement in cartilage thickness at 12 months of follow-up^7^. Gupta et al. performed a phase 3, double-blind, multicenter RCT on 146 patients with grade 2-3 knee OA treated with a single intra-articular injection of BM-MSCs (25 million cells) or placebo, followed by 20 mg per 2 mL of hyaluronic acid. Even though the cartilage volume did not show significant differences between the two groups, the MR analysis with T2 mapping showed that there was no deep cartilage worsening in the medial femorotibial compartment of the knee in the BM-MSC group at the 12-month follow-up, whereas in the placebo group there was significant and gradual cartilage worsening^8^. Silvestre et al. conducted a retrospective comparative study on patients affected by patellofemoral OA analyzing their MR performed at baseline and at 12 months of follow-up using T2 mapping sequences. In detail, 96 patients treated with intra-articular injection of BMAC were compared to 21 patients who did not undergo any knee injection nor surgical treatment during the previous year. MR analysis at 12 months of follow-up showed in the BMAC group no statistically significant difference in hyaline cartilage volume compared to baseline, while a significant decrease in the cartilage volume was documented in the control group^9^.

Some authors reported disease-modifying effects of CBT derived from bone marrow also directly targeting the subchondral bone area, which plays a critical role in both the pathophysiology and progression of knee OA. Hernigou et al. evaluated in a RCT the effects of intra-articular BMAC injections versus subchondral BMAC injections in 60 patients affected by bilateral symptomatic knee OA. Radiographic evaluations performed up to 24 months highlighted lower joint space narrowing and disease progression in the subchondral group. Moreover, MR analysis demonstrated an increase in percentage cartilage volume on the medial compartment in the subchondral group while a decrease was observed in the intra-articular group, suggesting that subchondral BMAC injections can halt the progressive cartilage loss observed in OA patients^10^. Kon et al. evaluated in a pilot prospective multi-center study the combined use of intra-articular and subchondral bone BMAC injections for the treatment of 30 knee OA patients, performing MR analysis at baseline and 12 months after treatment. The WORMS revealed at 12 months a significant improvement of the bone marrow edema, while the remaining MR parameters including cartilage volume did not show any significant changes after injection, neither as improvement nor as signs of disease progression^11^.

Regarding CBT derived from fetal annexes, Soltani et al.^12^ in a double-blind, placebo-controlled RCT investigating the safety and efficacy of intra-articular injection of culture-expanded placental mesenchymal stem cells in moderate to severe knee OA reported, in addition to subjective improvements, chondral thickening in about 10% of the total knee joint area in the intervention group after 24 weeks. On the other hand, the RCT of Matas et al. comparing umbilical cord-MSC to HA^13^ failed to demonstrate change from baseline or among groups in any of the 14 items composing the MRI-WORMS score, albeit MSC-treated patients experienced significant pain and function improvements from baseline to 12 months follow-up, with significant superiority compared to HA.

*Pre-clinical findings (animal studies)*

Evidence of disease-modifying effects has been documented in preclinical studies involving adipose- or bone marrow-derived cell-based products on animal OA models. A recent systematic review of preclinical studies conducted by the ESSKA-ORBIT Initiative identified 71 animal studies analyzing disease-modifying effects of CBT derived from adipose tissue for the treatment of OA^14^. Positive results have been documented at macroscopic, histological, and immunohistochemical level, as well as in the evaluation of biomarkers, clinical, and imaging results in 90% of the evaluated studies. In particular, CBT derived from adipose tissue provided disease-modifying effects at cartilage level compared to OA controls documenting: better results in terms of macroscopic cartilage appearance, with a smoother articular surface with less erosion, fibrillation, and osteophytes^15–17^; better results in terms of histological findings, with improved cartilage thickness and chondrocyte arrangement, reduced extracellular matrix loss, and increased cartilage-specific matrix^15,18,19^; better results in terms of immunohistochemical features, with increased expression of chondrogenic and cell proliferation markers, while reducing fibro-cartilaginous, catabolic, apoptotic, and inflammatory markers^17,18,20^. CBT derived from adipose tissue also provided disease-modifying effects at synovial level, with improvement in synovitis status compared to OA controls, with a reduction in the thickness of the lining layer of the synovial membrane and a decrease of inflammatory cell infiltration^21–23^.

Likewise adipose-derived products, a recent systematic review of preclinical studies on animal OA models conducted by the ESSKA-ORBIT Initiative identified 53 studies focusing on the disease-modifying effects of CBT derived from bone marrow for the treatment of OA^24^. In addition to clinical effects, 85% of the studies included in the systematic review reports disease-modifying effects induced by intra-articular injections of bone marrow-derived CBT in the treatment of OA. Data show that CBT were able to slow down the progression of cartilage damage with benefits at macroscopic, histological, and immunohistochemical levels. In detail, less articular degeneration was macroscopically observed in joints receiving CBT derived from bone marrow injections, with better cartilage volume and thickness, relatively smooth articular surface, and less osteophytes formation ^25,26^. Histological analysis revealed increased chondrocyte count, improved cell organization, and higher density, along with abundant extracellular matrix and superior proteoglycan and glycosaminoglycan content compared to OA controls^24,27,28^. Immunohistochemical evaluations revealed an increased expression of aggrecan and collagen type-II alpha and a decreased expression of collagen type-I alpha ^27,28^. Finally, positive results have been also observed in terms of clinical and imaging findings, as well as in the modulation of inflammatory and cartilage biomarkers, while poor effects have been described at the synovial membrane level^29–31^.

Promising evidence on disease-modifying effects provided by CBT derived from fetal sources has been also reported in preclinical studies on animal OA models. Several studies analyzed the injective use of expanded UC-MSCs in animal OA joints, documenting positive disease-modifying effects at macroscopic, histological, and immunohistochemical levels. Specifically, animals treated with UC-MSCs injections presented an improved cartilage status compared to OA controls, with better articular surface, less cartilage fibrillation, higher chondrocyte count, up-regulation of aggrecan and type 2 collagen, and lower staining of IL-1β, TNFα, MMP13, and ADAMTS5^32–35^. Positive disease-modifying effects after UC-MSCs injections have been also reported at the synovial level, with lower inflammatory cell infiltration and hyperplasia compared to OA controls^36,37^. Satisfactory disease-modifying effects have been documented also for placental tissue-derived injectable products in preclinical studies on animal OA models, focusing on expanded MSCs or “point of care” products. Overall positive results were documented after expanded placental MSCs injections compared to OA controls, with significantly lower cartilage degeneration and articular fibrillation, a higher staining of type 2 collagen and Sox-9, a lower staining of MMP13, ADAMT4, and ADAMT5, and better synovitis scores^38–40^. Conversely, the injections of “point of care” products such as the amniotic suspension allograft (ASA) did not report optimal findings, with minimal effects on cartilage tissue and worse results compared to OA controls at synovial level, with a documented increase in synovitis and fibrosis after ASA injections^41,42^

Comparing the three main sources of cells, adipose-derived product showed a more pronounced disease modifying effect over the others, with particular reference to changes in OA biomarkers and synovium aspects.

**References**

1. Hong Z, Chen J, Zhang S, et al. Intra-articular injection of autologous adipose-derived stromal vascular fractions for knee osteoarthritis: a double-blind randomized self-controlled trial. *Int Orthop*. 2019;43(5):1123-1134. doi:10.1007/s00264-018-4099-0

**Study type: RCT; Level of evidence 1.**

2. Freitag J, Bates D, Wickham J, et al. Adipose-derived mesenchymal stem cell therapy in the treatment of knee osteoarthritis: a randomized controlled trial. *Regen Med*. 2019;14(3):213-230. doi:10.2217/rme-2018-0161

**Study type: RCT; Level of evidence 2.**

3. Lee WS, Kim HJ, Kim KI, Kim GB, Jin W. Intra-Articular Injection of Autologous Adipose Tissue-Derived Mesenchymal Stem Cells for the Treatment of Knee Osteoarthritis: A Phase IIb, Randomized, Placebo-Controlled Clinical Trial. *Stem Cells Transl Med*. 2019;8(6):504-511. doi:10.1002/sctm.18-0122

**Study type: RCT; Level of evidence 2.**

4. Lu L, Dai C, Zhang Z, et al. Treatment of knee osteoarthritis with intra-articular injection of autologous adipose-derived mesenchymal progenitor cells: a prospective, randomized, double-blind, active-controlled, phase IIb clinical trial. *Stem Cell Res Ther*. 2019;10(1):143. doi:10.1186/s13287-019-1248-3

**Study type: RCT; Level of evidence 1.**

5. Hudetz D, Borić I, Rod E, et al. The Effect of Intra-articular Injection of Autologous Microfragmented Fat Tissue on Proteoglycan Synthesis in Patients with Knee Osteoarthritis. *Genes (Basel)*. 2017;8(10):270. doi:10.3390/genes8100270

**Study type: Case Series; Level of evidence 4.**

6. Soler R, Orozco L, Munar A, et al. Final results of a phase I-II trial using ex vivo expanded autologous Mesenchymal Stromal Cells for the treatment of osteoarthritis of the knee confirming safety and suggesting cartilage regeneration. *Knee*. 2016;23(4):647-654. doi:10.1016/j.knee.2015.08.013

**Study type: RCT; Level of evidence 2.**

7. Al-Najar M, Khalil H, Al-Ajlouni J, et al. Intra-articular injection of expanded autologous bone marrow mesenchymal cells in moderate and severe knee osteoarthritis is safe: a phase I/II study. *J Orthop Surg Res*. 2017;12(1):190. doi:10.1186/s13018-017-0689-6

**Study type: Prospective uncontrolled clinical trial; Level of evidence 3.**

8. Gupta PK, Maheshwari S, Cherian JJ, et al. Efficacy and Safety of Stempeucel in Osteoarthritis of the Knee: A Phase 3 Randomized, Double-Blind, Multicenter, Placebo-Controlled Study. *Am J Sports Med*. 2023;51(9):2254-2266. doi:10.1177/03635465231180323

**Study type: RCT; Level of evidence 1.**

9. Silvestre A, Bise S, Delavigne C, et al. Intra-articular Injection of Bone Marrow Concentrate for Treatment of Patellofemoral Osteoarthritis: Preliminary Results Utilizing an Ultrasound-Guided Marrow Harvesting Technique. *J Vasc Interv Radiol*. 2023;34(1):71-78.e1. doi:10.1016/j.jvir.2022.10.006

**Study type: Retrospective Study; Level of evidence 4.**

10. Hernigou P, Bouthors C, Bastard C, Flouzat Lachaniette CH, Rouard H, Dubory A. Subchondral bone or intra-articular injection of bone marrow concentrate mesenchymal stem cells in bilateral knee osteoarthritis: what better postpone knee arthroplasty at fifteen years? A randomized study. *Int Orthop*. 2021;45(2):391-399. doi:10.1007/s00264-020-04687-7

**Study type: RCT; Level of evidence 2.**

11. Kon E, Boffa A, Andriolo L, et al. Subchondral and intra-articular injections of bone marrow concentrate are a safe and effective treatment for knee osteoarthritis: a prospective, multi-center pilot study. *Knee Surg Sports Traumatol Arthrosc*. 2021;29(12):4232-4240. doi:10.1007/s00167-021-06530-x

**Study type: Pilot Study; Level of evidence 3.**

12. Khalifeh Soltani S, Forogh B, Ahmadbeigi N, et al. Safety and efficacy of allogenic placental mesenchymal stem cells for treating knee osteoarthritis: a pilot study. *Cytotherapy*. 2019;21(1):54-63. doi:10.1016/j.jcyt.2018.11.003

**Study type: Double-blind placebo controlled RCT; Level of evidence 2.**

13. Matas J, Orrego M, Amenabar D, et al. Umbilical Cord-Derived Mesenchymal Stromal Cells (MSCs) for Knee Osteoarthritis: Repeated MSC Dosing Is Superior to a Single MSC Dose and to Hyaluronic Acid in a Controlled Randomized Phase I/II Trial. *Stem Cells Transl Med*. 2019;8(3):215-224. doi:10.1002/sctm.18-0053

**Study type: RCT; Level of evidence 2.**

14. Perucca Orfei C, Boffa A, Sourugeon Y, et al. Cell-based therapies have disease-modifying effects on osteoarthritis in animal models. A systematic review by the ESSKA Orthobiologic Initiative. Part 1: adipose tissue-derived cell-based injectable therapies. *Knee Surg Sports Traumatol Arthrosc*. 2023;31(2):641-655. doi:10.1007/s00167-022-07063-7

**Study type: Systematic Review; Level of evidence 4.**

15. Mei L, Shen B, Ling P, et al. Culture-expanded allogenic adipose tissue-derived stem cells attenuate cartilage degeneration in an experimental rat osteoarthritis model. *PLoS One*. 2017;12(4):e0176107. doi:10.1371/journal.pone.0176107

**Study type: *in vivo* study.**

16. Veronesi F, Berni M, Marchiori G, et al. Evaluation of cartilage biomechanics and knee joint microenvironment after different cell-based treatments in a sheep model of early osteoarthritis. *Int Orthop*. 2021;45(2):427-435. doi:10.1007/s00264-020-04701-y

**Study type: *in vivo* study.**

17. Mei L, Shen B, Xue J, et al. Adipose tissue-derived stem cells in combination with xanthan gum attenuate osteoarthritis progression in an experimental rat model. *Biochem Biophys Res Commun*. 2017;494(1-2):285-291. doi:10.1016/j.bbrc.2017.10.039

**Study type: *in vivo* study.**

18. Desando G, Cavallo C, Sartoni F, et al. Intra-articular delivery of adipose derived stromal cells attenuates osteoarthritis progression in an experimental rabbit model. *Arthritis Res Ther*. 2013;15(1):R22. doi:10.1186/ar4156

**Study type: *in vivo* study.**

19. Shin K, Cha Y, Ban YH, et al. Anti-osteoarthritis effect of a combination treatment with human adipose tissue-derived mesenchymal stem cells and thrombospondin 2 in rabbits. *World J Stem Cells*. 2019;11(12):1115-1129. doi:10.4252/wjsc.v11.i12.1115

**Study type: *in vivo* study.**

20. Dubey NK, Wei HJ, Yu SH, et al. Adipose-derived Stem Cells Attenuates Diabetic Osteoarthritis via Inhibition of Glycation-mediated Inflammatory Cascade. *Aging Dis*. 2019;10(3):483-496. doi:10.14336/AD.2018.0616

**Study type: *in vivo* study.**

21. Hsu GCY, Cherief M, Sono T, et al. Divergent effects of distinct perivascular cell subsets for intra-articular cell therapy in posttraumatic osteoarthritis. *J Orthop Res*. 2021;39(11):2388-2397. doi:10.1002/jor.24997

**Study type: *in vivo* study.**

22. Cheng JH, Yen KT, Chou WY, et al. Autologous Adipose-Derived Mesenchymal Stem Cells Combined with Shockwave Therapy Synergistically Ameliorates the Osteoarthritic Pathological Factors in Knee Joint. *Pharmaceuticals (Basel)*. 2021;14(4):318. doi:10.3390/ph14040318

**Study type: *in vivo* study.**

23. ter Huurne M, Schelbergen R, Blattes R, et al. Antiinflammatory and chondroprotective effects of intraarticular injection of adipose-derived stem cells in experimental osteoarthritis. *Arthritis Rheum*. 2012;64(11):3604-3613. doi:10.1002/art.34626

**Study type: *in vivo* study.**

24. Boffa A, Perucca Orfei C, Sourugeon Y, et al. Cell-based therapies have disease-modifying effects on osteoarthritis in animal models. A systematic review by the ESSKA Orthobiologic Initiative. Part 2: bone marrow-derived cell-based injectable therapies. *Knee Surg Sports Traumatol Arthrosc*. 2023;31(8):3230-3242. doi:10.1007/s00167-023-07320-3

**Study type: Systematic Review; Level of evidence 4.**

25. Al Faqeh H, Nor Hamdan BMY, Chen HC, Aminuddin BS, Ruszymah BHI. The potential of intra-articular injection of chondrogenic-induced bone marrow stem cells to retard the progression of osteoarthritis in a sheep model. *Exp Gerontol*. 2012;47(6):458-464. doi:10.1016/j.exger.2012.03.018

**Study type: *in vivo* study.**

26. Wang Z, Zhai C, Fei H, et al. Intraarticular injection autologous platelet-rich plasma and bone marrow concentrate in a goat osteoarthritis model. *J Orthop Res*. Published online February 21, 2018. doi:10.1002/jor.23877

**Study type: *in vivo* study.**

27. Bhatti FU, Mehmood A, Latief N, et al. Vitamin E protects rat mesenchymal stem cells against hydrogen peroxide-induced oxidative stress in vitro and improves their therapeutic potential in surgically-induced rat model of osteoarthritis. *Osteoarthritis Cartilage*. 2017;25(2):321-331. doi:10.1016/j.joca.2016.09.014

**Study type: *in vivo* study.**

28. Desando G, Bartolotti I, Cavallo C, et al. Short-Term Homing of Hyaluronan-Primed Cells: Therapeutic Implications for Osteoarthritis Treatment. *Tissue Eng Part C Methods*. 2018;24(2):121-133. doi:10.1089/ten.TEC.2017.0336

**Study type: *in vivo* study.**

29. Huang H, Zhang P, Xiang C, Zeng C, Du Q, Huang W. Effect of bone marrow mesenchymal stem cell transplantation combined with lugua polypeptide injection on osteoarthritis in rabbit knee joint. *Connect Tissue Res*. 2022;63(4):370-381. doi:10.1080/03008207.2021.1962314

**Study type: *in vivo* study.**

30. Barrachina L, Remacha AR, Romero A, et al. Assessment of effectiveness and safety of repeat administration of proinflammatory primed allogeneic mesenchymal stem cells in an equine model of chemically induced osteoarthritis. *BMC Vet Res*. 2018;14(1):241. doi:10.1186/s12917-018-1556-3

**Study type: *in vivo* study.**

31. Jin Y, Xu M, Zhu H, et al. Therapeutic effects of bone marrow mesenchymal stem cells-derived exosomes on osteoarthritis. *J Cell Mol Med*. 2021;25(19):9281-9294. doi:10.1111/jcmm.16860

**Study type: *in vivo* study.**

32. Bie Y, Chen Q, Xu J, et al. Human umbilical-cord-derived mesenchymal stem cells in combination with rapamycin reduce cartilage degradation via inhibition of the *AKT/mTOR* signaling pathway. *Immunopharmacology and Immunotoxicology*. 2023;45(5):549-557. doi:10.1080/08923973.2023.2189062

**Study type: *in vivo* study.**

33. Kim H, Yang G, Park J, Choi J, Kang E, Lee BK. Therapeutic effect of mesenchymal stem cells derived from human umbilical cord in rabbit temporomandibular joint model of osteoarthritis. *Sci Rep*. 2019;9(1):13854. doi:10.1038/s41598-019-50435-2

**Study type: *in vivo* study.**

34. Geng Y, Chen J, Alahdal M, et al. Intra-articular injection of hUC-MSCs expressing miR-140-5p induces cartilage self-repairing in the rat osteoarthritis. *J Bone Miner Metab*. 2020;38(3):277-288. doi:10.1007/s00774-019-01055-3

**Study type: *in vivo* study.**

35. Mostafa A, Korayem HE, Fekry E, Hosny S. The Effect of Intra-articular versus Intravenous Injection of Mesenchymal Stem Cells on Experimentally-Induced Knee Joint Osteoarthritis. *J Microsc Ultrastruct*. 2021;9(1):31-38. doi:10.4103/JMAU.JMAU_2_20

**Study type: *in vivo* study.**

36. Cheng JH, Wang CJ, Chou WY, Hsu SL, Chen JH, Hsu TC. Comparison efficacy of ESWT and Wharton’s jelly mesenchymal stem cell in early osteoarthritis of rat knee. *Am J Transl Res*. 2019;11(2):586-598.

**Study type: *in vivo* study.**

37. Hsu CC, Cheng JH, Wang CJ, Ko JY, Hsu SL, Hsu TC. Shockwave Therapy Combined with Autologous Adipose-Derived Mesenchymal Stem Cells Is Better than with Human Umbilical Cord Wharton’s Jelly-Derived Mesenchymal Stem Cells on Knee Osteoarthritis. *Int J Mol Sci*. 2020;21(4):1217. doi:10.3390/ijms21041217

**Study type: *in vivo* study.**

38. Sampath SJP, Kotikalapudi N, Venkatesan V. A novel therapeutic combination of mesenchymal stem cells and stigmasterol to attenuate osteoarthritis in rodent model system-a proof of concept study. *Stem Cell Investig*. 2021;8:5. doi:10.21037/sci-2020-048

**Study type: *in vivo* study.**

39. Marino-Martínez IA, Martínez-Castro AG, Peña-Martínez VM, et al. Human amniotic membrane intra-articular injection prevents cartilage damage in an osteoarthritis model. *Exp Ther Med*. 2019;17(1):11-16. doi:10.3892/etm.2018.6924

**Study type: *in vivo* study.**

40. Kim J ki, Kim T hoon, Park S won, et al. Protective effects of human placenta extract on cartilage degradation in experimental osteoarthritis. *Biol Pharm Bull*. 2010;33(6):1004-1010. doi:10.1248/bpb.33.1004

**Study type: *in vivo* study.**

41. Kimmerling KA, Gomoll AH, Farr J, Mowry KC. Amniotic suspension allograft improves pain and function in a rat meniscal tear-induced osteoarthritis model. *Arthritis Res Ther*. 2022;24(1):63. doi:10.1186/s13075-022-02750-9

**Study type: *in vivo* study.**

42. Kimmerling KA, Gomoll AH, Farr J, Mowry KC. Amniotic Suspension Allograft Modulates Inflammation in a Rat Pain Model of Osteoarthritis. *J Orthop Res*. 2020;38(5):1141-1149. doi:10.1002/jor.24559

**Study type: *in vivo* study.**

**QUESTION 8**

**Is a repeated cycle of CBT injections recommended following a previous successful CBT treatment for knee OA upon the re-emergence of symptoms?**

**Statement**

Current evidence regarding repeated cycles of CBT treatment for knee OA is limited. However, it has been suggested that this strategy may have clinical benefits. **As evidence suggests a decrease in the effects of CBT for knee OA over time, the consensus group agrees that an additional cycle could be considered upon the re-emergence of symptoms after a previous successful treatment (lasting around 12 months).** However, the available data are mainly related to autologous procedures. Concerning allogeneic procedures, a few preclinical studies have indicated a local immune response with potential destruction of injected mesenchymal stem/stromal cells (MSCs), while a few clinical studies have indicated a potential higher risk of mild local adverse events when using allogeneic cells for repeated treatments.

Grade of recommendation: D

Rating Score: Mean 8.2±0.9; Median 8 (6-9) Appropriate with relative agreement

*This statement is valid for both POC- and Expanded-CBT*

**Literature summary**

Several studies pointed out that MSC intra-articular therapy is a safe procedure when treating knee OA, both in short^1,2^ and medium term^3^.

Autologous and allogeneic CBT will be treated separately, as they present differences in terms of amount of evidence, as well as of possible involvement of an immune response.

*Autologous CBT*

In a recent systematic review from 2021, Wiggers^4^ has also found improvements in clinical and radiological outcomes of autologous MSC therapies in randomized controlled trials, but their quality of evidence was also low. About risks, no serious adverse events were reported in patients who received MSC therapy at a maximum of 4 years follow-up. At 7 years follow-up, Park^3^ reported no cases of osteogenesis or tumorigenesis, while improved clinical outcomes were stable.

The concept of repeated cell therapy was initially proposed on the basis of experimental studies in rodents with use in cardiac applications with positive results^5^. In the same field (cardiology), the idea of repeated doses of MSCs was mentioned as a major paradigm shift that may fundamentally change the entire field of cell therapy^6,7^.

However, this concept has not been studied in MSK applications in detail. Nevertheless, as OA is a degenerative and progressive disease, it looks reasonable to expect that a single cycle of cell administration would not be sufficient to correct a chronic process. The traditional view of the mechanisms of action of CBT assumed that transplanted cells would engraft, multiply, and differentiate into new mature cells; therefore, it was thought that adequate regeneration could be achieved simply by administering one (sufficiently large) dose of cells. However, as mentioned in several clinical studies, we now know that there is only a minimal, if any, long-term engraftment, especially when cells are injected intra-articularly regardless of the cell type used^8,9^. The main mechanism of action attributed to MSCs in the treatment of knee OA is related to their paracrine signaling activity through which they interact with resident progenitor cells and initiate reparative processes that also involve a modulation in inflammation. Clinical evidence shows a perdurable effect (up to 24 months) even for a single dose of intra-articularly injected CBT, regardless of the tissue origin and the preparation method^10–14^. When this new balance is compromised by disease progression, upon the re-emergence of symptoms a new cycle of CBT may be required, similar to conventional corticosteroids, hyaluronic acid, and PRP^15^.

No severe adverse events have been described for multiple or repeated administrations of CBT. However, the current literature does not support with strong clinical evidence a repetitive CBT after a previous successful treatment at the re-emergence of symptoms.

In a RCT from 2019, Freitag et al. compared the 12 months-results of a single knee injection of 100X10^6^ autologous expanded AT-MSCs in OA patients with the results of two injections of the same amount of cells 6 months apart. Both groups of patients were associated with successful disease modifications. However, the data showed that two intra-articular injections of AT-MSCs achieved more consistent OA stabilization than a single one. A third treatment group receiving five injections of 40×10^6^ADMSCs at baseline, 1, 2, 3 and 6 months, respectively, was intended but ceased due to observed and reproducible moderate adverse events in a concurrently run study with the same treatment protocol (documented as increasing self-limiting pain with sequential injections at monthly intervals). The authors concluded that intra-articular autologous AT-MSC therapy was shown to be safe and related to no serious adverse events at 12 months follow-up^16^. Although it is not specified in the manuscript, an injection performed 6 months after the previous one could make this treatment to be considered as a second therapeutic cycle rather than one consisting of multiple injections.

Another study^17^ described the use of autologous AT-MSCs on 18 patients with two injections (baseline and week 6) using three different doses (10, 20, or 50 x 10^6^ cells). Fourteen of these patients voluntarily chose to receive a third injection after 12 months from the first one. After the first two injections, a substantial improvement in the function, pain and quality of life was reported at 12 weeks, whereas a decreasing tendency along time was observed. The third injection generated another increase in the improvement rate, especially in the low- and middle-dose groups, highlighting a time- and dose-dependent effect. During the study period, no death or SAEs was reported, with no significant difference among the three dose-groups.

*Allogeneic CBT*

Multipotent stem cells have in the recent years been shown to have heterogenous major histocompatibility complex (MHC) class I and II (Human leukocyte antigen (HLA)) to various degrees on their cell surface^18^. This potentially allows for T-cell recognition and initiation of an unwanted immune response when applying mismatched allogeneic stem cells – especially if applied repeatedly.

A single injection of allogeneic stem cells has in several osteoarthritis studies shown clinical improvement without severe effects^19–21^. These findings are confirmed in a recent review by Coop et al.^22^ showing that 5/6 (83%) of clinical OA studies using allogeneic expanded MSCs resulted in significant PROM-improvements. This number was 8/9 (89%) when using autologous MSCs. Nevertheless, a recent study by Mautner et al.^23^ showed a statistically increase in joint swelling when using allogeneic UC-MSCs (24.1%) compared to corticosteroids (17.4%), whereas no difference was seen in joint effusion when comparing a corticosteroid injection to autologous BMAC or SVF.

When it comes to multiple injection, allogeneic expanded umbilical cord MSC were used in a controlled randomized trial where 2 injections administered 6 months apart provided better results than a single dose, thus further supporting the assumption that multiple injections over time may contribute to increase the clinical response. Compared to the control group (hyaluronic acid), more mild to moderate symptomatic knee effusion was present in the allogeneic UC-MSC groups (22% vs. 33%) one week after the first treatment although not statistically significant. The second allogeneic UC-MSC treatment resulted in symptomatic knee effusion in 44% of the patients^24^ compared to 37.5% in patients who had two HA injections, again non significant. Similarly, Ao et al.^25^ showed that repeated intra-articular injection of allogeneic umbilical cord MSCs were safe in treating OA, as no severe adverse events occurred during the 3 months follow-up. In this study, 14 patients received 4 intra-articular injections once a week and although adverse reactions including joint pain, swelling, numbness and stiffness emerged during the study, all of them were transient and did not permanently harm the patient. No control group receiving only one or fewer injections, was included in the study.

**References**

1. Al-Najar M, Khalil H, Al-Ajlouni J, et al. Intra-articular injection of expanded autologous bone marrow mesenchymal cells in moderate and severe knee osteoarthritis is safe: a phase I/II study. *J Orthop Surg Res*. 2017;12(1):190. doi:10.1186/s13018-017-0689-6

**Study type: Prospective uncontrolled clinical trial; Level of evidence 3.**

2. Emadedin M, Labibzadeh N, Liastani MG, et al. Intra-articular implantation of autologous bone marrow-derived mesenchymal stromal cells to treat knee osteoarthritis: a randomized, triple-blind, placebo-controlled phase 1/2 clinical trial. *Cytotherapy*. 2018;20(10):1238-1246. doi:10.1016/j.jcyt.2018.08.005

**Study type: RCT; Level of evidence 1.**

3. Park YB, Ha CW, Lee CH, Yoon YC, Park YG. Cartilage Regeneration in Osteoarthritic Patients by a Composite of Allogeneic Umbilical Cord Blood-Derived Mesenchymal Stem Cells and Hyaluronate Hydrogel: Results from a Clinical Trial for Safety and Proof-of-Concept with 7 Years of Extended Follow-Up. *Stem Cells Transl Med*. 2017;6(2):613-621. doi:10.5966/sctm.2016-0157

**Study type: Clinical trial; Level of evidence 3.**

4. Wiggers TG, Winters M, Van den Boom NA, Haisma HJ, Moen MH. Autologous stem cell therapy in knee osteoarthritis: a systematic review of randomised controlled trials. *Br J Sports Med*. 2021;55(20):1161-1169. doi:10.1136/bjsports-2020-103671

**Study type: Systematic Review; Level of evidence 2.**

5. Tokita Y, Tang XL, Li Q, et al. Repeated Administrations of Cardiac Progenitor Cells Are Markedly More Effective Than a Single Administration: A New Paradigm in Cell Therapy. *Circ Res*. 2016;119(5):635-651. doi:10.1161/CIRCRESAHA.116.308937

**Study type: *in vivo* study.**

6. Bolli R. Repeated Cell Therapy: A Paradigm Shift Whose Time Has Come. *Circ Res*. 2017;120(7):1072-1074. doi:10.1161/CIRCRESAHA.117.310710

**Study type: Narrative Review.**

7. Guo Y, Wysoczynski M, Nong Y, et al. Repeated doses of cardiac mesenchymal cells are therapeutically superior to a single dose in mice with old myocardial infarction. *Basic Res Cardiol*. 2017;112(2):18. doi:10.1007/s00395-017-0606-5

**Study type: *in vivo* study.**

8. Im GI. Perspective on Intra-articular Injection Cell Therapy for Osteoarthritis Treatment. *Tissue Eng Regen Med*. 2019;16(4):357-363. doi:10.1007/s13770-018-00176-6

**Study type: Expert opinion; Level of evidence 4.**

9. Yang C, Wang G, Ma F, et al. Repeated injections of human umbilical cord blood-derived mesenchymal stem cells significantly promotes functional recovery in rabbits with spinal cord injury of two noncontinuous segments. *Stem Cell Res Ther*. 2018;9(1):136. doi:10.1186/s13287-018-0879-0

**Study type: *in vivo* study.**

10. Zhang S, Xu H, He B, et al. Mid-term prognosis of the stromal vascular fraction for knee osteoarthritis: a minimum 5-year follow-up study. *Stem Cell Res Ther*. 2022;13(1):105. doi:10.1186/s13287-022-02788-1

**Study type: RCT; Level of evidence 1.**

11. Zaffagnini S, Andriolo L, Boffa A, et al. Microfragmented Adipose Tissue Versus Platelet-Rich Plasma for the Treatment of Knee Osteoarthritis: A Prospective Randomized Controlled Trial at 2-Year Follow-up. *Am J Sports Med*. 2022;50(11):2881-2892. doi:10.1177/03635465221115821

**Study type: RCT; Level of evidence 1.**

12. Boffa A, Di Martino A, Andriolo L, et al. Bone marrow aspirate concentrate injections provide similar results versus viscosupplementation up to 24 months of follow-up in patients with symptomatic knee osteoarthritis. A randomized controlled trial. *Knee Surg Sports Traumatol Arthrosc*. 2022;30(12):3958-3967. doi:10.1007/s00167-021-06793-4

**Study type: RCT; Level of evidence 2.**

13. Khoury MA, Chamari K, Tabben M, et al. Knee osteoarthritis: clinical and MRI outcomes after multiple intra-articular injections with expanded autologous adipose-derived stromal cells or platelet-rich plasma. *Cartilage*. 2023;14(4):433-444. doi:10.1177/19476035231166127

**Study type: RCT; Level of evidence 2.**

14. Lamo-Espinosa JM, Mora G, Blanco JF, et al. Intra-articular injection of two different doses of autologous bone marrow mesenchymal stem cells versus hyaluronic acid in the treatment of knee osteoarthritis: long-term follow up of a multicenter randomized controlled clinical trial (phase I/II). *J Transl Med*. 2018;16(1):213. doi:10.1186/s12967-018-1591-7

**Study type: RCT; Level of evidence 1.**

15. Emadedin M, Aghdami N, Taghiyar L, et al. Intra-articular injection of autologous mesenchymal stem cells in six patients with knee osteoarthritis. *Arch Iran Med*. 2012;15(7):422-428.

**Study type: Case Series; Level of evidence 4.**

16. Freitag J, Bates D, Wickham J, et al. Adipose-derived mesenchymal stem cell therapy in the treatment of knee osteoarthritis: a randomized controlled trial. *Regen Med*. 2019;14(3):213-230. doi:10.2217/rme-2018-0161

**Study type: RCT; Level of evidence 2.**

17. Song Y, Du H, Dai C, et al. Human adipose-derived mesenchymal stem cells for osteoarthritis: a pilot study with long-term follow-up and repeated injections. *Regen Med*. 2018;13(3):295-307. doi:10.2217/rme-2017-0152

**Study type: Clinical Trial; Level of evidence 3.**

18. Schnabel LV, Pezzanite LM, Antczak DF, Felippe MJB, Fortier LA. Equine bone marrow-derived mesenchymal stromal cells are heterogeneous in MHC class II expression and capable of inciting an immune response in vitro. *Stem Cell Res Ther*. 2014;5(1):13. doi:10.1186/scrt402

**Study type: *In vitro* study.**

19. Vega A, Martín-Ferrero MA, Del Canto F, et al. Treatment of Knee Osteoarthritis With Allogeneic Bone Marrow Mesenchymal Stem Cells: A Randomized Controlled Trial. *Transplantation*. 2015;99(8):1681-1690. doi:10.1097/TP.0000000000000678

**Study type: RCT; Level of evidence 2.**

20. Song JS, Hong KT, Kim NM, et al. Implantation of allogenic umbilical cord blood-derived mesenchymal stem cells improves knee osteoarthritis outcomes: Two-year follow-up. *Regen Ther*. 2020;14:32-39. doi:10.1016/j.reth.2019.10.003

**Study type: Case series; Level of evidence 4.**

21. de Windt TS, Vonk LA, Slaper-Cortenbach ICM, et al. Allogeneic mesenchymal stem cells stimulate cartilage regeneration and are safe for single-stage cartilage repair in humans upon mixture with recycled autologous chondrons. *Stem Cells*. 2017;35(1):256-264. doi:10.1002/stem.2475

**Study type: Clinical Trial; Level of evidence 3.**

22. Copp G, Robb KP, Viswanathan S. Culture-expanded mesenchymal stromal cell therapy: does it work in knee osteoarthritis? A pathway to clinical success. *Cell Mol Immunol*. 2023;20(6):626-650. doi:10.1038/s41423-023-01020-1

**Study type: Narrative review; Level of evidence 4.**

23. Mautner K, Gottschalk M, Boden SD, et al. Cell-based versus corticosteroid injections for knee pain in osteoarthritis: a randomized phase 3 trial. *Nat Med*. 2023;29(12):3120-3126. doi:10.1038/s41591-023-02632-w

**Study type: RCT; Level of evidence 1.**

24. Matas J, Orrego M, Amenabar D, et al. Umbilical Cord-Derived Mesenchymal Stromal Cells (MSCs) for Knee Osteoarthritis: Repeated MSC Dosing Is Superior to a Single MSC Dose and to Hyaluronic Acid in a Controlled Randomized Phase I/II Trial. *Stem Cells Transl Med*. 2019;8(3):215-224. doi:10.1002/sctm.18-0053

**Study type: RCT; Level of evidence 2.**

25. Ao Y, Duan J, Xiong N, et al. Repeated intra-articular injections of umbilical cord-derived mesenchymal stem cells for knee osteoarthritis: a phase I, single-arm study. *BMC Musculoskelet Disord*. 2023;24(1):488. doi:10.1186/s12891-023-06555-y

**Study type: Case series; Level of evidence 4.**

**QUESTION 9**

**Are there advantages of CBT use in comparison to Corticosteroids (CS) for treating knee OA?**

**Statement**

**Although the literature is sparse with regards to direct comparisons between CBT and corticosteroids (CS), current available evidence does not show the clinical superiority of CBT compared to CS**. However, CS injections have been shown to have detrimental effects on chondrocytes and can lead to accelerated cartilage degeneration, especially with multiple/repeated injections. Although corticosteroids are strong anti-inflammatory agents and can provide short-term relief in knee OA (mainly less than 3 months), CBT injections have been shown to have the potential for a longer effect in comparison to the shorter-term effect of CS injections. CBT also seems to provide a safer use profile with less potentially related complications compared to CS, especially when considering the potential need for repeated injections in knee OA patients, more so in younger patients. Therefore, **the consensus group considers CBT injections to be a non-chondro-toxic and effective treatment option, with potentially expected longer-term clinical improvements compared to CS injections.**

Grade of recommendation: D

Rating Score: Mean 8.3±0.8; Median 8 (6-9) Appropriate with relative agreement

*This statement is valid for both POC- and Expanded-CBT*

**Literature summary**

Only a few studies were found making a direct comparison between CBT and Corticosteroids^1,2^. Bastos et al.^1^ in their RCT showed that patient with KL 1-4 knee OA treated with culture expanded BM- SCs or BM-MSCs + PRP had higher percentage of improvement in most KOOS domains and global score compared to the corticosteroid group at 12 months. A more recent and larger 4-arm RCT^2^ comparing SVF, BMAC, allogeneic umbilical cord-MSC and corticosteroids reported that at 1 year post injection, none of the three orthobiologic injections was superior to another, or to the CSI control. In addition, none of the four groups showed a significant change in magnetic resonance imaging osteoarthritis score compared to baseline. However, with the exception of expanded umbilical cord cells, the other orthobiologics used for this study had no previous literature demonstrating their efficacy.

Moreover, this RCT reports for the first time a long-lasting duration of CS effects up to one year without any sign of decrease along time. This is in contrast with the existing literature, including a recent network meta-analysis^3^ showing that corticosteroids had statistical significant improvement in VAS score at 6 weeks, but not at 3 months, 6 months, and 12 months when treating knee OA. No improvement was seen in WOMAC score at any time point^3^. Similarly, the latest Cochrane review^4^ reports that whether there are clinically important benefits of intra‐articular corticosteroids after one to six weeks remains unclear in view of the overall quality of the evidence, considerable heterogeneity between trials, and evidence of small‐study effects. A single trial included in this review described adequate measures to minimize biases and did not find any benefit of intra‐articular corticosteroids^4^. Likewise, a recent review concludes that recurrent intra articular injections with corticosteroids are shown to provide inferior (or non-superior) symptom relief compared with other injectables (including placebo) at 3 months and beyond^5^.

There is a lack of data on the long-term effects of intra articular injections with Corticosteroids on articular cartilage and its potential relationship to adverse joint effects. However, in some in vivo studies, corticosteroids have been cytotoxic to articular cartilage. In a 2-year randomized controlled trial with IACS injections every 3 months, knees with intra-articular triamcinolone injections experienced greater cartilage loss than knees receiving saline injections^6^.

Back to the comparison between CBT and CS, the network meta-analysis previously mentioned^3^ includes 79 RCTs (8761) evaluating intra-articular injectables in Knee OA at 1, 3, 6 and 12 months. Beyond others, the meta-analysis evaluated CS, BMA/BMAC, MSC, SVF, and HA injections. At 4-6 weeks and 3 months of follow-up, the treatment with the highest P-Score for WOMAC score was HA + CS. At 6-months follow-up, the treatment with the highest P-Score for WOMAC score was PRP. At all post-injection time points, the treatment with the highest P-Score for VAS score and Womac score at 12 Months was SVF. The Authors concluded that current evidence shows that SVF injections result in the greatest improvement in pain and functional outcomes in patients with knee OA at up to 1 year of follow-up. However, as no direct comparison is available the conclusion of this meta-analysis is at high risk of bias.

**References**

1. Bastos R, Mathias M, Andrade R, et al. Intra-articular injection of culture-expanded mesenchymal stem cells with or without addition of platelet-rich plasma is effective in decreasing pain and symptoms in knee osteoarthritis: a controlled, double-blind clinical trial. *Knee Surg Sports Traumatol Arthrosc*. 2020;28(6):1989-1999. doi:10.1007/s00167-019-05732-8

**Study type: RCT; Level of evidence 2.**

2. Mautner K, Gottschalk M, Boden SD, et al. Cell-based versus corticosteroid injections for knee pain in osteoarthritis: a randomized phase 3 trial. *Nat Med*. 2023;29(12):3120-3126. doi:10.1038/s41591-023-02632-w

**Study type: RCT; Level of evidence 1.**

3. Anil U, Markus DH, Hurley ET, et al. The efficacy of intra-articular injections in the treatment of knee osteoarthritis: A network meta-analysis of randomized controlled trials. *The Knee*. 2021;32:173-182. doi:10.1016/j.knee.2021.08.008

**Study type: Network meta-analysis; Level of evidence 2.**

4. Jüni P, Hari R, Rutjes AW, et al. Intra-articular corticosteroid for knee osteoarthritis. Cochrane Musculoskeletal Group, ed. *Cochrane Database of Systematic Reviews*. 2015;2015(11). doi:10.1002/14651858.CD005328.pub3

**Study type: Meta-analysis; Level of evidence 2.**

5. Donovan RL, Edwards TA, Judge A, Blom AW, Kunutsor SK, Whitehouse MR. Effects of recurrent intra-articular corticosteroid injections for osteoarthritis at 3 months and beyond: a systematic review and meta-analysis in comparison to other injectables. *Osteoarthritis and Cartilage*. 2022;30(12):1658-1669. doi:10.1016/j.joca.2022.07.011

**Study type: RCT; Level of evidence 2.**

6. Freitag J, Bates D, Wickham J, et al. Adipose-derived mesenchymal stem cell therapy in the treatment of knee osteoarthritis: a randomized controlled trial. *Regenerative Medicine*. 2019;14(3):213-230. doi:10.2217/rme-2018-0161

**Study type: RCT; Level of evidence 2.**

**QUESTION 10**

**Are there advantages of CBT use in comparison to hyaluronic acid (HA) for treating knee OA?**

**Statement**

**Are there advantages of CBT use in comparison to (HA) for treating knee OA?**

Several high-level studies, as well as meta-analyses, exist comparing the effectiveness of CBT to hyaluronic acid (HA) for knee OA, with the majority favoring CBT in terms of overall clinical improvement and a longer-lasting effect documented to last up to 12 months.

**Based on current available evidence, the consensus group acknowledges that CBT seems to have superiority over HA for knee OA due to overall clinical improvement and expected longer-lasting effects,** whilst also acknowledging that there are different formulations of the products that may introduce some bias in the conclusions of meta-analyses.

Grade of recommendation: B

Rating Score: Mean 8.3±0.8; Median 8 (6-9) Appropriate with relative agreement

However, due to the more invasive and complex preparation process of CBT, **the consensus group recommends that its use should be reserved as a second-line injectable treatment option.**

Grade of recommendation: D

Rating Score: Mean 8.3±1.0; Median 9 (5-9) Appropriate with relative agreement

*These statements are valid for both POC- and Expanded-CBT*

**Literature summary**

Three meta-analyses^1–3^ have been recently published comparing several treatment options for knee osteoarthritis (OA). Among these meta-analyses, Anil et al.^1^ performed a network meta-analysis of 79 level 1 and 2 studies to ascertain whether there is a superior intra-articular injection treatment for knee OA, from ozone to botulinum toxin, to HA, CS and cell-based therapies. Among these different therapies, the current review focused on the comparison of HA with different cell-based therapies (SVF, BMAC, and expanded MSC from adipose tissue, peripheral blood or fetal annexes), in order to identify which therapy is better for the management of knee OA. When gathering and crossing data from these meta-analyses, studies provided analyses on effect of these CBT versus HA, for a total of 422 and 304 patients, respectively, whose results are defined below:

*Adipose-Derived Stromal Vascular Fraction (SVF):* the two RCTs^4,5^ specifically comparing SVF and HA included 66 patients in each group, with Kellgren-Lawrence grades of knee OA between 2 and 3. Both studies came to identical conclusions: SVF significantly improved clinical and MRI scores at 6 and 12 months compared with HA.

*Bone Marrow Aspirate Concentrate (BMAC):* three RCTs^6–8^ specifically analyzed BMAC in comparison with HA in knee OA, including 195 and 114 patients respectively. Kellgren-Lawrence grades of knee OA were between 1-4^6^, 2-3^7^ and 3-4^8^. Both meta-analyses^2,3,9^ that examined BMAC ^2^reported better results with BMAC compared with HA injections for patients with knee OA with statistically significant difference concerning the WOMAC, VAS, and Subjective IKDC scores, although BMAC administration techniques and strategies were not identical across all studies.

*Expanded Mesenchymal Stem Cells (MSCs)*: eight studies were focused on the use of expanded MSCs vs HA in knee OA, and respectively include 211 and 174 patients. Three studies used expanded MSC from bone marrow, autologous^10^, allogeneic^11^ or in combination with HA^12^ Two studies^13,14^ used expanded MSC from human umbilical cord (one reported only in Chinese^13^), two others^15,16^ used peripheral blood stem cells, and the last used adipose-derived stem cells^17^. When available, Kellgren-Lawrence grades of knee OA were 1-3,^17^ 2-3,^14,15^ 2-4,^10,11^ and 3-4^13^. Whatever the tissue of origin of the MSC, human umbilical cord, adipose tissue, or peripheral blood, Jiang et al.^9^ conclude that several symptomatic scores improved significantly at 6 months and most of the scales improved at 12 months. Of note, one study assessed the effect of MSC in combination with HA vs HA alone.

*Amniotic Suspension Allograft (ASA):* only one randomized controlled trial^18^ assessed the effect of ASA vs HA in knee OA with 66 and 64 patients respectively. Kellgren-Lawrence grades of knee OA were 2 or 3. Gomoll et al. reported clinically meaningful improved KOOS and VAS with ASA over HA out to 12 months postinjection, however there were no differences between groups for radiographic measures.

*Microfragmented Adipose Tissue (MFAT):* To date, no study has compared MFAT with HA. In another recent systematic review and meta-analysis of 27 Level 1 studies, Belk et al^2^. systematically reviewed the literature to compare the efficacy and safety of PRP, BMAC, HA injections for the treatment of knee OA. The 27 studies included 1,042 patients undergoing intra-articular injection(s) with PRP (mean age 57.7 years, mean follow-up 13.5 months), 226 patients with BMAC (mean age 57.0 years, mean follow-up 17.5 months), and 1,128 patients with HA (mean age 59.0 years, mean follow-up 14.4 months). Non-network meta-analyses demonstrated significantly better postinjection WOMAC, VAS and Subjective IKDC scores in patients who received PRP compared with patients who received HA. Similarly, network meta-analyses demonstrated significantly better postinjection WOMAC, VAS and Subjective IKDC scores in patients who received BMAC compared with patients who received HA. Similar results were found in the level 1 systematic review and metanalysis of Kim et al.^3^, in this case comparing HA with adipose-derived cell-based products (AT-MSCs or SVF). The literature search identified 5 level 1 RCTs that evaluated the efficacy and safety of intra-articular injections of autologous AT-MSCs or SVFs without adjuvant treatments compared with placebo or hyaluronic acid in patients with knee OA. Based on the meta-analysis, AT-MSCs or SVFs showed significantly better pain relief at 6 months and 12 months and functional improvement at 6 months and 12 months without a difference in procedure-related knee pain or swelling compared with controls. No serious AEs associated with AT-MSCs or SVFs were reported. Subgroup analyses showed similar efficacy between AT-MSC and SVF treatments.

**References**

1. Anil U, Markus DH, Hurley ET, et al. The efficacy of intra-articular injections in the treatment of knee osteoarthritis: A network meta-analysis of randomized controlled trials. *The Knee*. 2021;32:173-182. doi:10.1016/j.knee.2021.08.008

**Study type: Network meta-analysis; Level of evidence 2.**

2. Belk JW, Lim JJ, Keeter C, et al. Patients With Knee Osteoarthritis Who Receive Platelet-Rich Plasma or Bone Marrow Aspirate Concentrate Injections Have Better Outcomes Than Patients Who Receive Hyaluronic Acid: Systematic Review and Meta-analysis. *Arthrosc J Arthrosc Relat Surg*. 2023;39(7):1714-1734. doi:10.1016/j.arthro.2023.03.001

**Study type: Systematic Review and Meta-analysis; Level of evidence 1.**

3. Kim KI, Kim MS, Kim JH. Intra-articular Injection of Autologous Adipose-Derived Stem Cells or Stromal Vascular Fractions: Are They Effective for Patients With Knee Osteoarthritis? A Systematic Review With Meta-analysis of Randomized Controlled Trials. *Am J Sports Med*. 2023;51(3):837-848. doi:10.1177/03635465211053893

**Study type: Meta-analysis; Level of evidence 1.**

4. Hong Z, Chen J, Zhang S, et al. Intra-articular injection of autologous adipose-derived stromal vascular fractions for knee osteoarthritis: a double-blind randomized self-controlled trial. *Int Orthop*. 2019;43(5):1123-1134. doi:10.1007/s00264-018-4099-0

**Study type: RCT; Level of evidence 1.**

5. Zhang Y, Bi Q, Luo J, Tong Y, Yu T, Zhang Q. The Effect of Autologous Adipose-Derived Stromal Vascular Fractions on Cartilage Regeneration Was Quantitatively Evaluated Based on the 3D-FS-SPGR Sequence: A Clinical Trial Study. Du J, ed. *BioMed Res Int*. 2022;2022:1-17. doi:10.1155/2022/2777568

**Study type: RCT; Level of evidence 1.**

6. Boffa A, Di Martino A, Andriolo L, et al. Bone marrow aspirate concentrate injections provide similar results versus viscosupplementation up to 24 months of follow-up in patients with symptomatic knee osteoarthritis. A randomized controlled trial. *Knee Surg Sports Traumatol Arthrosc*. 2022;30(12):3958-3967. doi:10.1007/s00167-021-06793-4

**Study type: RCT; Level of evidence 2.**

7. Goncars V, Jakobsons E, Blums K, et al. The comparison of knee osteoarthritis treatment with single-dose bone marrow-derived mononuclear cells vs. hyaluronic acid injections. *Medicina (Mex)*. 2017;53(2):101-108. doi:10.1016/j.medici.2017.02.002

**Study type: RCT; Level of evidence 2.**

8. Dulic O, Rasovic P, Lalic I, et al. Bone Marrow Aspirate Concentrate versus Platelet Rich Plasma or Hyaluronic Acid for the Treatment of Knee Osteoarthritis. *Medicina (Mex)*. 2021;57(11):1193. doi:10.3390/medicina57111193

**Study type: RCT; Level of evidence 2.**

9. Jiang P, Mao L, Qiao L, Lei X, Zheng Q, Li D. Efficacy and safety of mesenchymal stem cell injections for patients with osteoarthritis: a meta-analysis and review of RCTs. *Arch Orthop Trauma Surg*. 2021;141(7):1241-1251. doi:10.1007/s00402-020-03703-0

**Study type: Meta-analysis; Level of evidence 1.**

10. Lamo-Espinosa JM, Mora G, Blanco JF, et al. Intra-articular injection of two different doses of autologous bone marrow mesenchymal stem cells versus hyaluronic acid in the treatment of knee osteoarthritis: long-term follow up of a multicenter randomized controlled clinical trial (phase I/II). *J Transl Med*. 2018;16(1):213. doi:10.1186/s12967-018-1591-7

**Study type: RCT; Level of evidence 1.**

11. Vega A, Martín-Ferrero MA, Del Canto F, et al. Treatment of Knee Osteoarthritis With Allogeneic Bone Marrow Mesenchymal Stem Cells: A Randomized Controlled Trial. *Transplantation*. 2015;99(8):1681-1690. doi:10.1097/TP.0000000000000678

**Study type: RCT; Level of evidence 2.**

12. Wong KL, Lee KBL, Tai BC, Law P, Lee EH, Hui JHP. Injectable Cultured Bone Marrow–Derived Mesenchymal Stem Cells in Varus Knees With Cartilage Defects Undergoing High Tibial Osteotomy: A Prospective, Randomized Controlled Clinical Trial With 2 Years’ Follow-up. *Arthrosc J Arthrosc Relat Surg*. 2013;29(12):2020-2028. doi:10.1016/j.arthro.2013.09.074

**Study type: RCT; Level of evidence 2.**

13. Wang Y, Jin W, Liu H, Cui Y, Mao Q, Fei Z, Xiang C. [Curative effect of human umbilical cord mesenchymal stem cells by intra-articular injection for degenerative knee osteoarthritis]. *Zhongguo Xiu Fu Chong Jian Wai Ke Za Zhi*. 2016; 30(12):1472-1477. Chinese. doi: 10.7507/1002-1892.20160305.

**Study type: RCT; Level of evidence 2.**

14. Matas J, Orrego M, Amenabar D, et al. Umbilical Cord-Derived Mesenchymal Stromal Cells (MSCs) for Knee Osteoarthritis: Repeated MSC Dosing Is Superior to a Single MSC Dose and to Hyaluronic Acid in a Controlled Randomized Phase I/II Trial. *Stem Cells Transl Med*. 2019;8(3):215-224. doi:10.1002/sctm.18-0053

**Study type: RCT; Level of evidence 2.**

15. Turajane T, Chaveewanakorn U, Fongsarun W, Aojanepong J, Papadopoulos KI. Avoidance of Total Knee Arthroplasty in Early Osteoarthritis of the Knee with Intra-Articular Implantation of Autologous Activated Peripheral Blood Stem Cells versus Hyaluronic Acid: A Randomized Controlled Trial with Differential Effects of Growth Factor Addition. *Stem Cells Int*. 2017;2017:1-10. doi:10.1155/2017/8925132

**Study type: RCT; Level of evidence 2.**

16. Saw KY, Anz A, Siew-Yoke Jee C, et al. Articular Cartilage Regeneration With Autologous Peripheral Blood Stem Cells Versus Hyaluronic Acid: A Randomized Controlled Trial. *Arthrosc J Arthrosc Relat Surg*. 2013;29(4):684-694. doi:10.1016/j.arthro.2012.12.008

**Study type: RCT; Level of evidence 2.**

17. Lu L, Dai C, Zhang Z, et al. Treatment of knee osteoarthritis with intra-articular injection of autologous adipose-derived mesenchymal progenitor cells: a prospective, randomized, double-blind, active-controlled, phase IIb clinical trial. *Stem Cell Res Ther*. 2019;10(1):143. doi:10.1186/s13287-019-1248-3

**Study type: RCT; Level of evidence 1.**

18. Gomoll AH, Farr J, Cole BJ, et al. Safety and Efficacy of an Amniotic Suspension Allograft Injection Over 12 Months in a Single-Blinded, Randomized Controlled Trial for Symptomatic Osteoarthritis of the Knee. *Arthrosc J Arthrosc Relat Surg*. 2021;37(7):2246-2257. doi:10.1016/j.arthro.2021.02.044

**Study type: RCT; Level of evidence 1.**

**QUESTION 11**

**Are there advantages of CBT use in comparison to platelet rich plasma (PRP) for treating knee OA?**

**Statement**

Current literature with regards to the advantage or superiority of CBT compared to platelet rich plasma (PRP) is limited and inconclusive, with few studies performed with direct comparisons between CBT and PRP. Therefore, based on current evidence, **the consensus group does not acknowledge superiority or clear advantages of CBT over PRP for knee OA.**

Grade of recommendation: C

Rating Score: Mean 8.5±0.9; Median 9 (5-9) Appropriate with relative agreement

Moreover, considering the relatively invasive and more complex nature of the preparation procedure of CBT compared to PRP, **the consensus group recommends that PRP should be used as a first-line orthobiologic injectable treatment option in knee OA, while CBT could be considered as a second-line orthobiologic treatment option.**

Grade of recommendation: D

Rating Score: Mean 8.6±0.6; Median 9 (7-9) Strong agreement

*These statements are valid for both POC- and Expanded-CBT*

**Literature summary**

Several randomized trials and meta-analyses support the use of platelet rich plasma (PRP) for knee osteoarthritis (OA) compared with placebo and standard-of-care treatment options as steroids and viscosupplemention^1,2^.

A recent level 1 and 2 systematic review/network metanalysis^3^ compared the efficacy of nonsurgical interventions in knee OA. It included 19 RCTs studying 16 different interventions: injections of PRP were assessed in 8 studies, and cell based therapies (CBT) in 5 trials divided as follows: expanded MSCs (2 studies, adipose^4^ - or umbilical cord-derived^5^) , stromal vascular fraction (SVF^6^), bone marrow aspirate concentrate (BMAC ^7^) and amniotic suspension allograft^8^. Expanded MSCs and PRP showed the most consistent results and were associated with improvement in pain and articular function in the long-term, with a significantly greater WOMAC decrease at 12 months for MSCs and PRP vs other treatments. The study reported that MSC-products had the higher probability to be the best treatment, while PRP ranked as the second-best. However, the studies analyzed contain limitations such as heterogeneity for PRP strategy and protocols applied, short follow-up and in general limited quality of the studies included. Therefore, in order to confirm the clear superiority of one treatment over the other more high-quality RCTs would be required.

Another systematic meta-analysis of Level 1 and 2 studies^9^ evaluated the clinical efficacy and adverse events related to different treatment of knee OA, including PRP (24 studies, 12 studies leukocyte poor (LP)-PRP, 11 studies leukocyte rich (LR)-PRP, 1 study LP-PRP vs LR-PRP) and CBT (3 studies BM-MSC, 3 studies AT-MSC) and hyaluronic acid (HA) (13 studies). It was found that PRP, AT-MSCs and bone marrow BM-MSCs) were associated with better outcomes without clear significant superiority. To note, at 6 months, VAS scores and WOMAC pain subscores suggested AT-MSCs as the best treatment option for pain relief. According to WOMAC scores and subjective International Knee Documentation Committee (IKDC) scores, PRP seemed to be the most effective treatment for functional improvement. At 12 months, only AT-MSCs were associated with improved VAS scores compared with the placebo, whereas functional improvement was achieved with PRP. Potentially beneficial effects were observed for BM-MSCs, but other studies including direct head-to-head comparisons are needed to support reliable conclusions. All treatments except for LP-PRP had a higher incidence of treatment-related adverse effects than placebo that consist in mild local reactions, such as arthralgia and swelling which can self-alleviate without additional treatments.

A systematic review^10^ of biologic therapies for knee OA included eighty-two studies with PRP, comprising the majority (51 studies), and a limited number of studies for CBT: BM-MSCs (15 studies), AT-MSCs (11 studies) and hAMSCs (5 studies). The review included also studies that directly compared PRP and CBT treatments: 2 PRP vs BM-MSC^11,12^, 1 PRP vs MFAT^13^, 1 PRP+HA vs AT-MSCs^14^, 1 PRP vs AT-MSCs vs BM-MSCs^15^. No significant differences emerged, probably due to limitations existing in the study: number of available levels I and II studies were limited among non-PRP groups (BM-MSCs, AT-MSCs, and hAM-MSCs); formulations, injection techniques, and reported outcomes varied across all studies making study comparisons challenging.

A prospective study^16^ directly comparing clinical outcomes of knee injections of BMAC, PRP or HA for knee OA suggested a superiority for BMAC treatment up to 12 months in terms of KOOS and VAS scores, but not in terms of WOMAC and IKDC scales. Moreover, an immediate pain relief after BMAC injection was reported. In vitro comparative studies suggest that the superiority of BMAC treatment is due to the higher number of different cells with an active biological role and a higher concentration of bioactive molecules compared to PRP^17,18^. However more randomized controlled trials and high quality comparative studies are necessary to establish a real clinical advantage.

The therapeutic effect of a single dose of PRP was also compared to a single dose injection of MFAT in a RCT in 58 patients with symptomatic knee OA (KL 1-4) who were divided into two groups: 30 patients received a single injection of LR-PRP and another group of 28 patients received MFAT under ultrasound guidance^19^. A single injection of either PRP or MFAT resulted in clinically and statistically significant improvements in pain, mechanical symptoms, functional ability, and quality of life at 6 months post-injection, and again, considering limitations of the study, no difference in treatment efficacy between groups was reported.

Another level 1 randomized controlled trial comparing MFAT and PRP injections in 118 patients with symptomatic knee OA^20^ evaluated before the injection and at 1, 3, 6, 12, and 24 months with the IKDC subjective score, KOOS subscales and VAS for pain, reported significant improvement in terms of clinical and imaging outcomes for up to 24 months in both groups, with no significant differences among them. However, analyzing more severe cases of OA (KL3-4), more patients in the MFAT group reached the minimal clinically important difference for the IKDC score at 6 months compared with the PRP group (75.0% vs 34.6%, respectively; P = .005).

**References**

1. Xue Y, Wang X, Wang X, Huang L, Yao A, Xue Y. A comparative study of the efficacy of intra-articular injection of different drugs in the treatment of mild to moderate knee osteoarthritis: A network meta-analysis. *Medicine (Baltimore)*. 2023;102(12):e33339. doi:10.1097/MD.0000000000033339

**Study type: Network Meta-analysis; Level of evidence 2.**

2. Belk JW, Kraeutler MJ, Houck DA, Goodrich JA, Dragoo JL, McCarty EC. Platelet-Rich Plasma Versus Hyaluronic Acid for Knee Osteoarthritis: A Systematic Review and Meta-analysis of Randomized Controlled Trials. *Am J Sports Med*. 2021;49(1):249-260. doi:10.1177/0363546520909397

**Study type: Systematic review and Meta-analysis; Level of evidence 1.**

3. Naja M, Fernandez De Grado G, Favreau H, et al. Comparative effectiveness of nonsurgical interventions in the treatment of patients with knee osteoarthritis: A PRISMA-compliant systematic review and network meta-analysis. *Medicine (Baltimore)*. 2021;100(49):e28067. doi:10.1097/MD.0000000000028067

**Study type: Systematic review and Network Meta-analysis; Level of evidence 1.**

4. Freitag J, Bates D, Wickham J, et al. Adipose-derived mesenchymal stem cell therapy in the treatment of knee osteoarthritis: a randomized controlled trial. *Regen Med*. 2019;14(3):213-230. doi:10.2217/rme-2018-0161

**Study type: RCT; Level of evidence 2.**

5. Matas J, Orrego M, Amenabar D, et al. Umbilical Cord-Derived Mesenchymal Stromal Cells (MSCs) for Knee Osteoarthritis: Repeated MSC Dosing Is Superior to a Single MSC Dose and to Hyaluronic Acid in a Controlled Randomized Phase I/II Trial. *Stem Cells Transl Med*. 2019;8(3):215-224. doi:10.1002/sctm.18-0053

**Study type: RCT; Level of evidence 2.**

6. Hong Z, Chen J, Zhang S, et al. Intra-articular injection of autologous adipose-derived stromal vascular fractions for knee osteoarthritis: a double-blind randomized self-controlled trial. *Int Orthop*. 2019;43(5):1123-1134. doi:10.1007/s00264-018-4099-0

**Study type: RCT; Level of evidence 1.**

7. Shapiro SA, Kazmerchak SE, Heckman MG, Zubair AC, O’Connor MI. A Prospective, Single-Blind, Placebo-Controlled Trial of Bone Marrow Aspirate Concentrate for Knee Osteoarthritis. *Am J Sports Med*. 2017;45(1):82-90. doi:10.1177/0363546516662455

**Study type: RCT; Level of evidence 2.**

8. Farr J, Gomoll AH, Yanke AB, Strauss EJ, Mowry KC, ASA Study Group. A Randomized Controlled Single-Blind Study Demonstrating Superiority of Amniotic Suspension Allograft Injection Over Hyaluronic Acid and Saline Control for Modification of Knee Osteoarthritis Symptoms. *J Knee Surg*. 2019;32(11):1143-1154. doi:10.1055/s-0039-1696672

**Study type: RCT; Level of evidence 1.**

9. Zhao D, Pan JK, Yang WY, et al. Intra-Articular Injections of Platelet-Rich Plasma, Adipose Mesenchymal Stem Cells, and Bone Marrow Mesenchymal Stem Cells Associated With Better Outcomes Than Hyaluronic Acid and Saline in Knee Osteoarthritis: A Systematic Review and Network Meta-analysis. *Arthroscopy*. 2021;37(7):2298-2314.e10. doi:10.1016/j.arthro.2021.02.045

**Study type: Systematic Review and Network Meta-analysis; Level of evidence 2.**

10. Delanois RE, Sax OC, Chen Z, Cohen JM, Callahan DM, Mont MA. Biologic Therapies for the Treatment of Knee Osteoarthritis: An Updated Systematic Review. *J Arthroplasty*. 2022;37(12):2480-2506. doi:10.1016/j.arth.2022.05.031

**Study type: Systematic review; Level of evidence 2.**

11. Anz AW, Hubbard R, Rendos NK, Everts PA, Andrews JR, Hackel JG. Bone Marrow Aspirate Concentrate Is Equivalent to Platelet-Rich Plasma for the Treatment of Knee Osteoarthritis at 1 Year: A Prospective, Randomized Trial. *Orthop J Sports Med*. 2020;8(2):2325967119900958. doi:10.1177/2325967119900958

**Study type: RCT; Level of evidence 2.**

12. Lamo-Espinosa JM, Blanco JF, Sánchez M, et al. Phase II multicenter randomized controlled clinical trial on the efficacy of intra-articular injection of autologous bone marrow mesenchymal stem cells with platelet rich plasma for the treatment of knee osteoarthritis. *J Transl Med*. 2020;18(1):356. doi:10.1186/s12967-020-02530-6

**Study type: RCT; Level of evidence 2.**

13. Louis ML, Dumonceau RG, Jouve E, et al. Intra-Articular Injection of Autologous Microfat and Platelet-Rich Plasma in the Treatment of Knee Osteoarthritis: A Double-Blind Randomized Comparative Study. *Arthroscopy*. 2021;37(10):3125-3137.e3. doi:10.1016/j.arthro.2021.03.074

**Study type: RCT; Level of evidence 2.**

14. Dallo I, Szwedowski D, Mobasheri A, Irlandini E, Gobbi A. A Prospective Study Comparing Leukocyte-Poor Platelet-Rich Plasma Combined with Hyaluronic Acid and Autologous Microfragmented Adipose Tissue in Patients with Early Knee Osteoarthritis. *Stem Cells Dev*. 2021;30(13):651-659. doi:10.1089/scd.2021.0053

**Study type: RCT; Level of evidence 2.**

15. Estrada E, Décima JL, Rodríguez M, Di Tomaso M, Roberti J. Patient-Reported Outcomes After Platelet-Rich Plasma, Bone Marrow Aspirate, and Adipose-Derived Mesenchymal Stem Cell Injections for Symptomatic Knee Osteoarthritis. *Clin Med Insights Arthritis Musculoskelet Disord*. 2020;13:1179544120931086. doi:10.1177/1179544120931086

**Study type: Comparative study; Level of evidence 2.**

16. Dulic O, Rasovic P, Lalic I, et al. Bone Marrow Aspirate Concentrate versus Platelet Rich Plasma or Hyaluronic Acid for the Treatment of Knee Osteoarthritis. *Medicina (Kaunas)*. 2021;57(11):1193. doi:10.3390/medicina57111193

**Study type: RCT; Level of evidence 2.**

17. Cassano JM, Kennedy JG, Ross KA, Fraser EJ, Goodale MB, Fortier LA. Bone marrow concentrate and platelet-rich plasma differ in cell distribution and interleukin 1 receptor antagonist protein concentration. *Knee Surg Sports Traumatol Arthrosc*. 2018;26(1):333-342. doi:10.1007/s00167-016-3981-9

**Study type: RCT; Level of evidence 2.**

18. Ziegler CG, Van Sloun R, Gonzalez S, et al. Characterization of Growth Factors, Cytokines, and Chemokines in Bone Marrow Concentrate and Platelet-Rich Plasma: A Prospective Analysis. *Am J Sports Med*. 2019;47(9):2174-2187. doi:10.1177/0363546519832003

**Study type: Cross-sectional study; Level of evidence 3.**

19. Baria M, Pedroza A, Kaeding C, et al. Platelet-Rich Plasma Versus Microfragmented Adipose Tissue for Knee Osteoarthritis: A Randomized Controlled Trial. *Orthop J Sports Med*. 2022;10(9):23259671221120678. doi:10.1177/23259671221120678

**Study type: RCT; Level of evidence 2.**

20. Zaffagnini S, Andriolo L, Boffa A, et al. Microfragmented Adipose Tissue Versus Platelet-Rich Plasma for the Treatment of Knee Osteoarthritis: A Prospective Randomized Controlled Trial at 2-Year Follow-up. *Am J Sports Med*. 2022;50(11):2881-2892. doi:10.1177/03635465221115821

**Study type: RCT; Level of evidence 1.**

**QUESTION 12**

**Are there indications for the use of allogeneic cell products for knee OA?**

**Statement**

While several high-quality studies exist evaluating the clinical benefit of allogeneic CBT for knee OA with promising results in terms of clinical benefit, current evidence is still limited due to relatively small sample sizes and heterogeneity in cell preparation and dosing, product characterization, and patient populations. Additionally, the optimal source, dose, and frequency of MSC injections have not been established. Some transient adverse effects have been reported in preclinical studies, especially after repeated injections, as well as in a few clinical studies, often comparable to those observed with the use of autologous cells. **The consensus group cannot suggest allogeneic CBT have an advantage over autologous CBT in terms of clinical benefit, although the lack of donor-site morbidity could play an important role in some subjects. Therefore, the consensus group suggests allogeneic CBT could be considered in patients who cannot have autologous CBT for any reason or in whom autologous CBT is contraindicated.**

*(For contraindications to the use of CBT please refer to Question 6; for effects of repeated CBT injections please refer to question 8)*

Grade of recommendation: C

Rating Score: Mean 7.8±1.1; Median 8 (6-9) Appropriate with relative agreement

*These statements are valid for both POC- and Expanded-CBT*

**Literature summary**

Multipotent stem cells have in the recent years been shown to have heterogenous major histocompatibility complex (MHC) class I and II (Human leukocyte antigen (HLA)) to various degrees on their cell surface. This potentially allows for T-cell recognition and possible initiation of an unwanted immune response when applying mismatched allogeneic stem cells – especially if applied repeatedly. Haplotyping and match of patient and donor is possible prior to treatment, but the process is time-consuming and costly, therefore it is currently not used clinically. Moreover, allogeneic therapy often contains cells from multiple donors to obtain enough cells with high quality, which would make a full match difficult. Cell memory is assessed as a possible reason for the potential foreign body allogeneic reactions. In 2014, Ryan et al^1,2^ showed that differentiation of MSCs to chondrocytes caused a change in their gene expression to the donor they were derived from in an allogeneic rat model. The cells thereby lost their immunosuppressive capacity and were destroyed by an allogeneic-T-cell-initiated immune response. In the same study, differentiated human MSCs also lost their immunosuppressive capacity in vitro, while undifferentiated MSCs caused significantly less immune reaction in the rat model. Some preclinical studies showed immunoreaction to the use of allogeneic MSCs. Eliopoulos et al. showed that allogeneic marrow stromal cells were immune rejected by MHC class I– and class II–mismatched recipient mice^3^, likewise Pezzanite et al.^4^ who reported that equine allogeneic bone marrow-derived mesenchymal stromal cells elicited antibody responses in vivo in horses.

Nevertheless, from a clinical perspective intra-articular injection of allogeneic stem cells has shown in several RCTs clinical improvement without severe adverse effects in knee osteoarthritis^5–12^. A recent review by Copp et al.^13^ showed that 5/6 (83%) of clinical OA studies using allogeneic expanded MSCs resulted in significant PROM-improvements. This number was 8/9 (89%) when using autologous MSCs. Similarly, Song et al.

A recent study by Mautner et al.^14^ also showed a statistically increase in joint swelling when using allogeneic UC-MSCs (24.1%) compared to corticosteroids (17.4%), whereas no difference was seen in joint effusion when comparing a corticosteroid injection to autologous BMAC or SVF.

Some concerns may arise when it comes to multiple injections. Some preclinical studies show that repeated intra-articular injection of allogeneic mesenchymal stem cells caused an adverse response compared to autologous cells in the equine model^15^. However, others found no increase in adverse events after repeated injections of allogeneic cells. Pigott et al.^16^ did not observe a detectable immune response upon re-exposure done in vitro with the recipient PBMCs and same allo MSCs in horses. Similarly, Ardanaz et al.^17^ reported an absence of hypersensitivity response to the second allogeneic BM-MSCs injection in horses, while Barrachina et al.^18^ showed that repeated IA injections of naive and TNF+IFN primed allogeneic BM-MSCs in horses elicited a slight inflammatory reaction after the repeated injection of (only) the primed MSCs. In a follow-up study of the previous one^19^ they found that both naive and primed lead to allo-antibody formation, but the expression on the surface of primed MSCs makes it that they are more easily targeted by the antibodies upon re-exposure. Also, Magri et al.^20^ found no clear benefit of repeated injections in horses, but also no negative effects, as well as Rowland et al.^21^ found a mild local immune response upon second injection in horses when using mismatched allo MSCs.

However, from a clinical perspective no relevant concerns are reported by the two studies available on this topic, a RCT^11^ and a Phase I single arm trial^22^, respectively, both reporting the results of multiple injections of expanded allogeneic UC-MSC. In the first study the second injection was performed 6 months after the first one, while in the second study the patients received three consecutive injections one week apart. None of the studies reported severe adverse events or reaction, where the most common adverse event related to intra-articular injection was acute synovitis, associated to transient transient pain and swelling, as reported in other studies using autologous cells. Interestingly, the study from Matas^11^ showed no significant differences comparing patients who received one single injection of UC-MSCs vs one single inection of HA (33% vs 22%, p=ns), as well as comparing patients who received two injection of UC-MSCs vs two injections of HA (44% vs 37.5%, p=ns).

Interestingly, concerning the mechanism of action of allogeneic cells, a phase I study by Windt et al.^23^ showed that regenerative tissue obtained after treatment of cartilage lesions exclusively consisted of the patient’s own DNA, which highlights that the allogeneic MSCs primary effect is performed by paracrine signaling.

**References**

1. Ryan AE, Lohan P, O’Flynn L, et al. Chondrogenic Differentiation Increases Antidonor Immune Response to Allogeneic Mesenchymal Stem Cell Transplantation. *Molecular Therapy*. 2014;22(3):655-667. doi:10.1038/mt.2013.261

**Study type: *in vivo* study.**

2. Schnabel LV, Pezzanite LM, Antczak DF, Felippe MJB, Fortier LA. Equine bone marrow-derived mesenchymal stromal cells are heterogeneous in MHC class II expression and capable of inciting an immune response in vitro. *Stem Cell Res Ther*. 2014;5(1):13. doi:10.1186/scrt402

**Study type: *in vitro* study.**

3. Eliopoulos N, Stagg J, Lejeune L, Pommey S, Galipeau J. Allogeneic marrow stromal cells are immune rejected by MHC class I– and class II–mismatched recipient mice. *Blood*. 2005;106(13):4057-4065. doi:10.1182/blood-2005-03-1004

**Study type: *in vivo* study.**

4. Pezzanite LM, Fortier LA, Antczak DF, et al. Equine allogeneic bone marrow-derived mesenchymal stromal cells elicit antibody responses in vivo. *Stem Cell Res Ther*. 2015;6(1):54. doi:10.1186/s13287-015-0053-x

**Study type: *in vivo* study.**

5. Chen CF, Hu CC, Wu CT, et al. Treatment of knee osteoarthritis with intra-articular injection of allogeneic adipose-derived stem cells (ADSCs) ELIXCYTE®: a phase I/II, randomized, active-control, single-blind, multiple-center clinical trial. *Stem Cell Res Ther*. 2021;12(1):562. doi:10.1186/s13287-021-02631-z

**Study type: RCT; Level of evidence 1.**

6. Kuah D, Sivell S, Longworth T, et al. Safety, tolerability and efficacy of intra-articular Progenza in knee osteoarthritis: a randomized double-blind placebo-controlled single ascending dose study. *J Transl Med*. 2018;16(1):49. doi:10.1186/s12967-018-1420-z

**Study type: RCT; Level of evidence 1.**

7. Gupta PK, Maheshwari S, Cherian JJ, et al. Efficacy and Safety of Stempeucel in Osteoarthritis of the Knee: A Phase 3 Randomized, Double-Blind, Multicenter, Placebo-Controlled Study. *Am J Sports Med*. 2023;51(9):2254-2266. doi:10.1177/03635465231180323

**Study type: RCT; Level of evidence 1.**

8. Gupta PK, Chullikana A, Rengasamy M, et al. Efficacy and safety of adult human bone marrow-derived, cultured, pooled, allogeneic mesenchymal stromal cells (Stempeucel®): preclinical and clinical trial in osteoarthritis of the knee joint. *Arthritis Res Ther*. 2016;18(1):301. doi:10.1186/s13075-016-1195-7

**Study type: RCT; Level of evidence 1.**

9. Vega A, Martín-Ferrero MA, Del Canto F, et al. Treatment of Knee Osteoarthritis With Allogeneic Bone Marrow Mesenchymal Stem Cells: A Randomized Controlled Trial. *Transplantation*. 2015;99(8):1681-1690. doi:10.1097/TP.0000000000000678

**Study type: RCT; Level of evidence 2.**

10. Khalifeh Soltani S, Forogh B, Ahmadbeigi N, et al. Safety and efficacy of allogenic placental mesenchymal stem cells for treating knee osteoarthritis: a pilot study. *Cytotherapy*. 2019;21(1):54-63. doi:10.1016/j.jcyt.2018.11.003

**Study type: Double-blind placebo controlled RCT; Level of evidence 2.**

11. Matas J, Orrego M, Amenabar D, et al. Umbilical Cord-Derived Mesenchymal Stromal Cells (MSCs) for Knee Osteoarthritis: Repeated MSC Dosing Is Superior to a Single MSC Dose and to Hyaluronic Acid in a Controlled Randomized Phase I/II Trial. *Stem Cells Translational Medicine*. 2019;8(3):215-224. doi:10.1002/sctm.18-0053

**Study type: RCT; Level of evidence 2.**

12. Farr J, Gomoll AH, Yanke AB, Strauss EJ, Mowry KC, ASA Study Group. A Randomized Controlled Single-Blind Study Demonstrating Superiority of Amniotic Suspension Allograft Injection Over Hyaluronic Acid and Saline Control for Modification of Knee Osteoarthritis Symptoms. *J Knee Surg*. 2019;32(11):1143-1154. doi:10.1055/s-0039-1696672

**Study type: RCT; Level of evidence 1.**

13. Copp G, Robb KP, Viswanathan S. Culture-expanded mesenchymal stromal cell therapy: does it work in knee osteoarthritis? A pathway to clinical success. *Cell Mol Immunol*. 2023;20(6):626-650. doi:10.1038/s41423-023-01020-1

**Study type: Narrative Review; Level of evidence 4.**

14. Mautner K, Gottschalk M, Boden SD, et al. Cell-based versus corticosteroid injections for knee pain in osteoarthritis: a randomized phase 3 trial. *Nat Med*. 2023;29(12):3120-3126. doi:10.1038/s41591-023-02632-w

**Study type: RCT; Level of evidence 1.**

15. Joswig AJ, Mitchell A, Cummings KJ, et al. Repeated intra-articular injection of allogeneic mesenchymal stem cells causes an adverse response compared to autologous cells in the equine model. *Stem Cell Res Ther*. 2017;8(1):42. doi:10.1186/s13287-017-0503-8

**Study type: *in vivo* study.**

16. Pigott JH, Ishihara A, Wellman ML, Russell DS, Bertone AL. Investigation of the immune response to autologous, allogeneic, and xenogeneic mesenchymal stem cells after intra-articular injection in horses. *Veterinary Immunology and Immunopathology*. 2013;156(1-2):99-106. doi:10.1016/j.vetimm.2013.09.003

**Study type: *in vivo* study.**

17. Ardanaz N, Vázquez FJ, Romero A, et al. Inflammatory response to the administration of mesenchymal stem cells in an equine experimental model: effect of autologous, and single and repeat doses of pooled allogeneic cells in healthy joints. *BMC Vet Res*. 2016;12(1):65. doi:10.1186/s12917-016-0692-x

**Study type: *in vivo* study.**

18. Barrachina L, Remacha AR, Romero A, et al. Assessment of effectiveness and safety of repeat administration of proinflammatory primed allogeneic mesenchymal stem cells in an equine model of chemically induced osteoarthritis. *BMC Vet Res*. 2018;14(1):241. doi:10.1186/s12917-018-1556-3

**Study type: *in vivo* study.**

19. Barrachina L, Cequier A, Romero A, et al. Allo-antibody production after intraarticular administration of mesenchymal stem cells (MSCs) in an equine osteoarthritis model: effect of repeated administration, MSC inflammatory stimulation, and equine leukocyte antigen (ELA) compatibility. *Stem Cell Res Ther*. 2020;11(1):52. doi:10.1186/s13287-020-1571-8

**Study type: *in vivo* study.**

20. Magri C, Schramme M, Febre M, et al. Comparison of efficacy and safety of single versus repeated intra-articular injection of allogeneic neonatal mesenchymal stem cells for treatment of osteoarthritis of the metacarpophalangeal/metatarsophalangeal joint in horses: A clinical pilot study. Fiorina P, ed. *PLoS ONE*. 2019;14(8):e0221317. doi:10.1371/journal.pone.0221317

**Study type: *in vivo* study.**

21. Rowland AL, Miller D, Berglund A, et al. Cross-matching of allogeneic mesenchymal stromal cells eliminates recipient immune targeting. *Stem Cells Translational Medicine*. 2021;10(5):694-710. doi:10.1002/sctm.20-0435

**Study type: *in vivo* study.**

22. Ao Y, Duan J, Xiong N, et al. Repeated intra-articular injections of umbilical cord-derived mesenchymal stem cells for knee osteoarthritis: a phase I, single-arm study. *BMC Musculoskelet Disord*. 2023;24(1):488. doi:10.1186/s12891-023-06555-y

**Study type: Case series; Level of evidence 4.**

23. De Windt TS, Vonk LA, Slaper-Cortenbach ICM, et al. Allogeneic mesenchymal stem cells stimulate cartilage regeneration and are safe for single-stage cartilage repair in humans upon mixture with recycled autologous chondrons. *Stem Cells*. 2017;35(1):256-264. doi:10.1002/stem.2475

**Study type: Clinical trial; Level of evidence 3.**

**SECTION 2**

**QUESTION 13**

**Is there a difference between Bone Marrow Aspirate (BMA) or Bone Marrow Concentrate (BMAC) for the management of knee OA?**

**Statement**

Current evidence is lacking controlled clinical studies directly comparing bone marrow aspirate (BMA) and bone marrow concentrate (BMAC) for the management of knee OA.

Nevertheless, data indicates that BMA obtained with the most appropriate instruments and technique provides a similar number of bone marrow mesenchymal stem/stromal cells (BM-MSC) as in single-spin BMAC from a sample harvested without specific techniques aimed at minimizing peripheral blood contamination. When using the same equipment and technique for bone marrow harvesting, BMAC (obtained by centrifugation) will result in a product with a higher cell number, although with a lower volume. **The consensus, therefore, agrees it is essential to adopt the most suitable technique and instrument for bone marrow collection (see Question 18) in order not to compromise the resulting product or concentration procedure when relevant.**

A double-spin BMAC protocol is reported to increase the cell concentration while significantly reducing the volume. Double-spin BMAC protocol produces a higher BM-MSCs number, which seem to positively influence clinical benefit and, therefore, when considering BMAC use for knee OA, the consensus group recommends the use of products with a double-spin protocol.

Grade of recommendation: D

Rating Score: Mean 8.5±0.6; Median 9 (7-9) Appropriate with strong agreement

**Literature summary:**

Native bone marrow contains approximately 1% HSCs and 0.01% bone marrow-derived cells (BM-MSCs) with respect to total nucleated cells^1–3^.

A sub-optimal harvesting site, the collection of large volumes per aspiration (> 2 ml), and the use of large syringes (> 10 ml) may result in the collection of peripheral blood rather than bone marrow, thus reducing the overall BM-MSC content^4,5^.

Bone marrow can either be used as freshly isolated (bone marrow aspirate, BMA) or as further manipulated through filtration or centrifugation process (bone marrow aspirate concentrate, BMAC). Both products are obtained in one-step procedure.

Noteworthy, often studies do not use the correct terminology, i.e. using BMA for centrifuged products and BMAC for unprocessed samples.

The advantage of using specific devices to obtain BMA is that, reducing the amount of peripheral blood contamination, it allows to obtain a product rich in MSCs without the need of further processing (such as centrifugation steps), thus reducing the time interval between harvesting and injection, making the procedure faster and minimizing the risk of contamination. BMAC is obtained using systems mostly based on centrifugation step(s) aimed to reduce the initial large volume of bone marrow/peripheral blood and obtain a final output enriched in mononuclear cells among which BM-MSC.

A pilot study included 10 patients with knee osteoarthritis treated by intra-articular and subchondral injections of BMA obtained by a centrifuge-free process and prospectively evaluated at baseline and up to 24-month of follow-up by IKDC score, KOOS subscales, and VAS pain. The resulting product (BMA) contained as many BM-MSCs as expected in a pure bone marrow sample, demonstrating that specifically designed instruments allow the harvesting of pure bone marrow, minimizing red blood collection while reducing donor site morbidity and patient discomfort. BMA was able to control inflammation and counteract the catabolic cascade triggered by an inflammatory stimulus in vitro^6^. The clinical evaluation showed significant improvements in all scores adopted, with stable results up to two years. However, the limitation of the study was the small sample size and the lack of control group.

A review including 4 preclinical and 18 clinical studies (4626 patients) showed an overall improvement in pain and function. Indeed, both BMA and BMAC are reported to provide clinical benefits in the treatment of knee OA. However, the clinical studies present significant heterogeneity, few patients, short-term follow-up, and overall poor methodology^7^.

Although there are no controlled human studies directly comparing BMAC and BMA for knee OA, the existing data show that the concentration of BM-MSCs per ml is equal in BMA^8–10^. Double spin protocol is reported to increase the cell concentration while significantly reducing the volume.

At the same time the use of BMA allows to save procedural time, it does not require additional equipment (i.e centrifuge) and most of the time it is harvested through a single harvest site by using a devoted tool. The harvest through a single hole was reported to result in lower patients’ discomfort in comparison to multiple harvesting sites while providing the same cell ratios as well as the same colony-forming ability^11^. This would suggest that BMAC, typically produced starting from a larger volume of bone marrow harvested without specific intstrumentation, would be less tolerated by patients as it may often require longer harvesting involving more than one site.

An observational study (level V) showed that although a higher cell count in bone marrow samples (> 4X10^8^ mononucleated cells) was associated with a greater reduction in pain, there were no significant differences in functional improvement including the LEFS and IKDC (follow-up: 1, 3, 6 and 12 months, and annually thereafter)^12^.

In a comparative in vitro study, bone marrow samples were harvested from both the iliac crests of 5 patients using two different systems, namely one that did not require additional procedures (BMA) and the other that did require an additional centrifugation after harvesting (BMAC). The findings showed that BMA obtained by using a devoted instrument produced concentrations of CFU-fs, CD34+ cells and CD117+ BM-derived cells that were comparable or greater to BMACs derived from the same patient^8^.

It is important to note that not all the production systems result in the same final product quality^9^ and therefore the clinical outcome may differ according to the product’s choice.

**References**

1. Friedenstein AJ, Latzinik NW, Grosheva AG, Gorskaya UF. Marrow microenvironment transfer by heterotopic transplantation of freshly isolated and cultured cells in porous sponges. *Exp Hematol*. 1982;10(2):217-227.

**Study type: *in vitro* study.**

2. Wexler SA, Donaldson C, Denning-Kendall P, Rice C, Bradley B, Hows JM. Adult bone marrow is a rich source of human mesenchymal “stem” cells but umbilical cord and mobilized adult blood are not. *Br J Haematol*. 2003;121(2):368-374. doi:10.1046/j.1365-2141.2003.04284.x

**Study type: *in vitro* study.**

3. Krause DS, Fackler MJ, Civin CI, May WS. CD34: structure, biology, and clinical utility. *Blood*. 1996;87(1):1-13.

**Study type: Narrative review on in vitro findings.**

4. Narbona-Carceles J, Vaquero J, Suárez-Sancho SBS, Forriol F, Fernández-Santos ME. Bone marrow mesenchymal stem cell aspirates from alternative sources: is the knee as good as the iliac crest? *Injury*. 2014;45 Suppl 4:S42-47. doi:10.1016/S0020-1383(14)70009-9

**Study type: *in vitro* study.**

5. Hernigou P, Homma Y, Flouzat Lachaniette CH, et al. Benefits of small volume and small syringe for bone marrow aspirations of mesenchymal stem cells. *Int Orthop*. 2013;37(11):2279-2287. doi:10.1007/s00264-013-2017-z

**Study type: *in vitro* study.**

6. Viganò M, Ragni E, Di Matteo B, et al. A single step, centrifuge-free method to harvest bone marrow highly concentrated in mesenchymal stem cells: results of a pilot trial. *Int Orthop*. 2022;46(2):391-400. doi:10.1007/s00264-021-05243-7

**Study type: Pilot trial. Level of evidence 4.**

7. Cavallo C, Boffa A, Andriolo L, et al. Bone marrow concentrate injections for the treatment of osteoarthritis: evidence from preclinical findings to the clinical application. *Int Orthop*. 2021;45(2):525-538. doi:10.1007/s00264-020-04703-w

**Study type: Case series. Level of evidence 4.**

8. Scarpone M, Kuebler D, Chambers A, et al. Isolation of clinically relevant concentrations of bone marrow mesenchymal stem cells without centrifugation. *J Transl Med*. 2019;17(1):10. doi:10.1186/s12967-018-1750-x

**Study type: in vitro study**

9. Hegde V, Shonuga O, Ellis S, et al. A prospective comparison of 3 approved systems for autologous bone marrow concentration demonstrated nonequivalency in progenitor cell number and concentration. *J Orthop Trauma*. 2014;28(10):591-598. doi:10.1097/BOT.0000000000000113

**Study type: in vitro study**

10. McLain RF, Fleming JE, Boehm CA, Muschler GF. Aspiration of osteoprogenitor cells for augmenting spinal fusion: comparison of progenitor cell concentrations from the vertebral body and iliac crest. *J Bone Joint Surg Am*. 2005;87(12):2655-2661. doi:10.2106/JBJS.E.00230

**Study type: in vitro study**

11. Oliver K, Awan T, Bayes M. Single- Versus Multiple-Site Harvesting Techniques for Bone Marrow Concentrate: Evaluation of Aspirate Quality and Pain. *Orthop J Sports Med*. 2017;5(8):2325967117724398. doi:10.1177/2325967117724398

**Study type: in vitro study and cohort study; Level of evidence 4.**

12. Centeno CJ, Al-Sayegh H, Bashir J, Goodyear S, Freeman MD. A dose response analysis of a specific bone marrow concentrate treatment protocol for knee osteoarthritis. *BMC Musculoskelet Disord*. 2015;16:258. doi:10.1186/s12891-015-0714-z

**Study type: Observational Study. Level of evidence 4.**

**QUESTION 14**

**Is there a difference between mechanical stromal vascular fraction (SVF) and microfragmented adipose tissue (MFAT) for the management of knee OA?**

**Statement**

Although different in composition and structure, mechanical stromal vascular fraction (SVF) and microfragmented adipose tissue (MFAT) show a similar safety and efficacy profile for the treatment of knee OA, with satisfactory subjective results up to 24 months. Until further studies are conducted to determine whether one product is clinically superior to the other, **the consensus group currently does not support one type of adipose-derived CBT over the other and considers both mechanical SVF and MFAT valid options for the management of knee OA when this approach is considered.**

Grade of recommendation: D

Rating Score: Mean 8.4±0.6; Median 8 (7-9) Appropriate with strong agreement

**Literature summary:**

Adipose tissue is a highly vascularized structure, mainly composed of connective tissue and several cell types, including stromal players, like AT-MSCs defining the adipose SVF^1^ that is associated to the therapeutic potential of adipose-derived products. Interestingly, the adipose tissue SVF contains up to 3% of MSCs, whereas in bone-marrow it is reported around 0.002%^2^.

Two different procedures are currently used to prepare minimally manipulated AT-MSC-containing products at the point of care, namely SVF and microfragmented adipose tissue MFAT.

SVF refers to an adipocyte-free cell suspension, while MFAT is composed of clusters of blood- and lipids-free adipose tissue ranging from tens to few hundred micrometers in diameter, where AT-MSCs are shuttled within their niche^3,4^.

It has to be noted that only mechanical SVF prepared at the point of care can be considered as a minimally manipulated product in most countries, while SVF achieved with proteolytic enzymes (i.e collagenase) would not satisfy this definition although could be available as POC products. Although studies consistently show that enzymatic breakdown of the extracellular matrix affords significantly greater efficiency to the cell collection process resulting in a higher frequency of cells, SVF isolated through mechanical methods is associated with less cost and time for preparation^4^. As a consequence, almost all the studies available in literature on SVF are about the one prepared mechanically.

Both SVF and MFAT are currently used for the conservative management of knee OA as shown in a number of meta-analyses reporting them as valid and safe injective treatments concerning pain reduction, functional improvement, and quality of life up to two years after injection. Nevertheless, the number and the overall quality of the RCTs included is limited, as reported by several systematic reviews and meta-analysis^5–9^.

No controlled human studies exist directly comparing SVF and microfragmented adipose tissue (mFAT) for knee OA. One study is ongoing but recruitment has not started as per last update posted^10^.

Regarding SVF, a level I RCT compared SVF to placebo for knee OA, concluding that SVF improved WOMAC score at 12 months by 89.5% compared to saline^11^. Similarly, two other Level I and II RCTs comparing SVF to HA reported marked improvement of the clinical symptoms without adverse events at 12 months^12,13^. Interestingly, a longer term RCT on 126 patients, showed that the VAS and WOMAC scores in the SVF group were significantly better than those in the HA group during the 5-year follow-up. Up to 5 years after SVF treatment, acceptable clinical state was present for approximately 60% of patients^14^. Another clinical prospective controlled study vs placebo reported that the SVF therapy is effective in the recovery of OA patients of KL3 grade at 24 month follow-up^15^. Also, in a prospective case series of 57 patients, the short-term clinical effects (12 months) of intra-articular SVF cell injection on knee OA were defined as excellent^16^.

Regarding MFAT, a level II RCT compared 3 injections of PRP+HA to mFAT for knee OA (50 patients), reported similar improvements in the two groups at 12 months, with better results in Tegner activity scale for mFAT at 6 and 12 months and in KOOS symptoms subscale at 6 months^17^.

A number of case series investigated the treatment of knee OA with mFAT (>100 treatments each). Data show no serious adverse event after the treatment, while consistently reporting improvements in pain and function score at 12- and 24-month follow ups^18–22^.

A level II prospective non-randomized clinical trial showed that a single intra-articular injection of autologous mFAT in patients with knee OA grade III and IV, induced a substantial decrease of VAS at 24 months and a significant increase of glycosaminoglycans deposition in specific areas of the treated knee joint^23^. Similar results were showed in an interventional prospective study, in which after a single intra-articular injection of mFAT, clinical improvement with no adverse events at the 18-month follow-up were obtained, even if the best improvement in knee function were at 9 months post-injection^24^.

On the contrary, in a prospective case series an early clinical improvement after a single intra-articular injection with MFAT was observed but with a poor response rate of 45% at 12 months^25^.

In conclusion, both SVF and mFAT appear to have similar safety and efficacy profiles for knee OA. Further RCTs are needed to confirm the efficacy of these techniques over other regenerative medicine therapies, as well as to determine the most effective type of adipose-derived product for this application.

Interestingly, in a preclinical study on a rabbit knee OA model, the Authors showed distinct migration pathways for SVF and mFAT cells. Initially, SVF cells reach synovial membrane and at later time point they move towards cartilage and meniscus; mFAT cells home to cartilage tissue in the first days after injection, and then reach synovial membrane at later time points (30 days). The Authors also observed that SVF improved histological appearance of synovial membrane while mFAT had a greater effect on cartilage^26^. These observations may guide future research in the view of synovial membrane and cartilage specific therapies.

**References**

1. Casteilla L, Planat-Benard V, Laharrague P, Cousin B. Adipose-derived stromal cells: Their identity and uses in clinical trials, an update. *World J Stem Cells*. 2011;3(4):25-33. doi:10.4252/wjsc.v3.i4.25

**Study type: Narrative Review; Level of evidence 4.**

2. Fraser JK, Zhu M, Wulur I, Alfonso Z. Adipose-derived stem cells. *Methods Mol Biol*. 2008;449:59-67. doi:10.1007/978-1-60327-169-1_4

**Study type: Narrative Review; Level of evidence 4.**

3. Shah FS, Wu X, Dietrich M, Rood J, Gimble JM. A non-enzymatic method for isolating human adipose tissue-derived stromal stem cells. *Cytotherapy*. 2013;15(8):979-985. doi:10.1016/j.jcyt.2013.04.001

**Study type: *in vitro* study.**

4. Aronowitz JA, Lockhart RA, Hakakian CS. Mechanical versus enzymatic isolation of stromal vascular fraction cells from adipose tissue. *Springerplus*. 2015;4:713. doi:10.1186/s40064-015-1509-2

**Study type: *in vitro* study.**

5. Kim KI, Kim MS, Kim JH. Intra-articular Injection of Autologous Adipose-Derived Stem Cells or Stromal Vascular Fractions: Are They Effective for Patients With Knee Osteoarthritis? A Systematic Review With Meta-analysis of Randomized Controlled Trials. *Am J Sports Med*. 2023;51(3):837-848. doi:10.1177/03635465211053893

**Study type: Meta-analysis; Level of evidence 1.**

6. Yang Y, Lan Z, Yan J, et al. Effect of intra-knee injection of autologous adipose stem cells or mesenchymal vascular components on short-term outcomes in patients with knee osteoarthritis: an updated meta-analysis of randomized controlled trials. *Arthritis Res Ther*. 2023;25(1):147. doi:10.1186/s13075-023-03134-3

**Study type: Meta-analysis; Level of evidence 2.**

7. Shanmugasundaram S, Vaish A, Chavada V, Murrell WD, Vaishya R. Assessment of safety and efficacy of intra-articular injection of stromal vascular fraction for the treatment of knee osteoarthritis-a systematic review. *Int Orthop*. 2021;45(3):615-625. doi:10.1007/s00264-020-04926-x

**Study type: Systematic Review; Level of evidence 4.**

8. Agarwal N, Mak C, Bojanic C, To K, Khan W. Meta-Analysis of Adipose Tissue Derived Cell-Based Therapy for the Treatment of Knee Osteoarthritis. *Cells*. 2021;10(6):1365. doi:10.3390/cells10061365

**Study type: Meta-analysis; Level of evidence 2.**

9. Boada-Pladellorens A, Avellanet M, Pages-Bolibar E, Veiga A. Stromal vascular fraction therapy for knee osteoarthritis: a systematic review. *Ther Adv Musculoskelet Dis*. 2022;14:1759720X221117879. doi:10.1177/1759720X221117879

**Study type: Systematic Review; Level of evidence 4.**

10. Krześniak AM, Radzimowski K, Stolarczyk A. Comparison of the treatment results of knee osteoarthritis using adipose tissue mesenchymal stromal cells derived through enzymatic digestion and mechanically fragmented adipose tissue. *Medicine (Baltimore)*. 2021;100(9):e24777. doi:10.1097/MD.0000000000024777

**Study type: Study protocol; Level of evidence 4.**

11. Garza JR, Campbell RE, Tjoumakaris FP, et al. Clinical Efficacy of Intra-articular Mesenchymal Stromal Cells for the Treatment of Knee Osteoarthritis: A Double-Blinded Prospective Randomized Controlled Clinical Trial. *Am J Sports Med*. 2020;48(3):588-598. doi:10.1177/0363546519899923

**Study type: RCT; Level of evidence 1.**

12. Hong Z, Chen J, Zhang S, et al. Intra-articular injection of autologous adipose-derived stromal vascular fractions for knee osteoarthritis: a double-blind randomized self-controlled trial. *Int Orthop*. 2019;43(5):1123-1134. doi:10.1007/s00264-018-4099-0

**Study type: RCT; Level of evidence 1.**

13. Zhang Y, Bi Q, Luo J, Tong Y, Yu T, Zhang Q. The Effect of Autologous Adipose-Derived Stromal Vascular Fractions on Cartilage Regeneration Was Quantitatively Evaluated Based on the 3D-FS-SPGR Sequence: A Clinical Trial Study. *Biomed Res Int*. 2022;2022:2777568. doi:10.1155/2022/2777568

**Study type: RCT; Level of evidence 1.**

14. Zhang S, Xu H, He B, et al. Mid-term prognosis of the stromal vascular fraction for knee osteoarthritis: a minimum 5-year follow-up study. *Stem Cell Res Ther*. 2022;13(1):105. doi:10.1186/s13287-022-02788-1

**Study type: RCT; Level of evidence 1.**

15. Tran TDX, Wu CM, Dubey NK, et al. Time- and Kellgren−Lawrence grade-dependent changes in intra-articularly transplanted stromal vascular fraction in osteoarthritic patients. *Cells*. 2019;8(4):308. doi:10.3390/cells8040308

**Study type: Uncontrolled Clinical Trial; Level of evidence 3.**

16. Tsubosaka M, Matsumoto T, Sobajima S, Matsushita T, Iwaguro H, Kuroda R. The influence of adipose-derived stromal vascular fraction cells on the treatment of knee osteoarthritis. *BMC Musculoskelet Disord*. 2020;21(1):207. doi:10.1186/s12891-020-03231-3

**Study type: Prospective Uncontrolled Clinical Trial; Level of evidence 3.**

17. Dallo I, Szwedowski D, Mobasheri A, Irlandini E, Gobbi A. A Prospective Study Comparing Leukocyte-Poor Platelet-Rich Plasma Combined with Hyaluronic Acid and Autologous Microfragmented Adipose Tissue in Patients with Early Knee Osteoarthritis. *Stem Cells Dev*. 2021;30(13):651-659. doi:10.1089/scd.2021.0053

**Study type: RCT; Level of evidence 2.**

18. Heidari N, Noorani A, Slevin M, et al. Patient-Centered Outcomes of Microfragmented Adipose Tissue Treatments of Knee Osteoarthritis: An Observational, Intention-to-Treat Study at Twelve Months. *Stem Cells Int*. 2020;2020:8881405. doi:10.1155/2020/8881405

**Study type: Prospective Observational Study; Level of evidence 3.**

19. Heidari N, Borg TM, Olgiati S, et al. Microfragmented Adipose Tissue Injection (MFAT) May Be a Solution to the Rationing of Total Knee Replacement: A Prospective, Gender-Bias Mitigated, Reproducible Analysis at Two Years. *Stem Cells Int*. 2021;2021:9921015. doi:10.1155/2021/9921015

**Study type: Non-randomized Clinical Trial; Level of evidence 3.**

20. Gobbi A, Dallo I, Rogers C, et al. Two-year clinical outcomes of autologous microfragmented adipose tissue in elderly patients with knee osteoarthritis: a multi-centric, international study. *Int Orthop*. 2021;45(5):1179-1188. doi:10.1007/s00264-021-04947-0

**Study type: Non-randomized Clinical Trial; Level of evidence 3.**

21. Hogaboom N, D’Amico E, Mautner K, Rogers C, Malanga G. A 12-Month Cohort Study to Investigate Changes in Patient-Reported Outcomes after Intra-articular Injection of Micro-Fragmented Adipose Tissue for Knee Osteoarthritis. *Bio Orthop J*. 2021;3(1):e19-e28. doi:10.22374/boj.v3i1.13

**Study type: Prospective Uncontrolled Clinical Trial; Level of evidence 3.**

22. Borg TM, Heidari N, Noorani A, et al. Gender-Specific Response in Pain and Function to Biologic Treatment of Knee Osteoarthritis: A Gender-Bias-Mitigated, Observational, Intention-to-Treat Study at Two Years. *Stem Cells Int*. 2021;2021:6648437. doi:10.1155/2021/6648437

**Study type: Observational Study; Level of evidence 3.**

23. Borić I, Hudetz D, Rod E, et al. A 24-Month Follow-Up Study of the Effect of Intra-Articular Injection of Autologous Microfragmented Fat Tissue on Proteoglycan Synthesis in Patients with Knee Osteoarthritis. *Genes (Basel)*. 2019;10(12):1051. doi:10.3390/genes10121051

**Study type: Prospective Observational Study; Level of evidence 3.**

24. Yu Y, Lu Q, Li S, et al. Intra-Articular Injection of Autologous Micro-Fragmented Adipose Tissue for the Treatment of Knee Osteoarthritis: A Prospective Interventional Study. *J Pers Med*. 2023;13(3):504. doi:10.3390/jpm13030504

**Study type: Prospective Interventional Study; Level of evidence 4.**

25. Van Genechten W, Vuylsteke K, Martinez PR, Swinnen L, Sas K, Verdonk P. Autologous Micro-Fragmented Adipose Tissue (MFAT) to Treat Symptomatic Knee Osteoarthritis: Early Outcomes of a Consecutive Case Series. *J Clin Med*. 2021;10(11):2231. doi:10.3390/jcm10112231

**Study type: Case series; Level of evidence 4.**

26. Desando G, Bartolotti I, Martini L, et al. Regenerative Features of Adipose Tissue for Osteoarthritis Treatment in a Rabbit Model: Enzymatic Digestion Versus Mechanical Disruption. *Int J Mol Sci*. 2019;20(11):2636. doi:10.3390/ijms20112636

**Study type: *In vivo* study**

**QUESTION 15**

**Is there a clinical difference between expanded-CBT and point-of-care (POC)-CBT for the management of knee OA?**

The literature involving direct comparisons between expanded-CBT and point-of-care (POC)-CBT is sparse and limited. Treatments involving both expanded cells and POC products have been shown to be safe treatment options and to have the ability to provide clinical benefit for up to 12-24 months.

Expanded cell products have been shown to provide more consistent cell numbers, although they entail a higher production cost and a more complex two-stage procedure (in autologous products). Discrepancies in the clinical settings, in production protocols, and the lack of stratification of OA patients based on the radiologic classification currently limit any recommendation on the use of either product group in clinical practice. Therefore, **the consensus group does not recommend the use of one group over the other and currently considers both expanded-CBT and POC-CBT as acceptable products for the management of knee OA.**

Grade of recommendation: C

Rating Score: Mean 8.0±1.3; Median 8 (5-9) Appropriate with relative agreement

**Literature summary:**

Intra-articular Injectable cell-based products represent a promising tool for the conservative treatment of the diffuse chondral damages of the knee OA^1^. In the clinical practice MSCs, particularly from bone marrow and adipose tissue, are the most frequently used cells, both as expanded and non-expanded forms, since they are safe and harvestable with minimally invasive procedures.

Cell expansion allows for the acquisition of a precisely determined number of homogeneous cells, ensuring a high reproducibility of the procedure. Limitations are related to extensive in-vitro cell manipulation, entailing a high cost of the two-step procedure and stringent regulations. POC products (minimally manipulated products) such as BMAC, SVF or MFAT represent a group of minimally manipulated products which involve production via one-step procedures, with lower costs and preservation of MSCs physiological niche. Nevertheless, the amount of MSCs present in these products is less consistent and more variable compared to expanded MSCs products^2^.

The literature search found no RCTs in which a comparison between expanded cells and cell concentrates for the treatment of knee osteoarthritis (OA) was performed. However, two cohort studies compared the efficacy of the intra-articular injection of AT-MSCs and SVF^3,4^. In the first study^3^, 80 knee OA patients were divided into two groups: 42 patients (59 knees) received an intra-articular injection of AT-MSCs and 38 patients (69 knees) received SVF. Positive response was determined at 6-months using the OMERACT-OARSI criteria for both treatments. In the AT-MSC group symptoms improved earlier (by 3 months; P < .05) and pain VAS decreased to a greater degree (55%; P < .05) compared with the SVF group (44%), with no major complications in either group. The SVF group had a higher frequency of knee effusion (SVF 8%, AT-MSCs 2%) but minor complications related to the fat harvest site (SVF 34%, AT-MSCs 5%).

In the second study^4^, the same 80 patients were assessed through a longer follow-up (24 months). AT-MSCs and SVF injections both substantially improved knee pain and function at all follow-up time points, although AT-MSC injections demonstrated significantly better improvements with regard to the MCID and PASS for the pain VAS and the MCID for the KOOS at 12 months.

Knees treated with AT-MSCs for KL grade 2/3 OA had significantly superior outcomes compared with those with KL grade 4 OA for the KOOS (P = .01) and pain VAS (P = .03), but no such difference was observed in knees treated with SVF. Three patients receiving AT-MSCs (7%; all KL grade 3) sought additional nonoperative treatment by 24 months versus 9 patients receiving SVF (24%; all KL grade 3) (P = .06). Interestingly, there appears to be no benefit to a booster AT-MSC injection after initial treatment. Notably, patients in the AT-MSC cohort reported more injection-site pain and swelling after the booster injection than after the initial injection (P < .01).

The use of AT-MSCs over SVF was advantageous in this study, given less donor-site morbidity and superior outcomes at 2 years.

Given the lack of other direct comparisons an evaluation of the clinical outcomes of MSCs in the treatment of knee OA was conducted and reported separately for expanded and concentrated cells.

Expanded cells

A systematic literature review and meta-analysis of 6 RTC, with 203 participants was found. Four of these 6 studies were performed with BM-MSCs, while the remaining studies were performed with AT-MSCs and placenta-derived MSCs^5^. This meta-analysis has demonstrated that intra-articular injection of MSCs can significantly enhance pain and functional scores over a short-term period (6-12 months) and cartilage repair assessed at 6-12 months by magnetic resonance imaging^5^.

Another Level 2 systematic review and dual network meta-analysis ^6^ included 16 RCTs with 612 patients enrolled^6^. Their analysis indicated that the intra-articular delivery of BM-MSCs, AT-MSCs and UC-MSCs is more effective in alleviating pain and improving function than cell-free therapy (PRP, HA, and saline) for managing OA assessing a follow-up period of 6-12 months^6^. Additionally, this work showed that AT-MSCs and UC-MSCs were preferable to BM-MSCs for treating OA^6^.

Ossendorff et al. created a systematic review with 82 studies, including RTC, prospective cohort studies (level 1 and 2 studies), retrospective comparative trials (level 3 studies) and therapeutic case series (level 4 studies), focusing on AT-MSCs and SVF injection, of which 13 were related to the knee injection of expanded AT-MSCs. Despite the high heterogeneity in terms of utilized protocols, AT-MSCs demonstrated an enhancement of tissue regeneration and healing in preclinical studies^7^.

POC products

A level 4 systemtic review on cell concentrates collected 23 articles about the use of SVF or BMAC for the treatment of knee OA^8^. In regard to the clinical outcome, the majority of studies included subjective clinical scores (such as WOMAC, IKDC, KOOS, or KSS), where only a few performed MRI prior to and following the procedure^9,10^. Overall, these studies suggested significant improvement of relief pain, improved function, and repair cartilage defects in patients with knee OA following treatments with BMAC and SVF injections.

The above-mentioned systematic review Ossendorff et al.^7^ additionally considered 26 additional studies dealing with SVF treatment of knee OA, highlighting the safety and the short-term (1-3-6 months) beneficial effects of these products, but not recommending the use of these products in clinical practice^7^.

Another systematic literature review and meta-analysis^11^ comprising 10 clinical studies, reporting the outcomes of patients who received a single BMAC or SVF injection in the knee joint to manage osteoarthritic changes of any degree. A single BMAC or SVF injection into the OA knee joints resulted in symptomatic improvement at short-term and longer term follow-up (6-24 months). However, SVF seemed to be more effective than BMAC in the reduction of knee pain. There was significant variation in the BMAC and SVF injection preparation techniques used across the studies and a lack of stratification of outcomes based on the radiologic classification of OA. Therefore, the authors suggest to consider these results with caution^11^.

The lack of studies using both expanded cells and POC products impedes the direct comparison of the clinical efficacy between these two approaches. Nevertheless, both are reported to provide clinical benefit at medium term follow up, without marked differences in the subjective outcomes.

**References**

1. Fusco G, Gambaro FM, Di Matteo B, Kon E. Injections in the osteoarthritic knee: a review of current treatment options. *EFORT Open Rev*. 2021;6(6):501-509. doi:10.1302/2058-5241.6.210026

**Study type: Systematic Review; Level of evidence 3.**

2. Lopa S, Colombini A, Moretti M, de Girolamo L. Injective mesenchymal stem cell-based treatments for knee osteoarthritis: from mechanisms of action to current clinical evidences. *Knee Surg Sports Traumatol Arthrosc*. 2019;27(6):2003-2020. doi:10.1007/s00167-018-5118-9

**Study type: Systematic Review; Level of evidence 3.**

3. Yokota N, Hattori M, Ohtsuru T, et al. Comparative Clinical Outcomes After Intra-articular Injection With Adipose-Derived Cultured Stem Cells or Noncultured Stromal Vascular Fraction for the Treatment of Knee Osteoarthritis. *Am J Sports Med*. 2019;47(11):2577-2583. doi:10.1177/0363546519864359

**Study type: Cohort Study; Level of evidence 3.**

4. Yokota N, Lyman S, Hanai H, Shimomura K, Ando W, Nakamura N. Clinical Safety and Effectiveness of Adipose-Derived Stromal Cell vs Stromal Vascular Fraction Injection for Treatment of Knee Osteoarthritis: 2-Year Results of Parallel Single-Arm Trials. *Am J Sports Med*. 2022;50(10):2659-2668. doi:10.1177/03635465221107364

**Study type: Cohort Study; Level of evidence 2.**

5. Kim SH, Djaja YP, Park YB, Park JG, Ko YB, Ha CW. Intra-articular Injection of Culture-Expanded Mesenchymal Stem Cells Without Adjuvant Surgery in Knee Osteoarthritis: A Systematic Review and Meta-analysis. *Am J Sports Med*. 2020;48(11):2839-2849. doi:10.1177/0363546519892278

**Study type: Systematic Review and meta-analysis; Level of evidence 2.**

6. Zhang Y, Yang H, He F, Zhu X. Intra-articular injection choice for osteoarthritis: making sense of cell source-an updated systematic review and dual network meta-analysis. *Arthritis Res Ther*. 2022;24(1):260. doi:10.1186/s13075-022-02953-0

**Study type: Systematic Review and Network Meta-Analysis; Level of evidence 2.**

7. Ossendorff R, Menon A, Schildberg FA, et al. A Worldwide Analysis of Adipose-Derived Stem Cells and Stromal Vascular Fraction in Orthopedics: Current Evidence and Applications. *J Clin Med*. 2023;12(14):4719. doi:10.3390/jcm12144719

**Study type: Systematic Review; Level of evidence 4.**

8. Di Matteo B, Vandenbulcke F, Vitale ND, et al. Minimally Manipulated Mesenchymal Stem Cells for the Treatment of Knee Osteoarthritis: A Systematic Review of Clinical Evidence. *Stem Cells Int*. 2019;2019:1735242. doi:10.1155/2019/1735242

**Study type: Systematic Review; Level of evidence 3.**

9. Hernigou P, Auregan JC, Dubory A, Flouzat-Lachaniette CH, Chevallier N, Rouard H. Subchondral stem cell therapy versus contralateral total knee arthroplasty for osteoarthritis following secondary osteonecrosis of the knee. *Int Orthop*. 2018;42(11):2563-2571. doi:10.1007/s00264-018-3916-9

**Study type: RCT; Level of evidence 2.**

10. Vad V, Barve R, Linnell E, Harrison J. Knee Osteoarthritis Treated with Percutaneous Chondral-Bone Interface Optimization: A Pilot Trial. *Surgical Science*. 2016;7(1):1-12. doi:10.4236/ss.2016.71001

**Study type: Prospective Pilot study; Level of evidence 4.**

11. Bolia IK, Bougioukli S, Hill WJ, et al. Clinical Efficacy of Bone Marrow Aspirate Concentrate Versus Stromal Vascular Fraction Injection in Patients With Knee Osteoarthritis: A Systematic Review and Meta-analysis. *Am J Sports Med*. 2022;50(5):1451-1461. doi:10.1177/03635465211014500

**Study type: Systematic Review and meta-analysis; Level of evidence 4.**

**QUESTION 16**

**What should be the quality control measures for injectable CBT?**

**Statement**

Currently available literature on quality control measures for CBT is variable, spanning from a lack of information - mainly for point-of-care products - to exhaustive biological characterization - mainly for expanded cells.

For improving clinical practice and the quality of future studies as well as enabling improved comparison measures between studies and products, **the consensus group considers the reporting of cell characterization an important minimum quality control requirement**. The consensus group suggests adopting the “Minimum Information for Studies Evaluating Biologics in Orthopaedics (MIBO) requirements” when reporting data about CBT.

Grade of recommendation: D

Rating Score: Mean 8.7±0.5; Median 9 (8-9) Appropriate with strong agreement

*This statement is valid for both POC- and Expanded-CBT*

**Literature summary**

There are few recommendations concerning the biological characterization of cell based therapy especially when prepared in a point of care setting. We analyzed available studies and clinical trials and reported quality controls performed based on the origin of the tissue. As a result, practices remain heterogeneous in terms of biological product characterization.

*Microfragnented adipose tissue or Stromal Vascular Fraction*

The literature search highlighted the lack of biological characterization of injected products derived from adipose tissue in 13 from 20 studies identified. A few studies carried out a cell count^1^ associated with viability determination^2–6^. Only 2 studies performed a more complete characterization, including a colony forming unit analysis test and a sterility assay^7,8^. Among the studies which performed quality control, the injected product was mainly stromal vascular fraction, except for 2 studies: one used MFAT^3^ and the other used microfat^8^. For both studies, part of the QC were performed after obtaining stromal vascular fraction following enzymatic digestion and the study by Louis et al^8^ also performed GF quantification after microfat incubation in a dedicated media and sterility testing directly on microfat.

*Bone marrow concentrate*

As with adipose tissue, almost the majority of the studies^6–13^ does not provide quality control (QC) when bone marrow concentrate is injected.

When performed, QC includes a simple cell count^9,10^ or a more complete characterization including viability and cell surface marker using flow cytometry^11–13^. One study performed clonogenic CFU assay and measure of IL-1Ra protein concentrations^13^.

*Expanded cells from adipose tissue*

Clinical studies using AT-MSC often report biological characterization. Cell identity was assessed by flow cytometry, cell counting and trilineage differentiation in vitro^14^. Viability and sterility tests (bacteria and/or mycoplasma and/or endotoxin) tests are clearly mentioned for a large part of the studies^15–24^. Two studies also performed genetic checks using Short Tandem Repeat detection technology^24^ or karyotypes^21^.

*Expanded cells from bone marrow*

All the studies using BM-MSC provide information about biological characterization. Description of cell surface markers were described in majority of the studies^25–29^ with associated viability^27–30^ and bacteriological sterility^28,29,31^ whereas endotoxins and mycoplasma determination were performed in only two studies^31,32^ and CFU-F clonogenic assay in one study^26^.

This literature research on quality control highlights a wide variety of practices, from a total absence of information to exhaustive biological characterization. These differences can be explained by different national regulations and the belonging of expanded cells to the ATMPs family and related regulation 1394/2007. Indeed, a number of regulatory texts apply to ATMPs quality controls, in compliance with certain monographs of the European Pharmacopoeia (<https://www.ema.europa.eu/en/human-regulatory/research-development/advanced-therapies/guidelines-relevant-advanced-therapy-medicinal-products>).

Regarding the case of the use of orthobiologics, minimum quality control recommendations are provided by the Minimum Information for Studies Evaluating Biologics in Orthopaedics (MIBO)^33^ system which suggests, among others, to report at least cellular composition and/or heterogeneity, immunophenotype and details of in vitro differentiation tested on batch and passage and percentage viability. Here below the MIBO checklist.


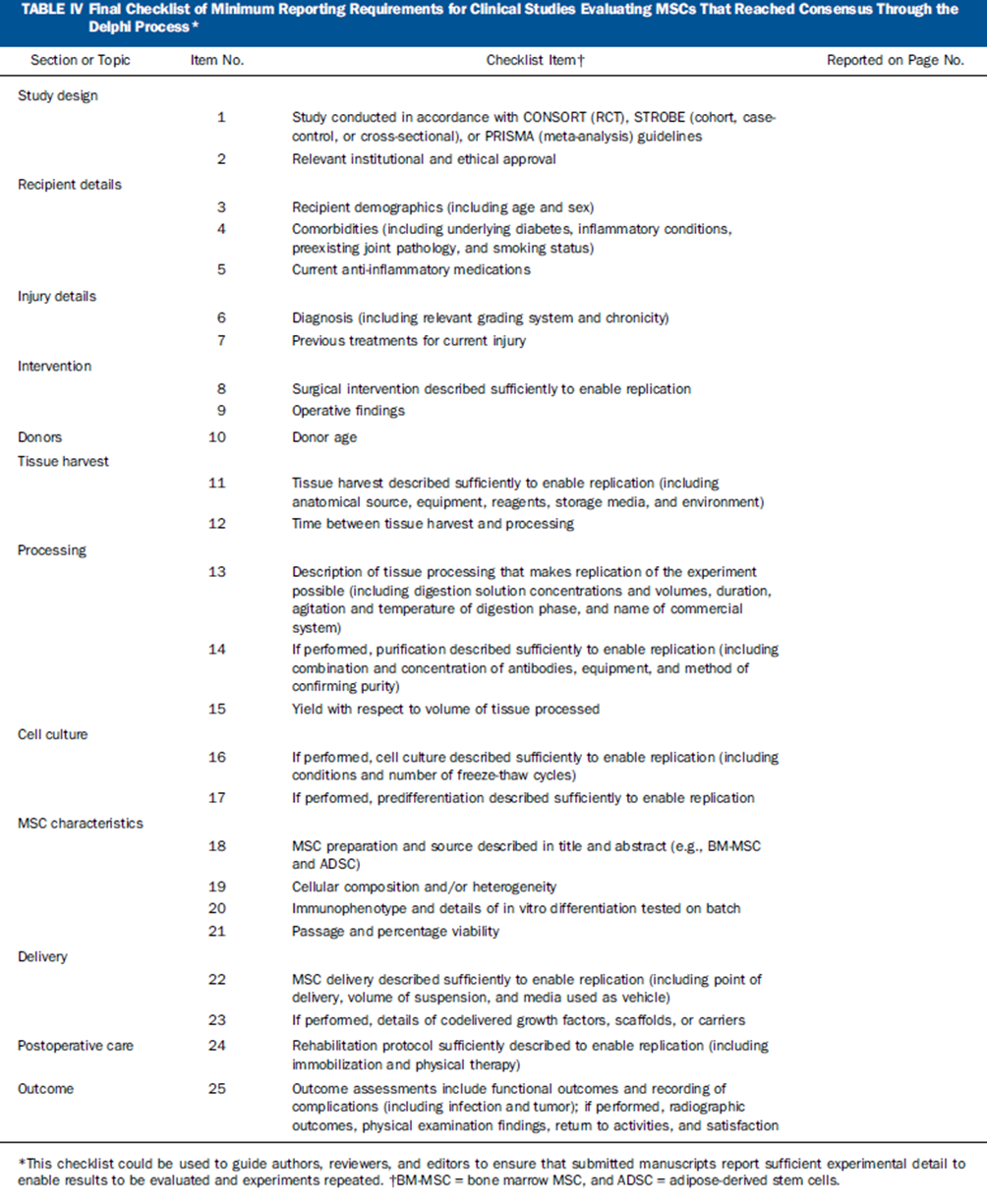


**References:**

1. Fodor PB, Paulseth SG. Adipose Derived Stromal Cell (ADSC) Injections for Pain Management of Osteoarthritis in the Human Knee Joint. *Aesthet Surg J*. 2016;36(2):229-236. doi:10.1093/asj/sjv135

**Study Type: Uncontrolled clinical trial; Level of evidence 4.**

2. Tsubosaka M, Matsumoto T, Sobajima S, Matsushita T, Iwaguro H, Kuroda R. The influence of adipose-derived stromal vascular fraction cells on the treatment of knee osteoarthritis. *BMC Musculoskelet Disord*. 2020;21(1):207. doi:10.1186/s12891-020-03231-3

**Study type: Prospective Uncontrolled Clinical Trial; Level of evidence 3.**

3. Hudetz D, Borić I, Rod E, et al. Early results of intra-articular micro-fragmented lipoaspirate treatment in patients with late stages knee osteoarthritis: a prospective study. *Croat Med J*. 2019;60(3):227-236. doi:10.3325/cmj.2019.60.227

**Study Type: Case series; Level of evidence 4.**

4. Zhang S, Xu H, He B, et al. Mid-term prognosis of the stromal vascular fraction for knee osteoarthritis: a minimum 5-year follow-up study. *Stem Cell Res Ther*. 2022;13(1):105. doi:10.1186/s13287-022-02788-1

**Study Type: RCT; Level of evidence 1.**

5. Zhang Y, Bi Q, Luo J, Tong Y, Yu T, Zhang Q. The Effect of Autologous Adipose-Derived Stromal Vascular Fractions on Cartilage Regeneration Was Quantitatively Evaluated Based on the 3D-FS-SPGR Sequence: A Clinical Trial Study. Du J, ed. *BioMed Research International*. 2022;2022:1-17. doi:10.1155/2022/2777568

**Study Type: RCT; Level of evidence 1.**

6. Song JS, Hong KT, Kim NM, et al. Implantation of allogenic umbilical cord blood-derived mesenchymal stem cells improves knee osteoarthritis outcomes: Two-year follow-up. *Regenerative Therapy*. 2020;14:32-39. doi:10.1016/j.reth.2019.10.003

**Study Type: Case series; Level of evidence 4.**

7. Garza JR, Campbell RE, Tjoumakaris FP, et al. Clinical Efficacy of Intra-articular Mesenchymal Stromal Cells for the Treatment of Knee Osteoarthritis: A Double-Blinded Prospective Randomized Controlled Clinical Trial. *Am J Sports Med*. 2020;48(3):588-598. doi:10.1177/0363546519899923

**Study Type: RCT; Level of evidence 1.**

8. Louis ML, Dumonceau RG, Jouve E, et al. Intra-Articular Injection of Autologous Microfat and Platelet-Rich Plasma in the Treatment of Knee Osteoarthritis: A Double-Blind Randomized Comparative Study. *Arthroscopy: The Journal of Arthroscopic & Related Surgery*. 2021;37(10):3125-3137.e3. doi:10.1016/j.arthro.2021.03.074

**Study Type: RCT; Level of evidence 2.**

9. Centeno C, Sheinkop M, Dodson E, et al. A specific protocol of autologous bone marrow concentrate and platelet products versus exercise therapy for symptomatic knee osteoarthritis: a randomized controlled trial with 2 year follow-up. *J Transl Med*. 2018;16(1):355. doi:10.1186/s12967-018-1736-8

**Study Type: RCT; Level of evidence 1.**

10. Centeno CJ, Al-Sayegh H, Bashir J, Goodyear S, Freeman MD. A dose response analysis of a specific bone marrow concentrate treatment protocol for knee osteoarthritis. *BMC Musculoskelet Disord*. 2015;16(1):258. doi:10.1186/s12891-015-0714-z

**Study Type: Observational Study; Level of evidence 4.**

11. Shapiro SA, Arthurs JR, Heckman MG, et al. Quantitative T2 MRI Mapping and 12-Month Follow-up in a Randomized, Blinded, Placebo Controlled Trial of Bone Marrow Aspiration and Concentration for Osteoarthritis of the Knees. *Cartilage*. 2019;10(4):432-443. doi:10.1177/1947603518796142

**Study Type: RCT; Level of evidence 1.**

12. Dulic O, Rasovic P, Lalic I, et al. Bone Marrow Aspirate Concentrate versus Platelet Rich Plasma or Hyaluronic Acid for the Treatment of Knee Osteoarthritis. *Medicina*. 2021;57(11):1193. doi:10.3390/medicina57111193

**Study Type: RCT; Level of evidence 2.**

13. Wells K, Klein M, Hurwitz N, et al. Cellular and Clinical Analyses of Autologous Bone Marrow Aspirate Injectate for Knee Osteoarthritis: A Pilot Study. *PM&R*. 2021;13(4):387-396. doi:10.1002/pmrj.12429

**Study Type: Observational Study; Level of evidence 4.**

14. Song Y, Du H, Dai C, et al. Human adipose-derived mesenchymal stem cells for osteoarthritis: a pilot study with long-term follow-up and repeated injections. *Regenerative Medicine*. 2018;13(3):295-307. doi:10.2217/rme-2017-0152

**Study Type: Clinical trial; Level of evidence 3.**

15. Khoury MA, Chamari K, Tabben M, et al. Knee osteoarthritis: clinical and MRI outcomes after multiple intra-articular injections with expanded autologous adipose-derived stromal cells or platelet-rich plasma. *Cartilage*. 2023;14(4):433-444. doi:10.1177/19476035231166127

Study Type: RCT; Level of evidence 2.

16. Chen HH, Chen YC, Yu SN, et al. Infrapatellar fat pad-derived mesenchymal stromal cell product for treatment of knee osteoarthritis: a first-in-human study with evaluation of the potency marker. *Cytotherapy*. 2022;24(1):72-85. doi:10.1016/j.jcyt.2021.08.006

**Study Type: Uncontrolled Clinical trial; Level of evidence 3.**

17. Lee WS, Kim HJ, Kim KI, Kim GB, Jin W. Intra-articular injection of autologous adipose tissue-derived mesenchymal stem cells for the treatment of knee osteoarthritis: a phase IIb, randomized, placebo-controlled clinical trial. Stem cells Translational Medicine. 2019;8(6):504-511. doi:10.1002/sctm.18-0122

**Study Type: RCT; Level of evidence 2.**

18. Lu L, Dai C, Zhang Z, et al. Treatment of knee osteoarthritis with intra-articular injection of autologous adipose-derived mesenchymal progenitor cells: a prospective, randomized, double-blind, active-controlled, phase IIb clinical trial. *Stem Cell Res Ther*. 2019;10(1):143. doi:10.1186/s13287-019-1248-3

Study Type: RCT; Level of evidence 1.

19. Freitag J, Bates D, Wickham J, et al. Adipose-derived mesenchymal stem cell therapy in the treatment of knee osteoarthritis: a randomized controlled trial. *Regenerative Medicine*. 2019;14(3):213-230. doi:10.2217/rme-2018-0161

Study Type: RCT; Level of evidence 2.

20. Kim KI, Lee MC, Lee JH, et al. Clinical Efficacy and Safety of the Intra-articular Injection of Autologous Adipose-Derived Mesenchymal Stem Cells for Knee Osteoarthritis: A Phase III, Randomized, Double-Blind, Placebo-Controlled Trial. *Am J Sports Med*. 2023;51(9):2243-2253. doi:10.1177/03635465231179223

Study Type: RCT; Level of evidence 1.

21. Pers YM, Rackwitz L, Ferreira R, et al. Adipose Mesenchymal Stromal Cell-Based Therapy for Severe Osteoarthritis of the Knee: A Phase I Dose-Escalation Trial. *Stem Cells Translational Medicine*. 2016;5(7):847-856. doi:10.5966/sctm.2015-0245

**Study type: Uncontrolled clinical trial; Level of evidence 3.**

22. Jo CH, Chai JW, Jeong EC, et al. Intra-articular Injection of Mesenchymal Stem Cells for the Treatment of Osteoarthritis of the Knee: A 2-Year Follow-up Study. *Am J Sports Med*. 2017;45(12):2774-2783. doi:10.1177/0363546517716641

Study type: Prospective Cohort Study; Level of evidence 3.

23. Spasovski D, Spasovski V, Baščarević Z, et al. Intra‐articular injection of autologous adipose‐derived mesenchymal stem cells in the treatment of knee osteoarthritis. *The Journal of Gene Medicine*. 2018;20(1):e3002. doi:10.1002/jgm.3002

Study type: Case Series; Level of evidence 4.

24. Zhao X, Ruan J, Tang H, et al. Multi-compositional MRI evaluation of repair cartilage in knee osteoarthritis with treatment of allogeneic human adipose-derived mesenchymal progenitor cells. *Stem Cell Res Ther*. 2019;10(1):308. doi:10.1186/s13287-019-1406-7

**Study Type: Uncontrolled Clinical trial; Level of evidence 3.**

25. Lamo-Espinosa JM, Blanco JF, Sánchez M, et al. Phase II multicenter randomized controlled clinical trial on the efficacy of intra-articular injection of autologous bone marrow mesenchymal stem cells with platelet rich plasma for the treatment of knee osteoarthritis. *J Transl Med*. 2020;18(1):356. doi:10.1186/s12967-020-02530-6

Study Type: RCT; Level of evidence 2.

26. Bastos R, Mathias M, Andrade R, et al. Intra-articular injection of culture-expanded mesenchymal stem cells with or without addition of platelet-rich plasma is effective in decreasing pain and symptoms in knee osteoarthritis: a controlled, double-blind clinical trial. *Knee Surg Sports Traumatol Arthrosc*. 2020;28(6):1989-1999. doi:10.1007/s00167-019-05732-8

Study Type: RCT; Level of evidence 2.

27. Goncars V, Jakobsons E, Blums K, et al. The comparison of knee osteoarthritis treatment with single-dose bone marrow-derived mononuclear cells vs. hyaluronic acid injections. *Medicina*. 2017;53(2):101-108. doi:10.1016/j.medici.2017.02.002

Study Type: RCT; Level of evidence 2.

28. Garay‐Mendoza D, Villarreal‐Martínez L, Garza‐Bedolla A, et al. The effect of intra‐articular injection of autologous bone marrow stem cells on pain and knee function in patients with osteoarthritis. *Int J of Rheum Dis*. 2018;21(1):140-147. doi:10.1111/1756-185X.13139

**Study Type: Phase I/II Clinical trial; Level of evidence 3.**

29. Chahal J, Gómez-Aristizábal A, Shestopaloff K, et al. Bone Marrow Mesenchymal Stromal Cell Treatment in Patients with Osteoarthritis Results in Overall Improvement in Pain and Symptoms and Reduces Synovial Inflammation. *Stem Cells Translational Medicine*. 2019;8(8):746-757. doi:10.1002/sctm.18-0183

**Study Type: Uncontrolled Clinical Trial; Level of evidence 3.**

30. Orozco L, Munar A, Soler R, et al. Treatment of Knee Osteoarthritis With Autologous Mesenchymal Stem Cells: A Pilot Study. *Transplantation*. 2013;95(12):1535-1541. doi:10.1097/TP.0b013e318291a2da

**Study Type: Pilot Clinical study; Level of evidence 3.**

31. Soler R, Orozco L, Munar A, et al. Final results of a phase I–II trial using ex vivo expanded autologous Mesenchymal Stromal Cells for the treatment of osteoarthritis of the knee confirming safety and suggesting cartilage regeneration. *The Knee*. 2016;23(4):647-654. doi:10.1016/j.knee.2015.08.013

**Study Type: RCT; Level of evidence 2.**

32. Al-Najar M, Khalil H, Al-Ajlouni J, et al. Intra-articular injection of expanded autologous bone marrow mesenchymal cells in moderate and severe knee osteoarthritis is safe: a phase I/II study. *J Orthop Surg Res*. 2017;12(1):190. doi:10.1186/s13018-017-0689-6

**Study type: Prospective uncontrolled clinical trial; Level of evidence 3.**

33. Murray IR, Geeslin AG, Goudie EB, Petrigliano FA, LaPrade RF. Minimum Information for Studies Evaluating Biologics in Orthopaedics (MIBO): Platelet-Rich Plasma and Mesenchymal Stem Cells. *The Journal of Bone and Joint Surgery*. 2017;99(10):809-819. doi:10.2106/JBJS.16.00793

**Study Type: Guidelines; Level of evidence: 4**

**QUESTION 17**

**Does harvest location, harvest equipment and number of trajectories matter for adipose tissue harvest and preparation?**

**Statement**

Although in vitro studies show differences in terms of cell yield and performances among different anatomic harvest sites for adipose tissue, due to the lack of stringency and high heterogeneity in the design of the available clinical studies, **currently, it is not possible to recommend one harvest site over another**. Due to the different body mass composition of athletes, adipose tissue harvesting can be more complicated in these patients. **The consensus group, therefore, suggests choosing preparation methods that require smaller volume of adipose tissue**, or, in case of scarce material, to combine adipose tissue-derived products with platelet rich plasma (PRP) or other orthobiologics.

There is a general trend that mild harvest methods such as surgical resection and manual lipoaspiration are milder in terms of cell integrity, but due to the lack of clinical data comparing possible different methods, **it is not possible to drive any clear conclusion with regards to the optimal harvest equipment.**

Finally, no studies investigated the effect of the number of harvesting trajectories on adipose tissue CBT related to knee OA treatment and, therefore, **the consensus group cannot recommend an optimal number of harvest trajectories.**

Grade of recommendation: D

Rating Score: Mean 8.4±0.7; Median 9 (7-9) Appropriate with strong agreement

*This statement is valid for both POC- and Expanded-CBT*

**Literature summary**

*Harvest location*

Regarding anatomical location (harvest site) of adipose tissue there is a general overrepresentation of basic studies showing that there are cellular differences depending on the harvest site. The differences are primarily related to cellular quantity and trilineage differentiation performance of the adipose tissue-derived stem cells.

A study by Iyyanki et al.^1^. (n=15, paired-sample design) investigated subcutaneous adipose tissue from the abdomen, flank, and axilla, and found that the abdomen provided significantly higher number of SVF cells (but not AT-MSCs) The cultured cells showed trilineage differentiation performance, but a statistical comparison between harvest sites were not performed with regards to differentiation.

A study by Jurgens et al^2^. investigated non-paired subcutaneous adipose tissue from the abdomen (n=12) and hip/thigh region (n=10) reporting a higher higher frequency of AT-MSCs in the former, although no difference in terms of number of nucleated cells, cellular proliferation, phenotype, or osteogenic and chondrogenic differentiation potential.

A study by Padoin et al.^3^. (n=25, paired-sample design) investigated adipose tissue from the upper abdomen, lower abdomen, trochanteric region, inner thigh, knee, and the flank. The results showed that the lower abdomen and inner thigh resulted in higher lipoaspirate cell concentration compared to the other sites

A study by Fraser et al.^4^ (n=10, paired-sample design) investigated subcutaneous adipose tissue from the hip and abdomen, and reported that adipose tissue from the hip yielded 2.3-fold more CFU-F/unit volume and a 7-fold higher frequency of CFU-alkaline phosphatase positive (osteogenic marker) than the abdomen.

A study by Choudhery et al.^5^ reported no difference in AT-MSCs in terms of immunophenotype, CFU-F, population doublings, doubling time, adipogenic, osteogenic and chondrogenic differentiation, when harvested from various locations, although given the heavily biased study design these results are not completely reliable.

Selection of adipose tissue harvest site may thus be important for selection of cells suitable for treatment of osteoarthritis, both with regards to numbers and performance. However, due to high heterogeneity in the design of the available studies^6-9^ as well as to the lack of a direct comparison of the clinical efficacy between adipose-derived products harvested form different locations, it is not possible to recommend one harvest site over another. Currently, the level of evidence is low.

*Harvest equipment*

Regarding harvest equipment of adipose tissue there is a general overrepresentation of studies showing cellular differences depending on the chosen harvest equipment. The differences are related to cellular quantity, viability, trilineage differentiation performance (and cytokine expression) of AT-MSCs.

A study by Iyyanki et al.^1^ compared four harvest techniques of adipose tissue by Coleman’s technique (manual harvest of fat aspirated with a 3-mm blunt cannula and a 10-mL syringe without centrifugation, Coleman’s technique with centrifugation, machine-assisted liposuction or direct surgical excision. The results showed that direct excision provided higher number of SVF and AT-MSCs than the Coleman’s technique without centrifugation. There was a higher SVF when using Coleman’s technique with centrifugation compared to without (no difference in AT-MSCs). The machine-assisted liposuction (and the blood-oil waste) provided fewer SVF and AT-MSCs compared to Coleman’s technique without centrifugation.

A study by Schreml et al.^10^ compared surgical resection to liposuction. The results showed no difference in total nucleated cell count between the two methods. More viable cells were identified after liposuction. No difference was shown for adipogenic differentiation of the AT-MSCs, whereas AT-MSCs obtained by surgical resection had significantly higher osteogenic and chondrogenic differentiation potential

A study by Fraser et al.^4^ found that syringe suction resulted in a higher frequency of AT-MSCs compared to pump-assisted liposuction.

A study by Oedayrajsingh-Varma et al.^11^ found no difference between surgical resection and tumescent liposuction. However, ultrasound-assisted liposuction resulted in fewer proliferating AT-MSCs.

On the other hand, Duscher et al.^12^ reported no difference between a third-generation ultrasound-assisted liposuction device (containing a specialized probe or cannula to transmit ultrasound vibrations to the adipose tissue to emulsify the fat, decrease blood loss and tissue trauma) versus AT-MSCs obtained via standard suction-assisted lipoaspiration with regards to AT-MSC yield and viability, and differentiation potential. However, the lack of a clear description of the SAL procedure questions the reliability of the results.

Selection of harvest equipment of adipose tissue may thus be important for selection of cells suitable for treatment of OA, both with regards to viable numbers and performance.

There is a general trend that mild harvest methods such as surgical resection and manual lipoaspirations are milder on the cells, but due to lack of stringency and high heterogeneity in the design of the available literature the conclusion is in high risk of bias and the true effect might be markedly different from the estimated effect. Currently, the level of evidence is low.

*Number of trajectories*

No studies were identified investigating the effect of the number of trajectories on adipose tissue for cell-based therapies related to osteoarthritis treatment.

**References**

1. Iyyanki T, Hubenak J, Liu J, Chang EI, Beahm EK, Zhang Q. Harvesting Technique Affects Adipose-Derived Stem Cell Yield. *Aesthet Surg J*. 2015;35(4):467-476. doi:10.1093/asj/sju055

**Study type: *in vitro* study.**

2. Jurgens WJFM, Oedayrajsingh-Varma MJ, Helder MN, et al. Effect of tissue-harvesting site on yield of stem cells derived from adipose tissue: implications for cell-based therapies. *Cell Tissue Res*. 2008;332(3):415-426. doi:10.1007/s00441-007-0555-7

**Study type: *in vitro* study.**

3. Padoin AV, Braga-Silva J, Martins P, et al. Sources of Processed Lipoaspirate Cells: Influence of Donor Site on Cell Concentration: *Plast Reconstr Surg*. 2008;122(2):614-618. doi:10.1097/PRS.0b013e31817d5476

**Study type: *in vitro* study.**

4. Fraser JK, Wulur I, Alfonso Z, Zhu M, Wheeler ES. Differences in stem and progenitor cell yield in different subcutaneous adipose tissue depots. *Cytotherapy*. 2007;9(5):459-467. doi:10.1080/14653240701358460

**Study type: *in vitro* study.**

5. Choudhery MS, Badowski M, Muise A, Pierce J, Harris DT. Subcutaneous Adipose Tissue–Derived Stem Cell Utility Is Independent of Anatomical Harvest Site. *BioResearch Open Access*. 2015;4(1):131-145. doi:10.1089/biores.2014.0059

**Study type: *in vitro* study.**

6. Kim SH, Ha CW, Park YB, Nam E, Lee JE, Lee HJ. Intra-articular injection of mesenchymal stem cells for clinical outcomes and cartilage repair in osteoarthritis of the knee: a meta-analysis of randomized controlled trials. *Arch Orthop Trauma Surg*. 2019;139(7):971-980. doi:10.1007/s00402-019-03140-8

**Study type: Meta-analysis; Level of evidence 2**

7. Kim KI, Kim MS, Kim JH. Intra-articular Injection of Autologous Adipose-Derived Stem Cells or Stromal Vascular Fractions: Are They Effective for Patients With Knee Osteoarthritis? A Systematic Review With Meta-analysis of Randomized Controlled Trials. *Am J Sports Med*. 2023;51(3):837-848. doi:10.1177/03635465211053893

**Study type: Meta-analysis; Level of evidence 1.**

8. Wiggers TG, Winters M, Van den Boom NA, Haisma HJ, Moen MH. Autologous stem cell therapy in knee osteoarthritis: a systematic review of randomised controlled trials. *Br J Sports Med*. 2021;55(20):1161-1169. doi:10.1136/bjsports-2020-103671

9. Qu H, Sun S. Efficacy of mesenchymal stromal cells for the treatment of knee osteoarthritis: a meta-analysis of randomized controlled trials. *J Orthop Surg Res*. 2021;16(1):11. doi:10.1186/s13018-020-02128-0

**Study type: Meta-analysis; Level of evidence 2.**

10. Schreml S, Babilas P, Fruth S, et al. Harvesting human adipose tissue-derived adult stem cells: resection versus liposuction. *Cytotherapy*. 2009;11(7):947-957. doi:10.3109/14653240903204322

**Study type: *in vitro* study.**

11. Oedayrajsingh-Varma MJ, Van Ham SM, Knippenberg M, et al. Adipose tissue-derived mesenchymal stem cell yield and growth characteristics are affected by the tissue-harvesting procedure. *Cytotherapy*. 2006;8(2):166-177. doi:10.1080/14653240600621125

**Study type: *in vitro* study.**

12. Duscher D, Atashroo D, Maan ZN, et al. Ultrasound-Assisted Liposuction Does Not Compromise the Regenerative Potential of Adipose-Derived Stem Cells. *Stem Cells Transl Med*. 2016;5(2):248-257. doi:10.5966/sctm.2015-0064

**Study type: *in vitro* study.**

**QUESTION 18**

**Does harvest location, harvest equipment and number of trajectories matter for bone marrow harvest and preparation?**

**Statement**

Although clinical studies comparing different harvest locations for bone marrow harvesting are lacking, **the posterior and anterior iliac crest have been suggested as the best sites for collecting bone marrow for intra-articular injection because of the highest number of mesenchymal stem/stromal cells (MSCs) available in comparison to other sites**.

Clinical studies directly comparing different harvesting equipment are also lacking. Nevertheless, to improve the quality of bone marrow, that is the lack of peripheral blood contamination, **the consensus group recommends performing multiple puncture sites and different trajectories and gradual advancements to harvest small volumes of bone marrow aspirate** (up to 2-5 ml from each location) from multiple sites with a 10 ml syringe.

Grade of recommendation: C

Rating Score: Mean 8.3±0.7; Median 8 (7-9) Appropriate with strong agreement

*This statement is valid for both POC- and Expanded-CBT*

**Literature summary**

*Harvest location*

In vitro studies focusing on differences in terms of BM-MSCs features are available in literature. A study by Narbona-Carceles et al.^1^ bone marrow was aspirated from the metaphysis of the distal femur, the proximal tibia, and the iliac crest from 20 patients during total knee arthroplasty. The results showed a greater mononuclear cell concentration in the iliac crest compared to the femur and the tibia ( >15 and 6 times higher, respectively). Successful culture rates were highest for BMA from the iliac crest (90%) compared to the femur (71%) and the tibia (47%). No differences were seen in immunophenotypes and all samples showed trilineage differentiation performance. McLain et al.^2^ reported no statistically significant difference in the nucleated cell count or progenitor cells between the vertebra and the iliac crest, whereas Pierini et al.^3^ found that the posterior iliac crest outperformed (1.6 times) the anterior iliac crest, but this difference was only found in CFU-f. A recent study by Pabinger et al.^4^ found no difference in BMA cell count between anterior or posterior harvesting from the iliac crest in a paired-sample design. The cell count, viability and immunophenotype correlated strongly within the same patient when comparing anterior and posterior harvesting. The authors hence suggest applying an anterior approach for safety reasons and practicality.

In clinical practice, Hernigou et al.^5^ highlighted some of the risk factors associated with posterior and anterior iliac crest collection. Thin sectors were at higher risks compared to thicker sections. The anterior approach gave risk of the trocar reaching the external iliac artery. The posterior sections were at risk of sciatic nerve and gluteal vessel damage when the trocar was pushed > 6 cm into the posterior iliac crest. Efforts should hence be made to direct the trocar away from neural and vascular structures and towards zones with high bone marrow stock. Collection of bone marrow from the ventral part of the iliac crest can be done in the supine position, whereas for collection of bone marrow from the spina iliaca posterior the patients have to be in the prone or lateral position^4^.

Anatomical harvest differences are primarily related to number of cells of interest and not to performance of the cells. Multiple locations can thus be considered depending on the number of cells needed for a given procedure. Although more studies are warranted in this regard, the general finding is that the iliac crest is the most suitable location for harvest of BM for treatment of osteoarthritis.

*Harvest equipment*

It is estimated that only 40% of BMAC studies describe the applied bone marrow aspiration technique sufficiently for reproducibility^4^. The various harvest equipment’s for harvest of BMA differ significantly in their technical features, anticoagulants applied, aspirate volume, and later centrifugation parameters and methods to describe BMA quantity and biologic potency.

A review by Gaul et al.^6^ compared commercially available point-of-care devices for harvest and isolation of BMA. A major difference is the method used to extract the buffy coat containing the BMAC of interest. Some fo these devices are fully automated, contains an internal centrifuge, and delivers the BMAC ready to use in a syringe. The other devices rely on manual extraction of the buffy coat and an external centrifuge and syringe preparation. No publications could be identified to determine the likelihood of contamination using the different devices. Studies suggest that “fast” and continuous aspiration of bone marrow with high differential pressure (e.g. using a back-lock/VacLok^TM^ syringe) together with multiple advancements, result in more stem cells and a better product ^6^. Bone marrow aspiration needles included in the kits are of different sizes (11-15G) and with various number of holes. Pabinger et al.^4^ found differences in the use of needles from different companies. A study by Brestoff et al.^7^ reported that a single-bevel bone marrow needle was less likely to produce hemodilute aspirates compared to a triple-bevel needle.

As centrifugation has previously been shown to affect stem cells^8^, recent studies report harvest of bone marrow aspirate using centrifuge-free methods by different aspiration techniques^9,10^. A study by Marx et al.^9^ compared three harvesting devices in a non-paired study design, highlighting differences in terms total nucleated cell counts, CFU-f values. The forward aspiration method is said by the authors to reduce the dilution from peripheral blood, but the total cellular immunophenotypes were not assessed. The flexible needle is said by the authors to facilitate targeting of lining cells with high stem/progenitor cell numbers along the inner cortex, but was not specifically determined in the current study. The compared methods used different types and concentrations of anticoagulants and bone marrow aspiration volumes and these may have influenced the results ^9^. Anticoagulants have been shown to affect BMA differently^11^. Heparin sodium provided greater CFU-f from bone marrow aspirates compared to sodium citrate, and differences were also reported in the cytokine profile^11^.

Hence, a recommendation of a single device is not possible, as the variations are not controlled for in the different study set ups. As mentioned in a review by Gaul et al.^6^ a standardized reporting method is needed for valid comparisons, together with clinical outcomes to establish the true efficacy of the various harvest equipments.

*Number of trajectories*

Multiple puncture sites or gradual advancements to harvest small volumes of bone marrow aspirate from multiple sites with a 10 mL syringe are generally recommended^12^. The multiple trajectories typically sum up to a minimum volume of 10 mL bone marrow aspirate, which has shown promising results when treating knee osteoarthritis^10,13^. The highest concentration of stem cells has been found in the first small volumes of bone marrow aspirates^14,15^. The collected volume should therefore be considered. A rotational concept, with small bone marrow aspiration volumes (preferably 1-2 mL per site) and the use of a 10 mL syringe was also reported by Hernigou et al.^12^ to provide greater concentration of stem cells and less peripheral blood dilution compared to using a 50 mL syringe when harvesting from the iliac crest.

A study by Muschler et al.^15^ compared 1, 2, and 4 mL bone marrow aspirates from the anterior iliac crest and found an increase in peripheral blood with increasing volume and a 50% reduction in alkaline phosphatase-positive CFU-f when upscaling. The authors recommend a maximum of 2 mL bone marrow aspirate from one site. Similarly to Hernigou et al.^12^ and Batinic^15^. In 2022, Pabinger et al. compared a reorientation technique (2x2 mL bone marrow aspiration per site using reorientation after the first 2 mL) from the iliac crest to a conventional technique (multiple puncture sites where large volumes are harvested from the same site – single orientation). Using the reorientation technique, the authors found that it led led to higher viability, leucocytes and CD34+ cells compared to the conventional technique^4^.

Batinic et al.^14^ make a general recommendation of multiple aspirates with small volumes of bone marrow aspirates taken from bone puncture sites as distant as possible. Gaul et al.^6^ recommend multiple advancements during the bone marrow aspiration. Pabinger et al. make a recommendation of the two combined^14^.

**References**

1. Narbona-Carceles J, Vaquero J, Suárez-Sancho S,, Forriol F, Fernández-Santos ME. Bone marrow mesenchymal stem cell aspirates from alternative sources Is the knee as good as the iliac crest? *Injury*. 2014;45:S42-S47. doi:10.1016/S0020-1383(14)70009-9

**Study type: *in vitro* study.**

1. McLain RF, Fleming JE, Boehm CA, Muschler GF. Aspiration of Osteoprogenitor Cells for Augmenting Spinal Fusion: Comparison of Progenitor Cell Concentrations from the Vertebral Body and Iliac Crest. *J Bone Jt Surg*. 2005;87(12):2655-2661. doi:10.2106/JBJS.E.00230

**Study type: *in vitro* study.**

1. Pierini M, Di Bella C, Dozza B, et al. The Posterior Iliac Crest Outperforms the Anterior Iliac Crest When Obtaining Mesenchymal Stem Cells from Bone Marrow: *J Bone Jt Surg-Am Vol*. 2013;95(12):1101-1107. doi:10.2106/JBJS.L.00429

**Study type: *in vitro* study.**

1. Pabinger C, Dammerer D, Lothaller H, Kobinia GS. Reorientation technique has benefits in bone marrow aspiration of stem cells. *Sci Rep*. 2022;12(1):11637. doi:10.1038/s41598-022-15019-7

**Study type: *in vitro* study.**

1. Hernigou J, Picard L, Alves A, Silvera J, Homma Y, Hernigou P. Understanding bone safety zones during bone marrow aspiration from the iliac crest: the sector rule. *Int Orthop*. 2014;38(11):2377-2384. doi:10.1007/s00264-014-2343-9

**Study Type: Clinical Trial; Level of evidence 4.**

1. Gaul F, Bugbee WD, Hoenecke HR, D’Lima DD. A Review of Commercially Available Point-of-Care Devices to Concentrate Bone Marrow for the Treatment of Osteoarthritis and Focal Cartilage Lesions. *Cartilage*. 2019;10(4):387-394. doi:10.1177/1947603518768080

**Study Type: Narrative Review; Level of evidence 4.**

1. Brestoff JR, Toland A, Afaneh K, et al. Bone Marrow Biopsy Needle Type Affects Core Biopsy Specimen Length and Quality and Aspirate Hemodilution. *Am J Clin Pathol*. 2019;151(2):185-193. doi:10.1093/ajcp/aqy126

**Study type: *in vitro* study.**

1. Conde-Green A, Baptista LS, De Amorin NFG, et al. Effects of Centrifugation on Cell Composition and Viability of Aspirated Adipose Tissue Processed for Transplantation. *Aesthet Surg J*. 2010;30(2):249-255. doi:10.1177/1090820X10369512

**Study type: *in vitro* study.**

1. Marx RE, Amailuk P, Patel N, Ledoux A, Stanbouly D. FlexMetric bone marrow aspirator yields laboratory and clinically improved results from mesenchymal stem and progenitor cells without centrifugation. *J Tissue Eng Regen Med*. 2022;16(11):1047-1057. doi:10.1002/term.3348

**Study Type: RCT; Level of evidence 3.**

1. Viganò M, Ragni E, Di Matteo B, et al. A single step, centrifuge-free method to harvest bone marrow highly concentrated in mesenchymal stem cells: results of a pilot trial. *Int Orthop*. 2022;46(2):391-400. doi:10.1007/s00264-021-05243-7

**Study Type: Pilot Trial; Level of evidence 4.**

1. Dregalla RC, Herrera JA, Koldewyn LS, Donner EJ. The Choice of Anticoagulant Influences the Characteristics of Bone Marrow Aspirate Concentrate and Mesenchymal Stem Cell Bioactivity In Vitro. Mezey E, ed. *Stem Cells Int*. 2022;2022:1-12. doi:10.1155/2022/8259888

**Study type: *in vitro* study.**

1. Hernigou P, Homma Y, Flouzat Lachaniette CH, et al. Benefits of small volume and small syringe for bone marrow aspirations of mesenchymal stem cells. *Int Orthop*. 2013;37(11):2279-2287. doi:10.1007/s00264-013-2017-z

**Study type: *in vitro* study.**

1. Kuebler D, Schnee A, Moore L, et al. Short-Term Efficacy of Using a Novel Low-Volume Bone Marrow Aspiration Technique to Treat Knee Osteoarthritis: A Retrospective Cohort Study. Qian L, ed. *Stem Cells Int*. 2022;2022:1-7. doi:10.1155/2022/5394441

**Study Type: Retrospective Cohort Study; Level of evidence 4.**

1. Batinić D, Marusić M, Pavletić Z, et al. Relationship between differing volumes of bone marrow aspirates and their cellular composition. *Bone Marrow Transplant*. 1990;6(2):103-107.

**Study type: *in vitro* study.**

1. Muschler GF, Boehm C, Easley K. Aspiration to Obtain Osteoblast Progenitor Cells from Human Bone Marrow: The Influence of Aspiration Volume*. *J Bone Joint Surg Am*. 1997;79(11):1699-1709. doi:10.2106/00004623-199711000-00012

**Study type: *in vitro* study.**

**SECTION 3**

**QUESTION 19**

**When using expanded mesenchymal stem/stromal cells (MSCs), what is the optimal/most appropriate number of cells to inject?**

**Statement**

The majority of available dose-response studies reported the use of <100 × 10^6^ mesenchymal stem/stromal cells (MSCs). However, due to the lack of stringency and high heterogeneity in the design of the available studies and due to the absence of a clear correlation between cell numbers and clinical outcomes, as well as various cell numbers in different studies, currently no consensus exists about the most appropriate number of expanded MSCs to inject in the treatment of knee OA. **The consensus group concludes that defining the optimal MSC number for the management of knee OA is complex and includes many variables, and, therefore, currently optimal cell ranges for the treatment of knee OA cannot be defined.**

Grade of recommendation: C

Rating Score: Mean 8.4±1.0; Median 9 (5-9) Appropriate with relative agreement

**Literature summary**

A wide range of MSC doses varying from approximately 1×10^6^ to 200×10^6^ (mean value = 38 × 10^6^ cells) can be found in the literature in the treatment of knee osteoarthritis^1^. This is also reported in a meta-analysis by Wang^2^ who investigated cell doses in 120 pre-clinical and clinical studies using MSCs for the treatment of knee OA. Due to lack of stringency and high heterogeneity in the design of the available studies and to contradicting results no consensus exists about the most appropriate number of expanded mesenchymal stem cells to inject in the treatment of knee osteoarthritis^2,3^.

Selected human studies

A study by Pers^4^ investigated intraarticular injection with three different doses of expanded autologous AT-MSCs (2, 10, and 50 × 10^6^) in 18 patients, six in each group in the treatment of knee OA.^7^ No between group comparison was performed. Significant improvement in WOMAC was only detected for patients treated with the lowest dose.

Lamo-Espinosa et al.^5,6^ investigated intraarticular injection with a combination of HA and autologous BM-MSCs in the treatment of knee OA. 30 patients were randomized in three groups: HA, HA + 10 × 10^6^ BM‑MSCs, HA + 100 × 10^6^. Only the latter group was significantly better than HA at 12^5^ and 24 months^5,6^.

A study by Song^7^ investigated the efficacy of three intra-articular injections with autologous AT-MSCs in 18 patients divided into three cell dose groups (10, 20 and 50x10^6^). After 8 months, all groups showed a significant improvement over baseline, with the middle one having the best result.

Two studies by Jo^8,9^ investigated intra-articular injection of AT-MSCs for knee OA in 18 patients divided into three dose groups (10, 50 and 80x10^6^ according to Jo^8^ or 100x10^6^ according to Jo^9^). Improved knee function was seen for up to 2 years regardless of the cell dosage.

A study by Chahal^10^ investigated a single intra-articular injection of BM-MSCs in 12 patients divided into three dose groups (1, 10 and 100x10^6^ cells), with the latter having the highest number of patients achieving the MCID for PROMs.

Lu et al.^11^ injected knee OA patients with two doses of **allogeneic** AD-MSCs (AlloJoin®) at different concentrations (10, 20, or 50 x10^6^ cells, 3 weeks interval) with no major safety issues or adverse events at 48 weeks. Clinical improvements from baseline were measured in all the groups, whereas MRI assessments showed slight improvements in the low-dose group only.

**References**

1. Gupta PK, Thej C. Mesenchymal stromal cells for the treatment of osteoarthritis of knee joint: context and perspective. *Ann Transl Med*. 2019;7(S6):S179-S179. doi:10.21037/atm.2019.07.54

**Study type: Editorial; Level of evidence 5.**

2. Wang G, Xing D, Liu W, et al. Preclinical studies and clinical trials on mesenchymal stem cell therapy for knee osteoarthritis: A systematic review on models and cell doses. *Int J of Rheum Dis*. 2022;25(5):532-562. doi:10.1111/1756-185X.14306

**Study type: Systematic review; Level of evidence 3.**

3. Wiggers TG, Winters M, Van Den Boom NA, Haisma HJ, Moen MH. Autologous stem cell therapy in knee osteoarthritis: a systematic review of randomised controlled trials. *Br J Sports Med*. 2021;55(20):1161-1169. doi:10.1136/bjsports-2020-103671

**Study type: Meta-analysis; Level of evidence 2.**

4. Pers YM, Rackwitz L, Ferreira R, et al. Adipose Mesenchymal Stromal Cell-Based Therapy for Severe Osteoarthritis of the Knee: A Phase I Dose-Escalation Trial. *Stem Cells Translational Medicine*. 2016;5(7):847-856. doi:10.5966/sctm.2015-0245

**Study type: Uncontrolled clinical trial; Level of evidence 3.**

5. Lamo-Espinosa JM, Mora G, Blanco JF, et al. Intra-articular injection of two different doses of autologous bone marrow mesenchymal stem cells versus hyaluronic acid in the treatment of knee osteoarthritis: multicenter randomized controlled clinical trial (phase I/II). *J Transl Med*. 2016;14(1):246. doi:10.1186/s12967-016-0998-2

**Study type: RCT; Level of evidence 1.**

6. Lamo-Espinosa JM, Mora G, Blanco JF, et al. Intra-articular injection of two different doses of autologous bone marrow mesenchymal stem cells versus hyaluronic acid in the treatment of knee osteoarthritis: long-term follow up of a multicenter randomized controlled clinical trial (phase I/II). *J Transl Med*. 2018;16(1):213. doi:10.1186/s12967-018-1591-7

**Study type: RCT; Level of evidence 1.**

7. Song Y, Du H, Dai C, et al. Human adipose-derived mesenchymal stem cells for osteoarthritis: a pilot study with long-term follow-up and repeated injections. *Regenerative Medicine*. 2018;13(3):295-307. doi:10.2217/rme-2017-0152

**Study Type: Clinical trial; Level of evidence 3.**

8. Jo CH, Lee YG, Shin WH, et al. Intra‐Articular Injection of Mesenchymal Stem Cells for the Treatment of Osteoarthritis of the Knee: A Proof‐of‐Concept Clinical Trial. *Stem Cells*. 2014;32(5):1254-1266. doi:10.1002/stem.1634

**Study type: Proof-of-concept Clinical Trial; Level of evidence 4.**

9. Jo CH, Chai JW, Jeong EC, et al. Intra-articular Injection of Mesenchymal Stem Cells for the Treatment of Osteoarthritis of the Knee: A 2-Year Follow-up Study. *Am J Sports Med*. 2017;45(12):2774-2783. doi:10.1177/0363546517716641

**Study type: Prospective Cohort Study; Level of evidence 3.**

10. Chahal J, Gómez-Aristizábal A, Shestopaloff K, et al. Bone Marrow Mesenchymal Stromal Cell Treatment in Patients with Osteoarthritis Results in Overall Improvement in Pain and Symptoms and Reduces Synovial Inflammation. *Stem Cells Translational Medicine*. 2019;8(8):746-757. doi:10.1002/sctm.18-0183

**Study type: Uncontrolled Clinical Trial; Level of evidence 3.**

11. Lu L, Dai C, Du H, et al. Intra-articular injections of allogeneic human adipose-derived mesenchymal progenitor cells in patients with symptomatic bilateral knee osteoarthritis: a Phase I pilot study. *Regenerative Medicine*. 2020;15(5):1625-1636. doi:10.2217/rme-2019-0106

**Study type: RCT; Level of evidence 2.**

**QUESTION 20**

**For CBT Injections in knee OA – is one injection sufficient per treatment cycle?**

**Statement**

Current literature is scarce with regards to the optimal number of CBT injections per treatment cycle for the management of knee OA. To date, no study involving autologous POC-CBT includes more than one injection protocol, whereas a few studies using expanded mesenchymal stem/stromal cells (MSCs) reported the outcomes of multiple injections in a short interval. Although studies using expanded cells with more than one-injection protocols have shown to provide clinical benefit, there is a lack of sufficient data to support multiple injection protocols over single-injection protocols and, therefore, **the consensus group cannot recommend one protocol over the other for either POC-CBT or expanded-CBT for the management of knee OA.**

Grade of recommendation: C

Rating Score: Mean 8.4±0.8; Median 8 (6-9) Appropriate with relative agreement

*This statement is valid for both POC- and Expanded-CBT*

**Literature summary**

There is scarce evidence about the number of injections of the MSCs for the knee OA. To date no study involving autologous CBT prepared at the point of care includes more than one injection, whereas a few studies about expanded MSCs show the outcomes of multiple injections per treatment cycle.

A level 1 RCT^1^ was conducted to evaluate the efficacy of either one or two injections of autologous AT-MSCs on pain, function and disease modification in knee OA. Thirty patients with symptomatic knee OA were randomized to receive either a single injection (100 × 10^6^ AT-MSCs) or two injections (100 × 10^6^ AT-MSCs at baseline and 6 months) or to continue conservative management. No serious adverse events were observed, there was no difference between groups in bone marrow lesions, synovitis, meniscus pathology and popliteal cysts on MRI assessment. Both treatment groups receiving AT-MSCs showing clinically significant pain and functional improvement at 12 months. Sctructural MRI analysis using indicated modification of disease progression. Authors suggested that more consistent OA control was achieved via two injections compared to one injection. However, the distance between the two infiltrations can hardly be attributed to a single treatment cycle. The Authors also reported that a third treatment group receiving five injections of 40 × 10^6^ AT-MSCs at baseline, 1, 2, 3 and 6 months was initially meant to be included in the study. However, this was not performed because of the reproducible moderate adverse events observed in another concomitant study, although no further explanation is given.

Another level I RCT^2^ compared a repeated injection of UC-MSCs to a single injection and an injection of HA. Patients with symptomatic knee OA were randomized in three groups to receive either HA at baseline and at 6 months, a single-dose of allogeneic UC-MSCs (20× 10^6^) at baseline, or repeated allogeneic UC-MSC doses (20× 10^6^) at baseline and at 6 months. At the 12 months follow up, WOMAC reached significantly lower levels of pain (pain improvement) in the repeated allogeneic UC-MSC group compared with the HA group, while the single dose allogeneic UC-MSC did not provide significant pain improvement compared to HA. No differences in MRI scores were detected between the groups.

In a pilot-study^3^, two repeated injections of 50x10^6^ autologous adipose-derived MSC (3^rd^ and 6^th^ week after liposuction) lead to significant pain and function improvement in OA knees compared to two injections of 10×10^6^ and 20×10^6^. The third injection 48 weeks after led to increased cartilage volume improvement from week 48^th^ to week 96^th^, compared to the period of baseline to 48^th^ week. After the first two injections, decreasing tendency was observed and the repeated injection led to benefits. There was no group that received a single injection in this study.

Another study^4^ used two injections of allogeneic AT-MSCs (50× 10^6^, 15 days apart) which showed promising results, but there was no comparison with single injection. They were compared with HA and showed better WOMAC score and more cartilage volume after 12 months.

An observational study^5^ also used a two-injection protocol of autologous BM-MSCs given 4 weeks apart (61× 10^6^ in two injections). Knee cartilage thickness improved significantly compared to baseline, but no control against one single injection is available.

In terms of safety, Ao et al.^6^ suggested that repeated intra-articular injection of allogeneic UC-MSCs were safe in treating OA. In this study patients received 4 intra-articular injections once a week and although some adverse reactions emerged during the research, all of them were transient and did not harm the patient.

*For additional information on safety of multiple injections, please refer to Question 8*

**References**

1. Freitag J, Bates D, Wickham J, et al. Adipose-derived mesenchymal stem cell therapy in the treatment of knee osteoarthritis: a randomized controlled trial. *Regen Med*. 2019;14(3):213-230. doi:10.2217/rme-2018-0161

**Study type: RCT; Level of evidence 2.**

2. Matas J, Orrego M, Amenabar D, et al. Umbilical Cord-Derived Mesenchymal Stromal Cells (MSCs) for Knee Osteoarthritis: Repeated MSC Dosing Is Superior to a Single MSC Dose and to Hyaluronic Acid in a Controlled Randomized Phase I/II Trial. *Stem Cells Translational Medicine*. 2019;8(3):215-224. doi:10.1002/sctm.18-0053

**Study type: RCT; Level of evidence 2.**

3. Song Y, Du H, Dai C, et al. Human adipose-derived mesenchymal stem cells for osteoarthritis: a pilot study with long-term follow-up and repeated injections. *Regenerative Medicine*. 2018;13(3):295-307. doi:10.2217/rme-2017-0152

**Study Type: Clinical trial; Level of evidence 3.**

4. Lu L, Dai C, Zhang Z, et al. Treatment of knee osteoarthritis with intra-articular injection of autologous adipose-derived mesenchymal progenitor cells: a prospective, randomized, double-blind, active-controlled, phase IIb clinical trial. *Stem Cell Res Ther*. 2019;10(1):143. doi:10.1186/s13287-019-1248-3

**Study type: RCT; Level of evidence 1.**

5. Al-Najar M, Khalil H, Al-Ajlouni J, et al. Intra-articular injection of expanded autologous bone marrow mesenchymal cells in moderate and severe knee osteoarthritis is safe: a phase I/II study. *J Orthop Surg Res*. 2017;12(1):190. doi:10.1186/s13018-017-0689-6

**Study type: Prospective uncontrolled clinical trial; Level of evidence 3.**

6. Ao Y, Duan J, Xiong N, et al. Repeated intra-articular injections of umbilical cord-derived mesenchymal stem cells for knee osteoarthritis: a phase I, single-arm study. *BMC Musculoskelet Disord*. 2023;24(1):488. doi:10.1186/s12891-023-06555-y

**Study type: Case series; Level of evidence 4.**

**QUESTION 21**

**Are fasting or dietary restrictions recommended before CBT use? Could any other patient behavior affect the treatment?**

**Statement**

The literature lacks clinical data regarding the direct impact of diet and fasting or other lifestyle recommendations on the therapeutic effects of CBT. However, since basic science literature reports negative effects of these behaviors on mesenchymal stem/stromal cells (MSC) performances, the consensus group acknowledges the importance of adopting some dietary and life restrictions (including alcohol consumption and smoking habit) a few weeks before the treatment in case of autologous treatment to maximize the efficacy. Moreover, given the presence of peripheral blood contamination in most of the POC-CBT (mainly cone marrow concentrate and stromal vascular fraction), similar recommendations to those for the use of platelet rich plasma should be followed. **The consensus group, therefore, recommends patients to avoid high-fat foods for at least 24 hours prior to a CBT treatment.**

Grade of recommendation: D

Rating Score: Mean 8.1±1.1; Median 8 (5-9) Appropriate with relative agreement

*This statement is valid for both POC- and Expanded-CBT*

**Literature summary:**

The current scientific literature does not include clinical studies evaluating the influence of diet and other types of patient behavior on treatments based on cell therapy. Therefore, the hypothetical effect of these variables is deduced from studies that analyze the aforementioned factors on cells and cellular niches or the overall effects of dietary modification on the alterations in glucose metabolism and on systemic inflammatory state.

While it is obvious that a few hours of fasting cannot instantly modify the metabolic state of adipose tissue or bone marrow, fasting and/or dietaty restrictions has been instead reported to have an immediate effect of blood composition/inflammatory state (see The use of injectable Orthobiologics for knee osteoarthritis: a formal ESSKA consensus, Part 1 - Blood-derived Products (PRP), Question 25).

Since most of the CBT preparations are contaminated by blood (especially BMAC but also SVF), as well as some surgeons inject CBT products with clood-derived products such as PRP, dietary recommendations before the CBT treatment seem reasonable too.

For adipose tissue these recommendations can include both fasting right before the procedure or a helthier diet style some weeks before it. Infact, studies on AT-MSCs isolated from obese patients showed that their immunomodulatory effect was altered favoring a pro-inflammatory environment^1^. The different findings include inflammatory cytokine production and monocyte activation^2^, less effective in suppressing lymphocyte proliferation and activating M2 macrophages^3^, decreased anti-inflammatory effect^4^, upregulated expression of pro-inflammatory genes^5^, potentiation of M1 macrophages and increased TNF-α levels^6^, and lower angiogenic potential and greater adipogenic differentiation^7^. This alteration in the cellular differentiation of AT-MSCs from obese individuals was also observed in *in vivo* studies in which a decrease in chondrogenic capacity was also observed^8^. It was demonstrated that the daily cycles of fasting and feeding induce changes on adipose tissue, specifically in lipid level, glucose metabolism with decrease of insulin release and a global improvement in the quality of adipose tissue especially in terms of inflammatory profile^9^. Another nutritional strategy that patients could employ to improve the chronic low-grade inflammation is represented by plant-based dietary interventions, able to reduce inflammatory biomarkers such as CRP, IL-6, sICAM^10^.

Concerning bone marrow, while it metabolic state seems to be less related to diet style, the contamination with blood of BMAC products make it necessary to pay attention to dietary restrictions both right before the procedure (i.e fasting or a non-fatty diet) and a few time before it. Obesity results in a low-grade inflammatory state throughout the body which is also affecting the bone marrow, with changes in its composition^11^, in turn affecting characteristic of BM-MSCs^12^, such as their mobilization and secretion of molecules^11^. While red blood cells and white blood cells of bone marrow are increased, BM-MSCs from obese patients presented a more adipogenic profile and alterations whose differentiation into osteoblasts and chondrocytes is diminished^13,14^. In addition, it was also observed an increase in the intracellular reactive oxygen species leading to cellular senescence^13,15^, and elevated expression of endoplasmic reticulum stress–related genes^15^.

Other patients’ habits, such as alcohol consumption ad smoking may affect the cell performances. However, the complete lack of clinical literature on this topic may it difficult to draft any recommendations. It is known that alcohol favors adipogenesis while impeding osteogenic and chondrogenic differentiation, and increases cellular senescence of MSCs^16,17^ through deregulation of the Wnt/β-catenin signaling pathway. This effect may persist for up to two weeks and it may therefore be reasonable to suggest that the patient stop drinking alcohol a few weeks before the orthobiologic treatment. Likewise, smoking is also able to impair chondrogenic differentiation.

**References**

1. Mahmoud M, Abdel-Rasheed M. Influence of type 2 diabetes and obesity on adipose mesenchymal stem/stromal cell immunoregulation. *Cell Tissue Res*. 2023;394(1):33-53. doi:10.1007/s00441-023-03801-6

**Study type: Narrative Review; Level of evidence 4.**

2. Eljaafari A, Robert M, Chehimi M, et al. adipose tissue–derived stem cells from obese subjects contribute to inflammation and reduced insulin response in adipocytes through differential regulation of the Th1/Th17 balance and monocyte activation. *Diabetes*. 2015;64(7):2477-2488. doi:10.2337/db15-0162

**Study type: *In vitro* study**

3. Serena C, Keiran N, Ceperuelo-Mallafre V, et al. Obesity and type 2 diabetes alters the immune properties of human adipose derived stem cells. *Stem Cells*. 2016;34(10):2559-2573. doi:10.1002/stem.2429

**Study type: *In vitro* study**

4. Strong AL, Bowles AC, Wise RM, et al. Human adipose stromal/stem cells from obese donors show reduced efficacy in halting disease progression in the experimental autoimmune encephalomyelitis model of multiple sclerosis. *Stem Cells*. 2016;34(3):614-626. doi:10.1002/stem.2272

**Study type: *In vitro* study**

5. Harrison MAA, Wise RM, Benjamin BP, Hochreiner EM, Mohiuddin OA, Bunnell BA. Adipose-derived stem cells from obese donors polarize macrophages and microglia toward a pro-inflammatory phenotype. *Cells*. 2020;10(1):26. doi:10.3390/cells10010026

**Study type: *In vitro* study**

6. Zhu X, Klomjit N, Conley SM, et al. Impaired immunomodulatory capacity in adipose tissue‐derived mesenchymal stem/stromal cells isolated from obese patients. *J Cellular Molecular Medi*. 2021;25(18):9051-9059. doi:10.1111/jcmm.16869

**Study type: *In vitro* study**

7. Juntunen M, Heinonen S, Huhtala H, et al. Evaluation of the effect of donor weight on adipose stromal/stem cell characteristics by using weight-discordant monozygotic twin pairs. *Stem Cell Res Ther*. 2021;12(1):516. doi:10.1186/s13287-021-02587-0

**Study type: *In vitro* study**

8. Wu CL, Diekman BO, Jain D, Guilak F. Diet-induced obesity alters the differentiation potential of stem cells isolated from bone marrow, adipose tissue and infrapatellar fat pad: the effects of free fatty acids. *Int J Obes*. 2013;37(8):1079-1087. doi:10.1038/ijo.2012.171

**Study type: *In vitro* study**

9. Atakan MM, Koşar ŞN, Güzel Y, Tin HT, Yan X. The Role of Exercise, Diet, and Cytokines in Preventing Obesity and Improving Adipose Tissue. *Nutrients*. 2021;13(5):1459. doi:10.3390/nu13051459

**Study type: Narrative Review; Level of evidence 4.**

10. Eichelmann F, Schwingshackl L, Fedirko V, Aleksandrova K. Effect of plant‐based diets on obesity‐related inflammatory profiles: a systematic review and meta‐analysis of intervention trials. *Obesity Reviews*. 2016;17(11):1067-1079. doi:10.1111/obr.12439

**Study type: Systematic review and meta‐analysis; Level of evidence 3.**

11. Benova A, Tencerova M. Obesity-induced changes in bone marrow homeostasis. *Front Endocrinol*. 2020;11:294. doi:10.3389/fendo.2020.00294

**Study type: Narrative Review; Level of evidence 4.**

12. Zong Q, Bundkirchen K, Neunaber C, Noack S. Are the Properties of Bone Marrow-Derived Mesenchymal Stem Cells Influenced by Overweight and Obesity? *IJMS*. 2023;24(5):4831. doi:10.3390/ijms24054831

**Study type: Narrative Review; Level of evidence 4.**

13. Tencerova M, Frost M, Figeac F, et al. Obesity-associated hypermetabolism and accelerated senescence of bone marrow stromal stem cells suggest a potential mechanism for bone fragility. *Cell Reports*. 2019;27(7):2050-2062.e6. doi:10.1016/j.celrep.2019.04.066

**Study type: *In vitro* study**

14. Di Bernardo G, Messina G, Capasso S, et al. Sera of overweight people promote in vitro adipocyte differentiation of bone marrow stromal cells. *Stem Cell Res Ther*. 2014;5(1):4. doi:10.1186/scrt393

**Study type: *In vitro* study**

15. Ulum B, Teker HT, Sarikaya A, et al. Bone marrow mesenchymal stem cell donors with a high body mass index display elevated endoplasmic reticulum stress and are functionally impaired. *Journal Cellular Physiology*. 2018;233(11):8429-8436. doi:10.1002/jcp.26804

**Study type: *In vitro* study**

16. Di Rocco G, Baldari S, Pani G, Toietta G. Stem cells under the influence of alcohol: effects of ethanol consumption on stem/progenitor cells. *Cell Mol Life Sci*. 2019;76(2):231-244. doi:10.1007/s00018-018-2931-8

**Study type: Narrative Review; Level of evidence 4.**

17. Chen X, Li M, Yan J, et al. Alcohol induces cellular senescence and impairs osteogenic potential in bone marrow-derived mesenchymal stem cells. *Alcohol and Alcoholism*. 2017;52(3):289-297. doi:10.1093/alcalc/agx006

**Study type: *In vitro* study**

**QUESTION 22**

**Is Antibiotics administration recommended around CBT use?**

**Statement**

Evidence on antibiotics administration around CBT use is lacking. **Therefore, the consensus group does not recommend the routine use of antibiotics around CBT use.** However, unlike other injectable products for the knee joint, autologous CBT preparation process involves tissue harvesting (mainly but not only fat or bone-marrow) and therefore some degree of infectious risk should be taken into consideration. To reduce the infectious risk, **the consensus group recommends performing CBT procedures in an appropriate and dedicated environment** (i.e. sterile office area, operating theater or similar environments). Nevertheless, the consensus group suggests taking a cautious approach in specific cases and consider the administration of antibiotics in populations with higher risk factors for infections, such as diabetics, heavy smokers, previous joint infections, or wound complications.

Grade of recommendation: D

Rating Score: Mean 8.4±0.8; Median 9 (6-9) Appropriate with relative agreement

*This statement is valid for both POC- and Expanded-CBT*

**Literature summary:**

In literature, no data could be found about prophylactic use of antibiotics for CBT treatment. It is known from the literature that CBT possess certain antimicrobial properties^1,2^.

There are many studies that investigated antibiotic and antimycotic prophylaxis of people receiving hematopoietic stem cells, but since these are usually systemic diseases and patients with severely damaged immune system, interpolation of the results to the field of OA treatment would not be reasonable^3–5^. However, there is concern of possible infection with use of CBT products, with a study reporting infections being 50% of the rare adverse events following CBT injections^6^, although neither the total number of the infections nor the total number of adverse events are reported, impeding therefore to appreciate the real weight of these findings. Systemic use of antibiotics during CBT treatment cannot be suggested on evidence-based data, but neither it can be said to be contraproductive in terms of CBT efficacy, although the general negative effects of antibiotics overuse is widely known.

Mixing the antibiotic directly with MSCs could have deleterious effects because certain antibiotics alter the proliferation of cells. They can also affect the differentiation process, i.e. promote osteogenesis and adipogenesis, which are not favorable lineage goals^7^.

**References**

1. Yagi H, Chen AF, Hirsch D, et al. Antimicrobial activity of mesenchymal stem cells against Staphylococcus aureus. *Stem Cell Res Ther*. 2020;11(1):293. doi:10.1186/s13287-020-01807-3

**Study type: *in vitro* study**

2. Chow L, Johnson V, Impastato R, Coy J, Strumpf A, Dow S. Antibacterial activity of human mesenchymal stem cells mediated directly by constitutively secreted factors and indirectly by activation of innate immune effector cells. *Stem Cells Transl Med*. 2020;9(2):235-249. doi:10.1002/sctm.19-0092

**Study type: *in vitro* and vivo study**

3. Pérez-Simón JA, García-Escobar I, Martinez J, et al. Antibiotic prophylaxis with meropenem after allogeneic stem cell transplantation. *Bone Marrow Transplant*. 2004;33(2):183-187. doi:10.1038/sj.bmt.1704335

**Study type: Uncontrolled, non-randomized, clinical trial; Level of Evidence 4**

4. Jenq RR, van den Brink MRM. Antibiotic prophylaxis in allogeneic stem cell transplantation-what is the correct choice? *Bone Marrow Transplant*. 2016;51(8):1071-1072. doi:10.1038/bmt.2016.144

**Study type: Non-randomized clinical trial compared to controls retrospectively; Level of evidence 4**

5. Kimura S ichi, Akahoshi Y, Nakano H, et al. Antibiotic prophylaxis in hematopoietic stem cell transplantation. A meta-analysis of randomized controlled trials. *J Infect*. 2014;69(1):13-25. doi:10.1016/j.jinf.2014.02.013

**Study type: Systematic Review and meta-analysis; Level of evidence 2**

6. Elia CA, Tamborini M, Rasile M, et al. Intracerebral Injection of Extracellular Vesicles from Mesenchymal Stem Cells Exerts Reduced Aβ Plaque Burden in Early Stages of a Preclinical Model of Alzheimer’s Disease. *Cells*. 2019;8(9). doi:10.3390/cells8091059

**Study type: Case series; Level of evidence 4**

7. Skubis A, Gola J, Sikora B, et al. Impact of Antibiotics on the Proliferation and Differentiation of Human Adipose-Derived Mesenchymal Stem Cells. *Int J Mol Sci*. 2017;18(12):2522. doi:10.3390/ijms18122522

**Study type: *in vitro* study**

**Question 23**

**Is there any clinical benefit combining platelet rich plasma (PRP) to CBT?**

**Statement**

Current pre-clinical and clinical literature suggest some potential benefits combining platelet rich plasma (PRP) with cell-based products, with the majority of studies focusing on culture-expanded cells. However, evidence is still lacking regarding the clear benefits of using these products in combination over using CBT alone. **Therefore, based on current evidence, the consensus group does not see clear advantages from combining PRP to CBT products for knee OA and does not recommend a combined treatment.**

Grade of recommendation: C

Rating Score: Mean 8.4±0.7; Median 9 (7-9) Appropriate with strong agreement

*This statement is valid for both POC- and Expanded-CBT*

**Literature summary:**

A rationale that PRP can be a beneficial adjunct to MSCs exists because of their dissimilar biologic action. One of the most interesting ideas about MSCs and PRP synergy is that PRP alters the hostile environment in the joint caused by osteoarthritis and leads to longer survival of the cells. It provides growth factors to help better proliferation of MSCs and, on the other hand, acts as scaffold for attaching cells to the site of cartilage damage. Most of the studies in this topic are not clinical.

*In vitro and pre-clinical findings*

An in vitro study found that adding PRP to a culture of MSCs enhances their proliferation rate^1^. Also, the numbers of factors contributing to differentiation of cells (Sox-9, RUNX2) rose significantly when PRP was added, comparing to a control group. Another in vitro study outlined that PRP accelerated MSCs proliferation. The effect was dose dependent and 10% PRP was sufficient to induce a marked cell proliferation^2^. Also, an important finding was that upon treatment with 10% PRP, cells entered logarithmic growth and removal of PRP restored the characteristic proliferation rate. That is an important finding for the in vivo translation, to avoid uncontrolled growth. One more study outlined that 10% PRP ratio brings to the ideal milieu for MSC proliferation^3^. An in vitro study again indicated that different concentrations of activated autologous PRP can promote cell proliferation at an earlier stage and promote osteogenic differentiation at later stages of adipose derived MSCs. It displayed a dose-dependent effect of activated autologous PRP on cell proliferation and osteogenic differentiation^4^. A systematic review of in vitro studies from 2014 draws a conclusion that adding PRP to a culture of cells increases the proliferation rate and migration of the cells and delays the appearance of the senescence phenotype^5^. This review also stated that 10% of PRP in cultures is optimal and increasing it to 30% did not enhance proliferation, on the contrary, it lowered it, compared to the commonly used FBS (Fetal Bovine Serum). All the findings in this review were about priming the cells before implantation. The safety of possible neoplasm growth was evaluated in the study where platelet lysate was added to prime the BM-MSCs. After clinical use, there were no tumors associated with use of these cells^6^. In an animal study, three concentrations of PRP (10%, 15% and 20%) were added to cultures of cells and compared. AT-MSCs pre-treated with or without PRP were transplanted into murine models of injured articular cartilage. The results showed that there was a strong difference between 15% and 20% PRP compared to 10% PRP and FBS, but no significant difference between 15% and 20%, drawing a conclusion that 15% is an ideal ratio of PRP in the culture. Another study compared 1, 5, 10, 20, 40 and 60% PRP and the results favored the 20% PRP as the most promising for cell proliferation rate^7^. In vitro, cultures treated with PRP enhanced factors associated with chondrocyte differentiation, while in animal study, in mice, cartilage regeneration was improved with PRP primed cells^8^. Not only the cells, but the host tissue can be primed to modulate the hostile conditions. In vitro study showed that PRP can modulate cells of expressing less metalloproteinases^9^.

*Clinical findings*

A high-quality study^10^ compared BM-MSCs with and without PRP. The results showed no statistical differences between these groups in KOOS score at 12-month end point, with both groups showing improvements vs baseline. Another study^11^ from the same authors compared similar groups (BM-MSCs with and without PRP) to corticosteroid injection. The results at 12 months showed significant improvement of both MSCs group compared to corticosteroid group in KOOS global score, but again no significant difference between them.

A recent meta-analysis showed that MSCs combined with PRP had more advantages in reducing the VAS score of patients with knee OA at 6 and 12 months after treatment; MSCs + PRP also showed better clinical efficacy than control group in improving the total WOMAC score of patients with knee OA 3 months and 6 months after treatment, but no significant difference after 12 months. There was no clear benefit over MSCs alone in adverse reactions^12^. The limitation of this systematic review is due to heterogeneity of control groups. Six studies were pooled in meta-analysis, and control group in each study was not the same (two studies with MSCs alone, two studies with HA, one study with PRP and one study with three control groups – MSCs, HA and PRP), so the small number of trials also had to be divided in three groups, limiting the statistical power.

A recent Level 2 randomized double-blind comparative parallel-group trial^13^ compared a single MFAT injection mixed or not with PRP Low Dose (LD) or High Dose (HD) in terms of clinical and MRI parameters, in knee OA patients. While at MRI no significantly changes over the time were found for any of the groups, all treatments significantly improved knee functional status and symptom relief at 3 and 6 months. No clinical benefit was observed by the addition of PRP to MFAT.

**References**

1. Mishra A, Tummala P, King A, et al. Buffered platelet-rich plasma enhances mesenchymal stem cell proliferation and chondrogenic differentiation. *Tissue Eng Part C Methods*. 2009;15(3):431-435. doi:10.1089/ten.tec.2008.0534

**Study type: *in vitro* study**

2. Lucarelli E, Beccheroni A, Donati D, et al. Platelet-derived growth factors enhance proliferation of human stromal stem cells. *Biomaterials*. 2003;24(18):3095-3100. doi:10.1016/s0142-9612(03)00114-5

**Study type: *in vitro* study**

3. Mardani M, Kabiri A, Esfandiari E, et al. The effect of platelet rich plasma on chondrogenic differentiation of human adipose derived stem cells in transwell culture. *Iran J Basic Med Sci*. 2013;16(11):1163-1169.

**Study type: *in vitro* study**

4. Xu FT, Li HM, Yin QS, et al. Effect of activated autologous platelet-rich plasma on proliferation and osteogenic differentiation of human adipose-derived stem cells in vitro. *Am J Transl Res*. 2015;7(2):257-270.

**Study type: *in vitro* study**

5. Rubio-Azpeitia E, Andia I. Partnership between platelet-rich plasma and mesenchymal stem cells: in vitro experience. *Muscles Ligaments Tendons J*. 2014;4(1):52-62.

**Study type: Systematic review; Level of evidence 2.**

6. Centeno CJ, Schultz JR, Cheever M, Robinson B, Freeman M, Marasco W. Safety and complications reporting on the re-implantation of culture-expanded mesenchymal stem cells using autologous platelet lysate technique. *Curr Stem Cell Res Ther*. 2010;5(1):81-93. doi:10.2174/157488810790442796

**Study type: Observational Study; Level of evidence 3.**

7. Atashi F, Jaconi MEE, Pittet-Cuénod B, Modarressi A. Autologous platelet-rich plasma: a biological supplement to enhance adipose-derived mesenchymal stem cell expansion. *Tissue Eng Part C Methods*. 2015;21(3):253-262. doi:10.1089/ten.TEC.2014.0206

**Study type: *in vitro* study**

8. Van Pham P, Bui KHT, Ngo DQ, et al. Activated platelet-rich plasma improves adipose-derived stem cell transplantation efficiency in injured articular cartilage. *Stem Cell Res Ther*. 2013;4(4):91. doi:10.1186/scrt277

**Study type: *In vivo* studies.**

9. Wang CC, Lee CH, Peng YJ, Salter DM, Lee HS. Platelet-Rich Plasma Attenuates 30-kDa Fibronectin Fragment-Induced Chemokine and Matrix Metalloproteinase Expression by Meniscocytes and Articular Chondrocytes. *Am J Sports Med*. 2015;43(10):2481-2489. doi:10.1177/0363546515597489

**Study type: *in vitro* study**

10. Bastos R, Mathias M, Andrade R, et al. Intra-articular injections of expanded mesenchymal stem cells with and without addition of platelet-rich plasma are safe and effective for knee osteoarthritis. *Knee Surg Sports Traumatol Arthrosc*. 2018;26(11):3342-3350. doi:10.1007/s00167-018-4883-9

**Study type: RCT; Level of evidence 2.**

11. Bastos R, Mathias M, Andrade R, et al. Intra-articular injection of culture-expanded mesenchymal stem cells with or without addition of platelet-rich plasma is effective in decreasing pain and symptoms in knee osteoarthritis: a controlled, double-blind clinical trial. *Knee Surg Sports Traumatol Arthrosc*. 2020;28(6):1989-1999. doi:10.1007/s00167-019-05732-8

**Study type: RCT; Level of evidence 2.**

12. Zhao J, Liang G, Han Y, et al. Combination of mesenchymal stem cells (MSCs) and platelet-rich plasma (PRP) in the treatment of knee osteoarthritis: a meta-analysis of randomised controlled trials. *BMJ Open*. 2022;12(11):e061008. doi:10.1136/bmjopen-2022-061008

**Study type: Systematic Review and Meta-analysis; Level of evidence 2.**

13. Louis ML, Dumonceau RG, Jouve E, et al. Intra-Articular Injection of Autologous Microfat and Platelet-Rich Plasma in the Treatment of Knee Osteoarthritis: A Double-Blind Randomized Comparative Study. *Arthroscopy*. 2021;37(10):3125-3137.e3. doi:10.1016/j.arthro.2021.03.074

**Study type: RCT; Level of evidence 2.**
